# Ellagic Acid and Its Metabolites as Potent and Selective Allosteric Inhibitors of Liver Pyruvate Kinase

**DOI:** 10.3390/nu15030577

**Published:** 2023-01-22

**Authors:** Umberto Maria Battisti, Chunixa Gao, Fady Akladios, Woonghee Kim, Hong Yang, Cemil Bayram, Ismail Bolat, Metin Kiliclioglu, Nursena Yuksel, Ozlem Ozdemir Tozlu, Cheng Zhang, Jihad Sebhaoui, Shazia Iqbal, Saeed Shoaie, Ahmet Hacimuftuoglu, Serkan Yildirim, Hasan Turkez, Mathias Uhlen, Jan Boren, Adil Mardinoglu, Morten Grøtli

**Affiliations:** 1 Department of Chemistry and Molecular Biology, University of Gothenburg, 412 96 Gothenburg, Sweden; 2 Science for Life Laboratory, KTH–Royal Institute of Technology, 104 50 Stockholm, Sweden; 3 Department of Medical Pharmacology, Faculty of Medicine, Atatürk University, Erzurum 25240, Turkey; 4 Department of Pathology, Faculty of Veterinary, Atatürk University, Erzurum 25240, Turkey; 5 Department of Molecular Biology and Genetics, Faculty of Science, Erzurum Technical University, Erzurum 25050, Turkey; 6 School of Pharmaceutical Sciences, Zhengzhou University, Zhengzhou 450001, China; 7 Trustlife Labs, Drug Research & Development Center, Istanbul 34774, Turkey; 8 Centre for Host-Microbiome Interactions, Faculty of Dentistry, Oral & Craniofacial Sciences, King’s College London, London SE1 9RT, UK; 9 Department of Medical Biology, Faculty of Medicine, Atatürk University, Erzurum 25240, Turkey; 10 Department of Molecular and Clinical Medicine, University of Gothenburg, 405 30 Gothenburg, Sweden; 11 Sahlgrenska University Hospital, 405 30 Gothenburg, Sweden

**Keywords:** NAFLD, liver pyruvate kinase, ellagic acid, urolithins, enzyme inhibition

## Abstract

Liver pyruvate kinase (PKL) has recently emerged as a new target for non-alcoholic fatty liver disease (NAFLD), and inhibitors of this enzyme could represent a new therapeutic option. However, this breakthrough is complicated by selectivity issues since pyruvate kinase exists in four different isoforms. In this work, we report that ellagic acid (EA) and its derivatives, present in numerous fruits and vegetables, can inhibit PKL potently and selectively. Several polyphenolic analogues of EA were synthesized and tested to identify the chemical features responsible for the desired activity. Molecular modelling studies suggested that this inhibition is related to the stabilization of the PKL inactive state. This unique inhibition mechanism could potentially herald the development of new therapeutics for NAFLD.

## 1. Introduction

In the past decades, lifestyle modifications have drastically affected health priorities in several areas of the world, and emerging pathologies have changed the medical landscape. Non-alcoholic fatty liver disease (NAFLD) is a new chronic liver disease and is strongly associated with the worldwide increase in obesity [1]. NAFLD is an umbrella term that includes several liver abnormalities such as excess triglyceride accumulation, ballooning degeneration, inflammation, fibrosis, and cirrhosis [2]. Almost 33% of adults in the U.S. are affected by hepatic steatosis, even though this number might be even higher [3]. It is estimated that 15–20% of these individuals will develop non-alcoholic steatohepatitis, with 15–20% of these further progressing to cirrhosis [4]. Due to these alarming data, NAFLD has been recognized as an important cause of liver disease, and will likely grow into the leading cause of end-stage liver disease within the next decade. Despite the alarming prevalence of NAFLD, no therapy has yet been approved; the only available option has been weight loss (e.g., calorie restriction, exercise, etc.), and possibly vitamin E supplementation [5]. Therefore, it is crucial to discover and validate novel biomarkers and drug targets for the early diagnosis and effective treatment of NAFLD [6]. We recently employed an innovative approach based on integrative network analyses to identify new targets for NAFLD treatment with minimal side effects [7]. This study suggested that downregulation and/or an inhibition of liver pyruvate kinase (PKL) could serve as a successful strategy to block and even reverse NAFLD progression. Krishnan et al. reached a similar conclusion employing in vivo and in vitro knock-out models [8]. PKL has also been identified as one of the driver genes responsible for NAFLD, and is therefore a key target for the development of treatment strategies [9].

Pyruvate kinase (PK) is an enzyme that catalyzes the final step in glycolysis, converting phosphoenolpyruvate (PEP) and adenosine diphosphate (ADP) to pyruvate (PYR) and adenosine triphosphate (ATP). There are four mammalian pyruvate kinase isoforms, PKM1, PKM2, PKR, and PKL, and each isoform has had multiple designations over time in the literature [10]. Although a tissue may express more than one pyruvate kinase isoform, individual cells generally express only one at appreciable levels. Most adult tissues express PKM2, and expression of the other three isoforms is restricted to distinct tissues and cell types [11,12,13]. The PKL isoform is expressed in the liver, pancreatic β-cells, small intestine, and renal proximal tubule [14]. PKL inhibitors may be useful in halting NAFLD progression [7], but potent and selective inhibitors have not been reported in the literature. However, it is well known that some natural molecules, in particular polyphenolic compounds, potently inhibit the PKM2 isoform [15,16,17]. Most of these polyphenols have been found to possess a variety of pharmacological effects on oxidative stress, lipid metabolism, insulin resistance, and inflammation, which are all important pathological processes in the etiology of liver diseases such as NAFLD [18,19,20,21]. The mechanisms underlying the beneficial effects of many polyphenols on NAFLD have been extensively studied in recent years. In addition to the indirect antioxidant and anti-inflammatory effects and regulation of classical intracellular signal transduction, it has been demonstrated that some polyphenols exert their therapeutic effects through emerging new mechanisms (e.g., reduction of de novo lipogenesis and increased fatty acid β-oxidation) [18]. For these reasons, we hypothesized that some polyphenols might be beneficial in NAFLD due to PKL inhibition. EA is a naturally occurring polyphenolic compound found in numerous fruits, nuts, and seeds, such as pomegranates, grapes, raspberries, and walnuts, with antioxidative, anti-obesity, hypolipidemic, and antidiabetic effects [22]. Furthermore, EA has been reported to be an ATP competitive inhibitor of protein kinase CK2 [23]. Therefore, we aimed to test EA and urolithins, EA’s metabolites, to evaluate their inhibitory activity against PKL.

## 2. Materials and Methods

### 2.1. Materials

**Scheme 1:**
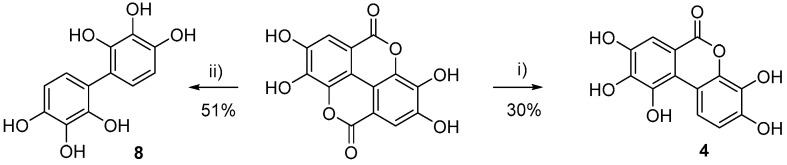
Synthesis of 4 and 8 from ellagic acid. Reagents and conditions: (i) KOH, H_2_O, 100 °C, 2 h; (ii) KOH, H_2_O, 170 °C, 2 h.

**Scheme 2:**
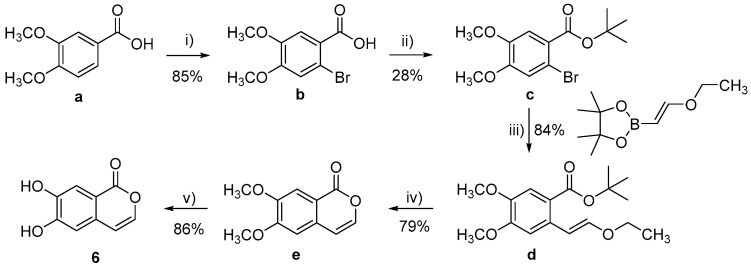
Synthesis of 6. Reagents and conditions: (i) Br_2_, HCl, rt, 2 h; (ii) *t*-BuOH, MgSO_4_, H_2_SO_4_, CH_2_Cl_2_, rt, 48 h; (iii) Na_2_CO_3_, Pd(PPh_3_)_3_, H_2_O, Dioxane, 110 °C, 2 h; (iv) TFA, 100 °C, 2 h; (v) BBr_3_, CH_2_Cl_2_, rt, 12 h.

**Scheme 3:**
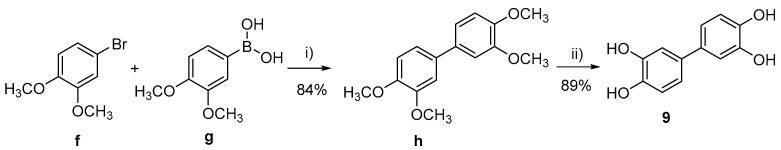
Synthesis of 9. Reagents and conditions: (i) Na_2_CO_3_, Pd(PPh_3_)_3_, H_2_O, Dioxane, 110 °C, 2 h; (ii) BBr_3_, CH_2_Cl_2_, rt, 12 h.

**Scheme 4:**
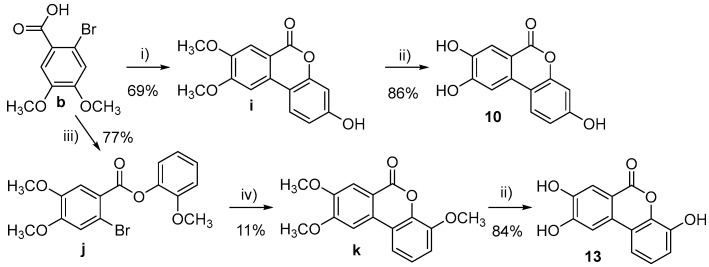
Synthesis of 10 and 13. Reagents and conditions: (i) NaOH, Na_2_CO_3_, resorcinol, CuI, H_2_O, 50 °C, 12 h; (ii) BBr_3_, CH_2_Cl_2_, rt, 12 h; (iii) SOCl_2_, reflux, 2 h, then Et_3_N, 2-methoxyphenol, CH_2_Cl_2_, rt, 12 h; (iv) NaOAc, Cy_3_P·HBF_4_, Pd(OAc)_2_, DMF, reflux, 72 h.

**Scheme 5:**
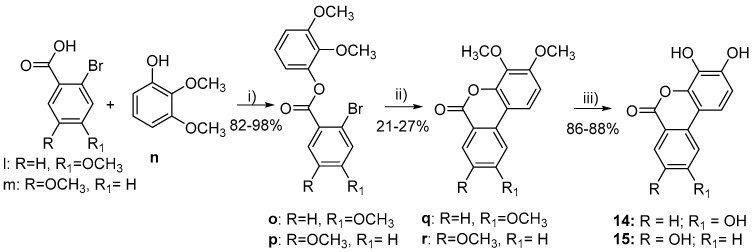
Synthesis of 14 and 15. Reagents and conditions: (i) SOCl_2_, reflux, 2 h, then Et_3_N, CH_2_Cl_2_, rt, 12 h; (ii) NaOAc, Cy_3_P·HBF_4_, Pd(OAc)_2_, DMF, reflux, 72 h; (iii) BBr_3_, CH_2_Cl_2_, rt, 12 h.

Compounds 1–3 and 7 were purchased from Sigma-Aldrich, Stockholm, Swedenwhile compounds 5, 11, and 12 were purchased from Toronto Research Chemicals (20 Martin Ross Avenue, North York, M3J 2K8, Toronto, ON, Canada). All other target molecules (4, 6, 8–10, and 13–15) were synthesized in-house (Scheme 1, Scheme 2, Scheme 3, Scheme 4 and Scheme 5).

3,4,8,9,10-Pentahydroxy-6H-benzo[c]chromen-6-one (**4**)

The compound was synthesized according to the literature procedure [24]. A solution of ellagic acid (0.100 g, 0.33 mmol) in aqueous KOH (0.8 g, 5.0 mL) was heated at 100 °C for 20 min. The mixture was then cooled to rt and acidified (pH 1). The aqueous phase was extracted with Et_2_O (3 × 20 mL). The combined organic layers were dried (Na_2_SO_4_), filtered, and concentrated to give a yellow powder. The solid obtained was recrystallized from water to give 0.025 g (30%) of the target compound as a grey solid. m.p. = 230–232 °C, ^1^H-NMR (CD_3_OD, 400 MHz): 6.75 (d, J = 9.0 Hz, 1H, Ar-H), 7.37 (s, 1H, Ar-H), 8.44 (d, J = 9.0 Hz, 1H, Ar-H); ^13^C-NMR (CD_3_OD, 100 MHz): 106.6, 110.7, 111.0, 111.4, 117.1, 117.7, 131.9, 139.6, 140.3, 142.6, 145.0, 145.4, 162.3; HRMS: found M-H: m/z 275.0206, calculated value for C_13_H_7_O_7_-: 275.0197 (delta: −3.3 ppm).

2-Bromo-4,5-Dimethoxybenzoic Acid (**b**)

The compound was synthesized according to the literature procedure [25]. To a suspension of 3,4-dimethoxybenzoic acid (a, 5.00 g, 27.44 mmol) in HCl conc. (100 mL), bromine (4.82 g, 30.19 mmol) was added dropwise at rt. The reaction mixture was stirred for 2 h at rt. Water (100 mL) was then added, and the resulting precipitate was collected by filtration, and recrystallized from MeOH to give 6.1 g (85%) of 2-bromo-3,4-dimethoxybenzoic acid as a white solid. m.p. = 185–187 °C, ^1^H-NMR (DMSO-*d*_6_, 400 MHz): 3.76 (s, 3H, CH_3_), 3.81 (s, 3H, CH_3_), 7.16–7.20 (m, 1H. Ar-H), 7.31–7.37 (m, 1H, Ar-H); ^13^C-NMR (DMSO-*d*_6_, 100 MHz): 56.1, 56.2, 112.8, 114.2, 124.4, 148.0, 151.9, 166.9.

*tert*-Butyl 2-bromo-4,5-dimethoxybenzoate (**c**)

The compound was synthesized according to the literature procedure [26]. To a solution of 2-bromo-3,4-dimethoxybenzoic acid (**b**, 1.30 g, 5.00 mmol) in CH_2_Cl_2_, MgSO_4_ (2.41 g, 20 mmol) was added. The flask was then purged with nitrogen and H_2_SO_4_ (0.46 g, 4.75 mmol) and tert-butanol (1.85 g, 25.00 mmol) was added. The reaction was stirred at rt for 48 h. The reaction was quenched by addition of EtOAC/pentane (20 mL, 50/50), followed by saturated NaHCO_3_ solution. The layers were separated, and the organic layer was dried (Na_2_SO_4_), filtered, and concentrated in vacuum. The crude mixture was purified by flash chromatography (90/10 pentane/EtOAc) to give 0.41 g (26%) of *tert*-butyl 2-bromo-4,5-dimethoxybenzoate as a colorless oil. ^1^H-NMR (CDCl_3_, 400 MHz) = 1.56 (s, 9H, 3 x CH_3_), 3.84 (s, 3H, CH_3_), 3.86 (s, 3H, CH_3_), 7.00 (s, 1H, Ar-H), 7.27 (s, 1H, Ar-H); ^13^C-NMR (CDCl3, 400 MHz) = 28.1, 56.0, 56.2, 82.2, 113.1, 113.8, 116.6, 125.2, 147.7, 151.4, 165.0.

(E)-*tert*-Butyl 2-(2-ethoxyvinyl)-4,5-dimethoxybenzoate (**d**)

The compound was synthesized according to the literature procedure [27]. *tert*-Butyl 2-bromo-4,5-dimethoxybenzoate (0.10 g, 0.31 mmol), trans-2-ethoxyvinylboronicacid pinacol ester (0.06 g, 0.31 mmol), palladium tetrakis (0.072 g, 0.06 mmol), and sodium carbonate (0.10 g, 0.94 mmol) were placed in a 5.0 mL microwave vial. A degassed solution of dioxane/water (50:50, 4 mL) was added. The mixture was stirred for 2 h at 110 °C in the microwave. The solution was then rinsed with water (20.0 mL) and extracted with EtOAc (3 × 20.0 mL). The organic phase was dried (Na_2_SO_4_), filtered, and concentrated in vacuum to give 0.12 g of crude product. Purification by flash chromatography (90/10 pentane/EtOAc) afforded 0.070 g (72%) of the target compound as a yellow oil. ^1^H-NMR (CDCl_3_, 400 MHz): 1.34 (t, J = 7.03, 3H, CH_3_), 1.58 (s, 9H, C(CH_3_)_3_), 3.84–3.96 (m, 8H, CH_2_, 2 x CH_3_), 6.69 (d, J = 12.9 Hz, 1H, CH), 6.78 (s, 1H, Ar-H), 6.81 (d, J = 12.9 Hz, 1H, CH), 7.38 (s, 1H, Ar-H); ^13^C-NMR (CDCl_3_, 100 MHz): 14.8, 28.3, 55.8, 55.9, 65.1, 80.8, 105.4, 108.6, 113.5, 121.4, 132.1, 146.7, 148.2, 151.5, 166.6.

6,7-Dimethoxy-1H-isochromen-1-one (**e**)

The compound was synthesized according to the literature procedure [27]. A solution of (E)-*tert*-butyl 2-(2-ethoxyvinyl)-4,5-dimethoxybenzoate (0.640 g, 2.07 mmol) in TFA (14.0 mL) in a sealed microwave reactor was heated at 100 °C for 2 h under microwaved-assisted conditions. The reaction mixture was evaporated to dryness. The residue was purified by flash chromatography (70/30 pentane/EtOAc) to give 0.34 g (79%) of the target compound as a yellow solid. m.p. = 116–118 °C; ^1^H-NMR (CDCl_3_, 400 MHz): 3.88 (s, 3H, CH_3_), 3.91 (s, 3H, CH_3_), 6.35 (d, J = 5.6 Hz, 1H, CH), 6.72 (s, 1H, Ar-H), 7.15 (d, J = 5.6 Hz, 1H, CH), 7.53 (s, 1H, Ar-H); ^13^C-NMR (CDCl3, 100 MHz): 56.1, 56.2, 106.2, 106.6, 109.3, 114.8, 132.0, 143.7, 149.7, 155.0, 162.1.

6,7-dihydroxy-1H-isochromen-1-one (**6**)

To a solution of 6,7-dimethoxy-1H-isochromen-1-one (0.270 g, 1.31 mmol) in CH_2_Cl_2_ (30.0 mL), BBr_3_ in CH_2_Cl_2_ (1.0 M, 5.76 mmol) was added dropwise at 0 °C. The solution was stirred at rt for 2 h. Water (20.0 mL) was added to quench the reaction and CH_2_Cl_2_ was removed under vacuum. The aqueous layer was extracted with ethyl acetate (3 × 20.0 mL). The combined organic phase was dried (Na_2_SO_4_), filtered, and concentrated in vacuum. The solid obtained was triturated in pentane and filtered to afford 0.200 g (86%) of the target compound, as a dark brown solid. m.p. = 265–267 °C; ^1^H-NMR (DMSO-*d*_6_, 400 MHz): 6.57 (d, J = 5.6 Hz, 1H, CH), 6.88 (s, 1H, Ar-H), 7.34 (d, J = 5.6 Hz, 1H, CH), 7.42 (s, 1H, Ar-H), 9.96 (br s, 1H, OH), 10.40 (br s, 1H, Ar-H); ^13^C-NMR (DMSO-*d*_6_, 100 MHz): 106.9, 111.1, 113.5, 113.6, 131.1, 143.7, 147.3, 153.5, 161.7; HRMS: found M-H: m/z 177.0198, calculated value for C_9_H_6_O_4_-: 177.0193 (delta: −2.8 ppm).

[1,1’-Biphenyl]-2,2’,3,3’,4,4’-hexaol (**8**)

The compound was synthesized according to the literature procedure [24]. A solution of ellagic acid (0.500 g, 1.65 mmol) in aqueous NaOH (5 M, 5.0 mL) was heated at 170 °C for 2 h. The mixture was then cooled to rt and acidified (pH 1). The solid obtained was filtered and recrystallized from water to give 0.21 g (51%) of [1,1’-biphenyl]-2,2’,3,3’,4,4’-hexaol as a brown solid. m.p. >350 °C, ^1^H-NMR (DMSO-*d*_6_, 400 MHz): 6.32 (d, J = 8.4 Hz, 2H, Ar-H), 6.40 (d, J = 8.4 Hz, 2H, Ar-H); ^13^C-NMR (DMSO-*d*_6_, 100 MHz): 107.8, 119.0, 121.0, 133.7, 143.4, 145.3; HRMS: found M-H: m/z 249.0411, calculated value for C_12_H_9_O_6_^−^: 249.0405 (delta: 2.1 ppm).

3,3’,4,4’-tetramethoxy-1,1’-biphenyl (**h**)

The compound was synthesized according to the literature procedure [28]. 4-Bromoveratrole (**f**, 0.50 g, 2.30 mmol), 3,4-dimethoxybenzeneboronic acid (g, 0.46 g, 2.53 mmol), palladium tetrakis (0.27 g, 0.23 mmol), and potassium carbonate (1.27 g, 9.20 mmol) were placed in a 20.0 mL microwave vial. A degassed solution of dioxane/water (50:50, 12.0 mL) was added. The mixture was stirred for 1 h at 110 °C in the microwave. The solution was then rinsed with water (20.0 mL) and extracted with EtOAc (3 × 20.0 mL). The organic phase was dried (Na2SO4), filtered, and concentrated in a vacuum to give 0.7 g of crude product. Purification by flash chromatography (70/30 pentane/EtOAc) afforded 0.53 g (84%) of the target compound as a white solid. m.p. = 133–135 °C, ^1^H-NMR (CDCl_3_, 400 MHz): 3.85 (s, 6H, CH_3_), 3.89 (s, 6H, CH_3_), 6.86 (d, 2H, J = 8.1 Hz, Ar-H), 7.02–7.07 (m, 4H, Ar-H); ^13^C-NMR (CDCl_3_, 100 MHz): 55.9, 110.3, 111.5, 119.2, 134.1, 148.3, 149.1.

[1,1’-biphenyl]-3,3’,4,4’-tetraol (**9**)

The compound was synthesized according to the literature procedure [29]. To a solution of 3,3’,4,4’-tetramethoxy-1,1’-biphenyl (**h**, 0.415 g, 1.51 mmol) in CH_2_Cl_2_ (30.0 mL), BBr_3_ in CH_2_Cl_2_ (1.0 M, 18.15 mmol) was added dropwise at 0 °C. The solution was stirred at rt for 2 h. Water was added to quench the reaction and CH_2_Cl_2_ was removed under vacuum. The aqueous layer was extracted with ethyl acetate (3 × 30 mL). The combined organic phase was dried (Na_2_SO_4_), filtered, and concentrated in vacuum. The solid obtained was triturated in pentane and filtered to afford 0.29 g (89%) of the target compound as a white solid. m.p. = 232–234 °C; ^1^H-NMR (CD_3_OD, 400 MHz): 6.76 (d, J = 8.2 Hz, 2H, Ar-H), 6.83 (dd, J = 2.2, 8.2 Hz, 2H, Ar-H), 144.9; HRMS: found M-H: m/z 217.0514, calculated value for C_12_H_9_O_4_-: 217.0506 (delta: −3.7 ppm).

3-Hydroxy-8,9-dimethoxy-6H-benzo[c]chromen-6-one (**i**)

The compound was synthesized according to the literature procedure [30]. To a suspension of 2-bromo-3,4-dimethoxybenzoic acid (b, 1.04 g, 4.00 mmol) and resorcinol (1.32 g, 12.00 mmol) in H_2_O (10.0 mL), NaOH solution (4 M, 1.0 mL) was added. The solution was stirred at rt until complete dissolution and subsequently Na_2_CO_3_ (0.93 g, 8.80 mmol) was added. The mixture was stirred at 50 °C for 10 min and then CuI (0.23 g, 1.20 mmol) was added. The resulting suspension was stirred at 50 °C for 12 h. The precipitate formed was filtered, washed with CH_3_OH, dried and triturated with Et_2_O to give 0.75 g (69%) of the target compound as a white solid. m.p. = 329–331 °C; ^1^H-NMR (DMSO-*d*_6_, 400 MHz): 3.85 (s, 3H, CH_3_), 3.98 (s, 3H, CH_3_), 6.60–6.93 (m, 2H, Ar-H), 7.41–7.69 (m, 2H, Ar-H), 8.15 (s, 1H, Ar-H), 10.19 (br s, 1H, OH); ^13^C-NMR (DMSO-*d*_6_, 100 MHz): 56.0, 56.6, 103.1, 103.7, 109.9, 112.0, 113.2, 125.0, 130.7, 149.2, 152.1, 155.5, 159.4, 160.7.

3,8,9-trihydroxy-6H-benzo[c]chromen-6-one (**10**)

To a solution of 3-hydroxy-8,9-dimethoxy-6H-benzo[c]chromen-6-one (**i**, 0.100 g, 0.37 mmol) in CH_2_Cl_2_ (10.0 mL), BBr_3_ in CH_2_Cl_2_ (1.0 M, 2.42 mmol) was added dropwise at 0 °C. The solution was stirred at rt for 48 h. MeOH was added to quench the reaction and the solvent was removed under vacuum. The solid obtained was triturated in Et_2_O and filtered to afford 0.076 g (85%) of the target compound as a white solid. m.p. > 350 °C; ^1^H-NMR (DMSO-d6, 400 MHz): 6.62 (d, J = 2.0 Hz, 1H, Ar-H), 6.75 (dd, J = 2.0, 8.6 Hz, 1H, Ar-H), 7.39 (s, 1H, Ar-H), 7.45 (s, 1H, Ar-H), 7.80 (d, J = 8.6 Hz, 1H, Ar-H), 10.17 (br s, 3H, OH); ^13^C-NMR (DMSO-*d*_6_, 100 MHz): 103.2, 107.3, 110.2, 111.3, 113.3, 114.6, 124.1, 129.6, 146.5, 151.9, 153.8, 159.0, 160.7; HRMS: found M-H: m/z 243.0306, calculated value for C_13_H_7_O_5_-: 243.0299 (delta: −2.9 ppm).

2-Methoxyphenyl 2-bromo-4,5-dimethoxybenzoate (**j**)

A solution of 2-bromo-3,4-dimethoxybenzoic acid (**b**, 1.30 g, 4.33 mmol) in thionyl chloride (10.0 mL) was refluxed for 2 h, and then the solvent was removed in vacuo. The oil obtained was solubilized in CH_2_Cl_2_ (10 mL), and added dropwise to a solution of 2-methoxyphenol (0.680 g, 5.48 mmol) and Et3N (0.75 g, 7.47 mmol) in CH_2_Cl_2_ (10.0 mL). The mixture was stirred at rt for 2 h and concentrated in vacuo. Purification by flash chromatography (80/20 Hexane/EtOAc) afforded 1.41 g (77%) of 2-methoxyphenyl 2-bromo-4,5-dimethoxybenzoate as a colorless oil. ^1^H-NMR (CDCl_3_, 400 MHz): 3.84 (s, 3H, CH_3_), 3.94 (s, 3H, CH_3_), 3.95 (s, 3H, CH_3_), 6.95–7.04 (m, 2H, Ar-H), 7.15–7.20 (m, 2H, Ar-H), 7.21–7.27 (m, 1H, Ar-H), 7.68 (s, 1H, Ar-H); ^13^C-NMR (CDCl_3_, 100 MHz): 55.9, 56.2, 56.3, 112.5, 114.5, 115.3, 117.1, 120.8, 121.9, 123.0, 127.0, 139.8, 147.8, 151.2, 152.5, 163.2.

4,8,9-Trimethoxy-6H-benzo[c]chromen-6-one (**k**)

To a solution of 2-methoxyphenyl 2-bromo-4,5-dimethoxybenzoate (1.30 g, 3.54 mmol) in dry DMF (10.0 mL), NaOAc (0.58 g, 7.08 mmol), Pd(OAc)_2_ (0.07 g, 0.35 mmol), and tricyclohexylphosphine tetrafluoroborate (0.39 g, 1.06 mmol) were added. The mixture was degassed and then refluxed under nitrogen for 3 days. The reaction mixture was cooled to rt, and then diluted with diethyl ether and filtered through Celite to remove the catalyst. The solvent was removed under vacuum to give 1.1 g of a dark oil. Crystallization from MeOH afforded 0.11 g (11%) of the target compound as a white solid. m.p. = 222–224 °C; ^1^H-NMR (DMSO-*d*_6_, 400 MHz): 3.89 (s, 3H, CH_3_), 3.90 (s, 3H, CH_3_), 4.01 (s, 3H, CH_3_), 7.17 (dd, J = 1.3, 8.2 Hz, 1H, Ar-H), 7.29 (app t, J = 8.1 Hz, 1H, Ar-H), 7.57 (s, 1H, Ar-H), 7.75 (s, 1H, Ar-H), 7.90 (dd, J = 1.3, 8.3 Hz, 1H, Ar-H); ^13^C-NMR (DMSO-*d*_6_, 100 MHz): 56.2, 56.4, 56.8, 105.0, 110.1, 112.4, 113.8, 115.2, 118.9, 124.6, 130.1, 140.2, 147.6, 150.4, 155.5, 160.0.

4,8,9-Trihydroxy-6H-benzo[c]chromen-6-one (**13**)

To a solution of 4,8,9-trimethoxy-6H-benzo[c]chromen-6-one (0.07 g, 0.24 mmol) in CH_2_Cl_2_ (20 mL), BBr_3_ in CH_2_Cl_2_ (1.0 M, 2.20 mmol) was added dropwise at 0 °C. The solution was stirred at rt for 12 h. Water (20 mL) was added to quench the reaction and the aqueous layer was extracted with EtOAc (3 × 20 mL). The organic phase was dried (Na_2_SO_4_), filtered, and concentrated in vacuo to give a brown solid. The solid obtained was triturated in Et_2_O and filtered to afford 0.05 g (84%) of the target compound as a pink powder. m.p. = 345–347 °C; ^1^H-NMR (MeOD, 400 MHz): 6.93 (dd, J = 1.4, 8.0 Hz, 1H, Ar-H), 7.13 (t, J = 8.0 Hz, 1H, Ar-H), 7.45 (dd, J = 1.4, 8.0 Hz, 1H, Ar-H), 7.50 (s, 1H, Ar-H), 7.63 (s, 1H, Ar-H); ^13^C-NMR (MeOD, 100 MHz): 107.2, 112.4, 112.8, 114.0, 115.2, 119.1, 124.0, 129.6, 139.3, 145.1, 147.0, 153.3, 161.2; HRMS: found M-H: m/z 243.0308, calculated value for C_13_H_7_O_5_-: 243.0299 (delta: −3.7 ppm).

2,3-Dimethoxyphenyl 2-bromo-4-methoxybenzoate (**o**)

A solution of 2-bromo-4-methoxybenzoic acid (l, 1.0 g, 4.33 mmol) in thionyl chloride (10.0 mL) was refluxed for 2 h, and then the solvent was removed in vacuo. The oil obtained was solubilized in CH_2_Cl_2_ (10.0 mL) and added dropwise to a solution of 2,3-dimethoxyphenol (0.73 g, 4.76 mmol), and Et_3_N (0.66 g, 6.49 mmol) in CH_2_Cl_2_ (10 mL). The mixture was stirred at rt for 2 h and concentrated in vacuo. Purification by flash chromatography (80/20 Hexane/EtOAc) afforded 1.31 g (82%) of the target compound as a colorless oil. ^1^H-NMR (CDCl_3_, 400 MHz): 3.86 (s, 6H, 2 x CH3), 3.88 (s, 3H, CH3), 6.81 (dd, J = 1.5, 8.2 Hz, 1H, Ar-H), 6.84 (dd, J = 1.5, 8.24 Hz, 1H, Ar-H), 6.93 (dd, J = 2.5, 8.8 Hz, 1H, Ar-H), 7.06 (t, J = 8.3 Hz, 1H, Ar-H), 7.25 (d, J = 2.5 Hz, 1H, Ar-H), 8.13 (d, J = 8.8 Hz, 1H, Ar-H); ^13^C-NMR (CDCl_3_, 100 MHz): 55.8, 56.1, 60.9, 110.3, 113.1, 115.3, 120.1, 122.4, 123.5, 124.0, 124.4, 134.0, 141.3, 144.2, 153.8, 162.3, 168.4.

3,4,9-Trimethoxy-6H-benzo[c]chromen-6-one (**q**)

To a solution of 2,3-dimethoxyphenyl 2-bromo-4-methoxybenzoate (**o**, 1.30 g, 3.54 mmol) in dry DMF (10.0 mL), NaOAc (0.58 g, 7.08 mmol), Pd(OAc)2 (0.07 g, 0.35 mmol), and tricyclohexylphosphine tetrafluoroborate (0.39 g, 1.06 mmol) were added. The mixture was degassed and then refluxed under nitrogen for 3 days. The reaction mixture was cooled to rt, and then diluted with diethyl ether and filtered through Celite to remove the catalyst. The solvent was under vacuum to give 1.1 g of a dark oil. Crystallization from MeOH afforded 0.270 g (27%) of the target compound as a white solid. m.p. = 183–185 °C; ^1^H-NMR (DMSO-*d*_6_, 400 MHz): 3.82 (s, 3H, CH_3_), 3.90 (s, 3H, CH_3_), 3.96 (s, 3H, CH_3_), 7.09 (d, J = 9.1 Hz, 1H, Ar-H), 7.13 (dd, J = 2.4, 8.2 Hz, 1H, Ar-H), 7.71 (d, J = 2.4 Hz, 1H, Ar-H), 8.06–8.12 (m, 2H, Ar-H); ^13^C-NMR (DMSO-*d*_6_, 100 MHz): 56.5, 56.7, 61.2, 105.6, 109.4, 112.3, 112.5, 116.7, 119.4, 132.5, 136.2, 137.7, 145.6, 154.5, 160.2, 166.2.

3,4,9-Trihydroxy-6H-benzo[c]chromen-6-one (**14**)

To a solution of 3,4,9-trimethoxy-6H-benzo[c]chromen-6-one (**q**, 0.15 g, 0.52 mmol) in CH_2_Cl_2_ (20 mL), BBr_3_ in CH_2_Cl_2_ (1.0 M, 4.71 mmol) was added dropwise at 0 °C. The solution was stirred at rt for 12 h. Water (20 mL) was added to quench the reaction and the aqueous layer was extracted with EtOAc (3 × 20 mL). The organic phase was dried (Na_2_SO_4_), filtered, and concentrated in vacuo to give a white powder. The solid obtained was triturated in Et2O and filtered to afford 0.110 g (86%) of the target compound as a brown solid. m.p. = 301–303 °C; ^1^H-NMR (DMSO-*d*_6_, 400 MHz): 6.80 (d, J = 8.7 Hz, 1H, Ar-H), 6.95 (dd, J = 2.2, 8.7 Hz, 1H, Ar-H), 7.40 (d, J = 2.2 Hz, 1H, Ar-H), 7.43 (d, J = 8.8 Hz, 1H, Ar-H), 8.03 (d, J = 8.8 Hz, 1H, Ar-H), 9.19 (br s, 1H, OH), 9.79 (br s, 1H, OH), 10.77 (br s, 1H, OH); ^13^C-NMR (DMSO-*d*_6_, 100 MHz): 106.8, 110.6, 110.9, 112.7, 113.7, 116.9, 132.9, 133.1, 138.3, 141.4, 148.4, 160.7, 164.1; HRMS: found M-H: m/z 243.0305, calculated value for C_13_H_7_O_5_-: 243.0299 (delta: −2.5 ppm).

2,3-Dimethoxyphenyl 2-bromo-5-methoxybenzoate (p)

A solution of 2-bromo-5-methoxybenzoic acid (m, 1.0 g, 4.33 mmol) in thionyl chloride (10.0 mL) was refluxed for 2 h and then the solvent removed in vacuo. The oil obtained was solubilized in CH_2_Cl_2_ (10.0 mL) and added dropwise to a solution of 2,3-dimethoxyphenol (0.73 g, 4.76 mmol), and Et_3_N (0.66 g, 6.49 mmol) in CH_2_Cl_2_ (10.0 mL). The mixture was stirred at rt for 2 h and concentrated in vacuo. Purification by flash chromatography (80/20 Hexane/EtOAc) afforded 1.56 g (98%) of the target compound as a colorless oil. ^1^H-NMR (CDCl_3_, 400 MHz): 3.85 (s, 3H, CH_3_), 3.88 (s, 3H, CH_3_), 3.90 (s, 3H, CH_3_), 6.81–6.88 (m, 2H, Ar-H), 6.96 (dd, J = 3.1, 8.8 Hz, 1H, Ar-H), 7.98 (t, J = 8.3 Hz, 1H, Ar-H), 7.58 (d, J = 3.1 Hz, 1H, Ar-H), 7.60 (d, J = 8.8 Hz, 1H, Ar-H); ^13^C-NMR (CDCl_3_, 100 MHz): 55.7, 56.1, 60.9, 110.5, 112.6, 115.0, 116.9, 119.6, 123.5, 131.8, 135.3, 141.2, 144.0, 153.8, 158.6, 164.0.

3,4,8-Trimethoxy-6H-benzo[c]chromen-6-one (r)

To a solution of 2,3-dimethoxyphenyl 2-bromo-5-methoxybenzoate (**p**, 1.00 g, 2.72 mmol) in dry DMF (10.0 mL), NaOAc (0.446 g, 5.47 mmol), Pd(OAc)2 (0.061, 0.27 mmol), and tricyclohexylphosphine tetrafluoroborate (0.300 g, 0.81 mmol) were added. The mixture was degassed and then refluxed under nitrogen for 3 days. The reaction mixture was cooled to rt, and then diluted with diethyl ether and filtered through Celite to remove the catalyst. The solvent was removed under vacuum to give 1.1 g of a dark oil. Crystallization from MeOH afforded 0.160 g of the target compound as a white solid. m.p. = 187–189 °C; ^1^H-NMR (CDCl_3_, 400 MHz): 3.91 (s, 3H, CH_3_), 3.94 (s, 3H, CH_3_), 4.00 (s, 3H, CH_3_), 6.90 (d, J = 8.9 Hz, 1H, Ar-H), 7.33 (dd, J = 2.8, 8.9 Hz, 1H, Ar-H), 7.62 (d, J = 8.9 Hz, 1H, Ar-H), 7.74 (d, J = 2.8 Hz, 1H, Ar-H), 7.89 (d, J = 8.9 Hz, 1H, Ar-H); ^13^C-NMR (CDCl_3_, 100 MHz): 55.7, 56.3, 61.5, 108.6, 111.0, 112.7, 116.7, 120.9, 123.0, 124.4, 128.6, 136.8, 144.6, 153.4, 159.3, 160.9.

3,4,8-Trihydroxy-6H-benzo[c]chromen-6-one (15)

The compound was synthesized according to the literature procedure [31]. To a solution of 3,4,8-trimethoxy-6H-benzo[c]chromen-6-one (r, 0.080 g, 0.28 mmol) in CH_2_Cl_2_ (10.0 mL), BBr_3_ in CH_2_Cl_2_ (1.0 M, 1.67 mmol) was added dropwise at 0 °C. The solution was stirred at rt for 12 h. Water (20.0 mL) was added to quench the reaction and the aqueous layer was extracted with EtOAc (3 × 20 mL). The organic phase was dried (Na_2_SO_4_), filtered, and concentrated in vacuo to give a white powder. The solid obtained was triturated in Et_2_O and filtered to afford 0.060 g (88%) of the target compound as yellow solid. m.p. = 341–343 °C; ^1^H-NMR (CD_3_OD, 400 MHz): 6.80 (d, J = 8.7 Hz, 1H, Ar-H), 7.28 (dd, J = 2.7, 8.7 Hz, 1H, Ar-H), 7.42 (d, J = 8.7 Hz, 1H, Ar-H), 7.59 (d, J = 2.7 Hz, 1H, Ar-H), 7.97 (d, J = 8.7 Hz, 1H, Ar-H); ^13^C-NMR (CD_3_OD, 100 MHz): 111.1, 111.9, 112.1, 113.4, 120.1, 123.2, 123.8, 128.1, 132.5, 138.8, 146.4, 157.0, 161.6; HRMS: found M-H: m/z 243.0307, calculated value for C_13_H_7_O_5_-: 243.0299 (delta: −3.3 ppm).

### 2.2. Kinase Assay

The assay was performed externally at BPS Bioscience (San Diego, CA, USA). Pyruvate kinase (PKL and PKR) reactions were conducted in triplicate at rt for 30 min in a 25 µL mixture containing 50 mM tris, pH 7.4, 10 mM MgCl_2_, 100 mM KCl, 0.05% Tween, 0.1 mM ADP, 0.125 mM PEP, pyruvate kinase (see Supplementary Materials) and the test compounds (see Supplementary Materials). The final DMSO concentration in the reaction was 1% v/v. After enzymatic reactions, 25 μL of Kinase-Glo Max reagent was added to each well and luminescence was measured using a BioTek Synergy^TM^ 2 microplate reader. The Kinase-Glo Max luminescence assay kit measures PK activity by quantitating the amount of ATP produced following a PK reaction. Enzyme activity assays were performed in triplicate at each concentration. The luminescence data were analyzed using GraphPad Prism. In the absence of the compound, the intensity (C_e_) in each data set was defined as 100% activity. In the absence of the enzyme, the intensity (C_0_) in each data set was defined as 0% activity. The percentage activity in the presence of each compound was calculated according to the following equation: % activity = (C-C_0_)/(C_e_-C_0_), where C is the luminescence in the presence of the compound.

### 2.3. Molecular Modelling

#### 2.3.1. Homology Modelling

A homology model of inactive PKL was constructed for induced-fit docking (IFD) using the reported crystal structure of PKM2. PKM2 has been crystallized in both an active R-state and an inactive T-state, while no crystal structure of PKL in the inactive state is available [32,33,34]. For this reason and due to the high degree of homology, the 3D structure of inactive PKM2 (PDBID: 6GG4) was used as the template structure for the homology modelling of the inactive PKL tetramer [32]. The PKL protein sequence was retrieved from Uniprot (entry: P30613). The homology model of inactive PKL was built using the StructurePrediction panel in Schrödinger Suite (Schrödinger, LLC, New York, NY, USA) [35]. The ClustalW method was used to align the target and template sequences in Prime. The energy-based method was selected for model building, and homo-multimer was selected as the multi-template model type.

#### 2.3.2. Induced Fit Docking

IFD was performed to identify the potential interactions of the compounds with the protein [36]. All compounds were prepared using the LigPrep module, and the protein was prepared using the Protein Preparation Wizard in Schrödinger. The compounds were docked to the rigid protein using Glide with a protein van der Waals radii scaling of 0.5, and ligand van der Waals scaling of 0.5. The resulting top 20 docking poses were used to sample the binding pocket plasticity. Residues within 5 Å of the 20 ligand poses were subject to side chain optimization, with the remainder of the residues held fixed. Therefore, the flexibility of the protein was considered for the forward redocking stage. The parameters for the redocking were all set to default.

#### 2.3.3. Prime-MMGBSA Energy Calculations

The binding free energy of the protein–ligand complexes was calculated by the Prime MM-GBSA method. The best IFD docking poses of each protein–ligand complex were used for this calculation. The following equation was used to determine the binding free energies Δ𝐺_bind_:

Δ𝐺_bind_ = 𝐺_complex_ − (𝐺_protein_ + 𝐺_ligand_) (1)

where 𝐺 = 𝐸_MM_ + 𝐺_SGB_ + 𝐺_NP_. 𝐸_MM_ is the molecular mechanism energies, 𝐺_SGB_ is SGB solvation energy for polar solvation, and 𝐺_NP_ is a nonpolar solvation energy.

## 3. Results

### 3.1. Chemistry

Compounds **1**, **2**, **3**, **5**, **7**, **11**, and **12** were commercially available and were purchased from the corresponding vendor. Some of the target compounds (**4**, **8**, **9**, **10**, and **15**) were reported previously in the chemical literature; their synthesis was replicated here, and the molecules were isolated and characterized accordingly [24,29,30,31]. The other compounds **6**, **13**, and **14** were synthesized in-house.

### 3.2. Initial Screening Results

**Figure 1:**
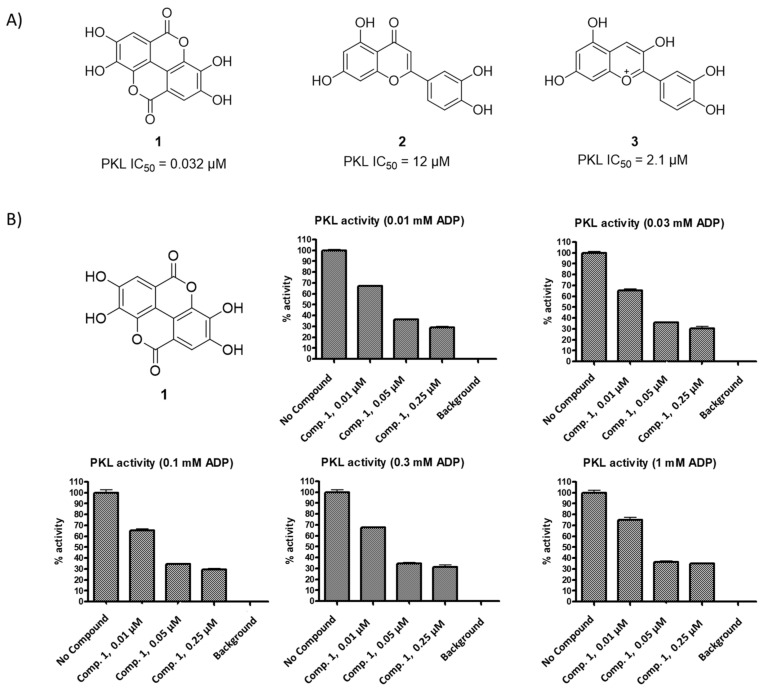
(**A**) Polyphenols exhibiting PKL inhibition. Ellagic acid (EA, **1**), luteolin (**2**), cyanidin (**3**). (**B**) Inhibition of PKL by compound **1** at different concentrations of the substrate ADP.

Initial screening was performed on a selected library of polyphenols, which identified three compounds possessing an inhibitory effect on PKL (Figure 1A). EA (**1**) was the most effective with an IC_50_ of 0.032 µM. Table 1 reports the IC_50_ values for all compounds.

Compound **1** was then evaluated against the other PK isoforms, showing a very good selectivity profile (Table 1). This behavior implied non-competitive inhibition, and in order to test this, we evaluated the activity of compound **1** in the presence of different concentrations of ADP (Figure 1B). The observed activity was independent of substrate concentration, confirming that **1** is a non-competitive allosteric inhibitor. EA possesses exceptional pharmacological properties against liver toxicity and disease, and it has been successfully employed in the past as a lead compound for the development of a new class of CK2 inhibitors [37,38].

### 3.3. Deconstruction of EA and Its Metabolites

**Figure 2:**
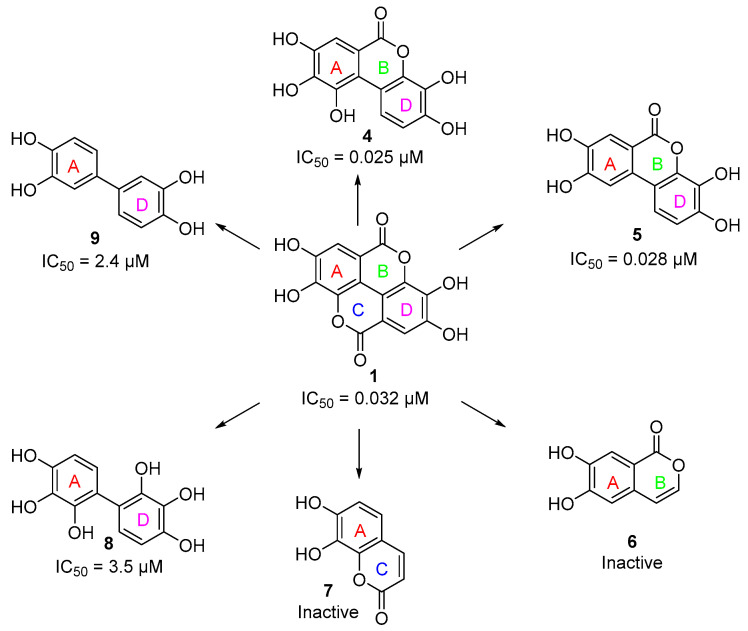
Deconstruction of EA and corresponding IC_50_ *vs.* PKL.

EA is a reasonable starting point for the development of potent and selective PKL inhibitors. EA has a complex structure, is symmetrical in nature, and has unfavorable physicochemical properties. Therefore, rather than investigating simple modifications, we immediately applied the “deconstruction” approach to EA (Figure 2). This strategy involves simplifying the structure step-by-step to identify the elements of the molecule required for PKL activity. We decided to focus our study on the number of rings in the molecule. Firstly, one carbonyl group was omitted, partially removing the C-ring, which resulted in urolithin M5 (Uro M5, **4**), a well-known metabolite of EA. Compound **4** showed a similar activity and selectivity to the parent compound (Table 1). Further simplification of **1** yielded another metabolite, urolithin D (Uro D, **5**). Again, the compound retained the activity and most of the selectivity of the lead molecule (Table 1). Removal of the C- and D-rings produced the inactive isochromenone **6**.

**Figure 3:**
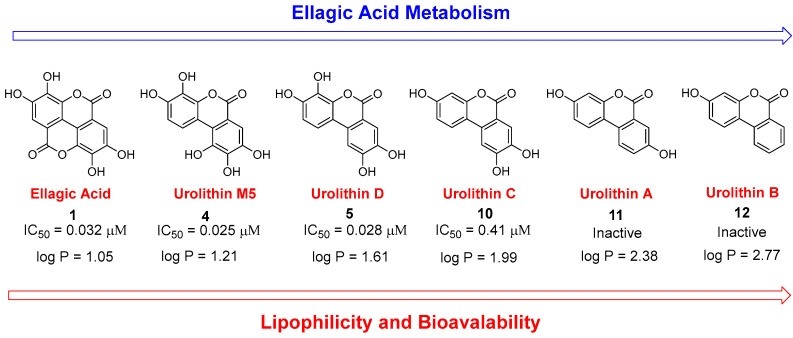
EA metabolites and corresponding IC_50_ *vs.* PKL.

In terms of the structure–activity relationship, the common features of all active ligands tested in this study were the two aromatic rings and the hydroxyl groups. The lactone moiety seems to play an important but not crucial role. Since the two most active compounds, **4** and **5**, are generated in vivo from EA metabolism, we decided to test all its possible metabolites. It is well known that EA is gradually metabolized in the intestine to produce urolithin (Uro) M5, Uro D, Uro C, and finally Uro A and Uro B (Figure 3) [38]. All these compounds share the same three-ring system, but they have a different hydroxyl substitution pattern. The removal of a hydroxyl group from **5** produces Uro C (**10**), which retains some level of activity that is nonetheless reduced by approximately one order of magnitude. Deletion of an additional hydroxyl group, to give Uro A (**11**), proved to be detrimental for the activity since no inhibition was observed, and similarly for the monohydroxyl compound Uro B (**12**).

**Figure 4:**
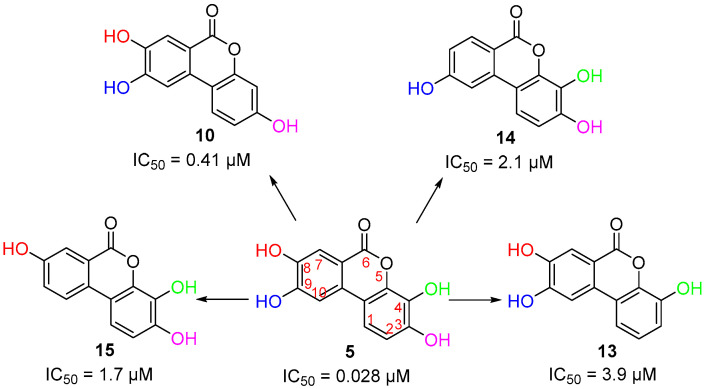
Urolithin C positional isomers and corresponding IC_50_ vs. PKL.

These data suggest that the hydroxyl groups at position 4 and especially those at position 9 play a critical role in the binding. However, we decided to further investigate which of the hydroxyl groups of Uro D are required for binding (Figure 4). The deletion of the 3-hydroxyl moiety to give **13** caused a hundred-fold reduction in activity. Removal of the 8-hydroxyl group of **5** gave 8-des-OH Uro D (**14**), and removal of the 9-hydroxyl group gave 9-des-OH Uro D (**15**). Both agents had dramatically reduced inhibitory activity. Evidently, all the hydroxyl groups of Uro D modulate the potency, but the very presence of hydroxyl groups per se is not a requirement for the desired activity.

### 3.4. Molecular Modelling Studies

**Figure 5:**
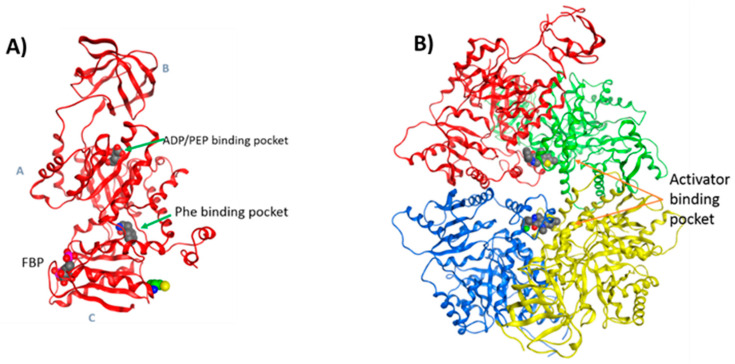
(**A**) Monomeric structure of PKL with different binding sites illustrated. (**B**) PKM2 active tetramer structure with activator binding (PDB ID: 3U2Z) [36].

To further understand the binding mode, binding site, and binding energy between EA derivatives and PKL, molecular modelling studies were performed. Initially, we attempted to rationalize our data by docking our active molecules into the PKL crystal structure (PDB ID: 3U2Z). Induced-fit docking was performed to search for potential interactions of the compounds with PKL. This setup takes into account the protein flexibility, which provides a higher degree of accuracy. As shown in Figure 5A, four possible binding pockets have been previously reported in the PKL structure for small molecules: FBP, PEP, ADP, and Phe binding pockets [39]. As such, we speculated that these new inhibitors might bind to one of these binding pockets. However, the ADP binding site was immediately disqualified from the docking study, since we demonstrated unequivocally that **1** is a non-competitive inhibitor.

**Figure 6:**
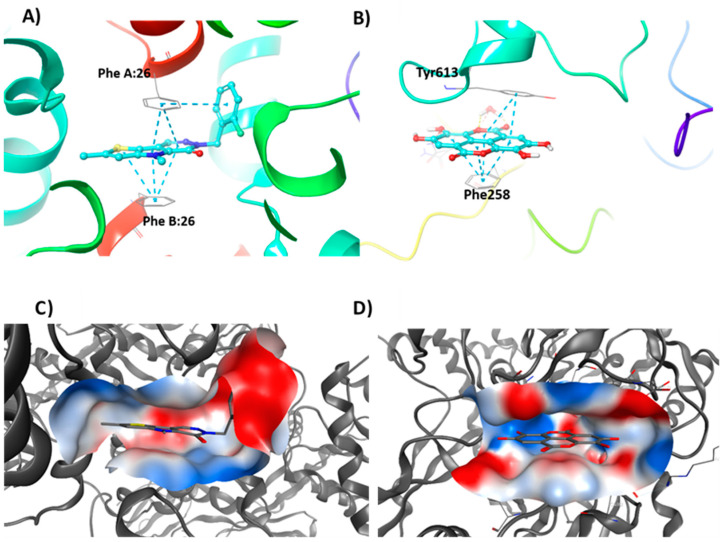
(**A**) Crystal structure of PKM2 activator in its binding site, PDB ID: 3H6O. (**B**) Crystal structure of compound 1 and glycogen phosphorylase, PDB ID: 4YUA. (**C**) Electrostatic map of the PKL binding pocket. The backbone is illustrated as a grey ribbon. (**D**) Electrostatic map of the glycogen phosphorylase binding pocket. The backbone is illustrated as a grey ribbon.

Moreover, a detailed analysis of the crystal structure and the literature revealed an additional possible binding site. Compound **1** behaves as a weak activator for PKM2, and has a very planar structure. Recently, a class of PKM2 activators with the same planarity as compound **1** were reported to bind to the inter-monomer interface of the active PKM2 tetramer (Figure 5B and Figure 6A). These PKM2 activators bind between two phenylalanine residues and form π–π interactions at the binding interface, thereby stabilizing the active tetramer (Figure 6A) [40]. For this reason, we hypothesize that EA and its derivatives might bind in the same fashion to the PKL dimer interface, resulting in inhibition rather than activation. Furthermore, compound **1** has been previously crystallized with two proteins, glycogen phosphorylase [41] and human CK2 alpha [42]. When analyzing the binding pockets of these two proteins, we found that **1** has a very similar binding pose in both crystal structures, since it lies between a phenylalanine and a tyrosine residue forming π–π interactions (Figure 6B).

As these binding modes are remarkably close to what was observed in PKM2, we also included this site in the docking study. Therefore, the FBP, PEP, and Phe pockets and the pocket at the binding interface of inactive PKL were selected for IFD to investigate the potential binding interactions of compound **1**. EA was docked into the different binding pockets, and the prime MMGBSA method was used to calculate the relative binding energies (∆G_bind_) of the complex. More negative values of ∆G_bind_ indicate stronger binding. The data obtained (Table 2) showed that compound **1** has a stronger interaction with the binding site located at the tetramer interface of PKL, suggesting that the observed inhibition might be related to the stabilization of the inactive PKL state.

**Figure 7:**
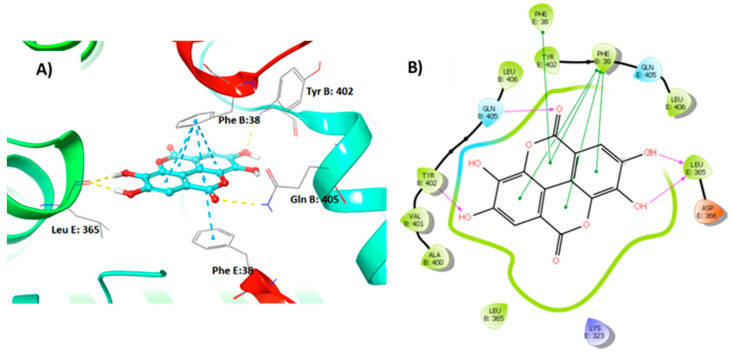
Compound **1** interactions with PKL tetramer. (**A**) 3D structure of the binding pose. (**B**) 2D structure of the binding pose.

The best binding pose of compound 1 in the inactive tetramer interface is shown in Figure 7, and involves two phenylalanine residues from Chain B and E; π–π interactions therefore play an important role.

Furthermore, two hydroxyl groups form hydrogen bonds to Leu365 of Chain E on the other side of the compound. The hydroxyl group acts as a hydrogen bond acceptor that interacts with the backbone of Tyr402 of Chain B, and the carbonyl forms a hydrogen bond with Gln405 of Chain B. These data are confirmed by the structure–activity relationship studies performed on the EA derivatives. Moreover, this binding mode could explain not only the non-competitive inhibition shown by **1,** but also the good selectivity observed versus PKR, notwithstanding the high homology sequence.

## 4. Discussion

**Figure 8:**
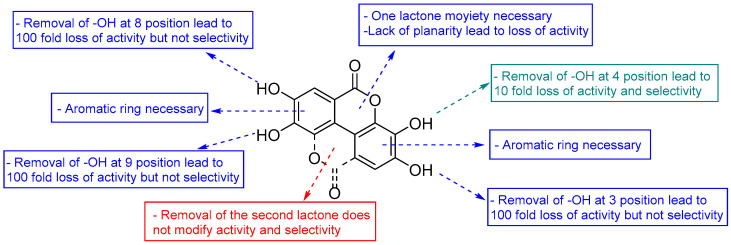
SAR study on EA and its derivatives as PKL inhibitors.

EA is a polyphenol commonly found in various fruits [43], and it is metabolized in vivo into urolithins, which may mediate its pharmacological effects [44,45,46]. Several studies have revealed that EA possesses potent biochemical and biological activities, including antioxidative, anti-inflammatory, and neuroprotective effects. EA substantially decreased de novo lipogenesis in adipocytes [47] and hepatocytes [48], and could activate the AMP-activated protein kinase (AMPK) [49]. Urolithins prevented triglyceride synthesis and accumulation, and concomitant downregulation of suppressing fatty acid synthase (FAS) by activating AMPK [50]. Furthermore, EA has also been shown to ameliorate hepatic steatosis by activating AMPK [51]. In this study, EA and its metabolites were identified as potent inhibitors of PKL. As previously reported, PKL upregulation is strongly associated with NAFLD severity [8,9]. Our data suggest that the effect of EA and its metabolites on hepatic steatosis, at least in part, is mediated through inhibition of PKL. Moreover, the structure–activity relationship of these compounds (Figure 8) was investigated using in vitro inhibition assays and molecular docking. The minimum requirements to retain nanomolar activity were (a) 4 hydroxyl groups at positions 3, 4, 8 and 9 on the urolithin core, (b) one lactone moiety (ring b), and (c) the planarity of the structure. Molecular docking provided valuable insight into the binding interactions of EA and its derivatives. This study provides a new class of non-competitive inhibitors of PKL. We speculate that Uro-D and Uro-C, in particular, could be used as starting points to develop new PKL inhibitors with better drug-like properties. These compounds could be useful in preventing or treating NAFLD. Studies are ongoing to evaluate their effect *in vivo*, and will be published in due course.

## Figures and Tables

**Table 1:** In vitro Pyruvate Kinase assay.

Compound	PKL IC50	PKR IC50	PKM1 IC50	PKM2 IC50
**1**	0.032 µM	4.7 µM	81 µM	0.07 µM ^a^
**2**	12 µM	>100 µM	N.D.	N.D.
**3**	2.1 µM	7.4 µM	N.D.	N.D.
**4**	0.025 µM	0.49 µM	8 µM	>100 µM
**5**	0.028 µM	1.27 µM	36 µM	0.03 µM ^a^
**6**	Inactive ^b^	Inactive ^b^	N.D.	N.D.
**7**	Inactive ^b^	Inactive ^b^	N.D.	N.D.
**8**	3.5 µM	12 µM	N.D.	N.D.
**9**	2.4 µM	5.1 µM	N.D.	N.D.
**10**	0.41 µM	2.6 µM	N.D.	N.D.
**11**	Inactive ^b^	Inactive ^b^	N.D.	N.D.
**12**	Inactive ^b^	Inactive ^b^	N.D.	N.D.
**13**	3.9 µM	43 µM	N.D.	N.D.
**14**	2.1 µM	68 µM	N.D.	N.D.
**15**	1.7 µM	>100 µM	N.D.	N.D.
FBP *	0.02 µM ^a^	0.01 µM ^a^	Inactive	0.05 µM ^a^

^a^ The compound behaves as an activator. The value refers to EC_50_; ^b^ compounds with less than 30% inhibition at 10 µM were considered inactive. *FBP = Fructose-1,6-biphosphate.

**Table 2:** The binding energies of compound **1** in different binding sites.

Binding Site	∆G_bind_ (kcal/mol) ^a^
FBP	−66.56
PEP	−39.51
Phe	−72.11
Inactive tetramer interface	−75.36

^a^ ∆G_bind_ = E_complex_ *−* (E_protein_ + E_ligand_).

## Data Availability

Data are available in the Supporting Materials.

## Supplementary Materials

The following supporting information can be downloaded at: https://www.mdpi.com/article/10.3390/nu15030577/s1. Figures of 1H- and ^13^C-NMR spectra of the synthesized compounds.
