# Supplementary material for: Ellagic Acid and Its Metabolites as Potent and Selective Allosteric Inhibitors of Liver Pyruvate Kinase
Source: Nutrients. 2023 Jan 22;15(3):577. doi: 10.3390/nu15030577 (PMC9919951; doi:10.3390/nu15030577)
Supplement: Supplementary file 1 [file nutrients-15-00577-s001.zip › nutrients-2130176-supplementary.pdf]

Ellagic Acid and its metabolites as potent and selective allosteric inhibitors of Liver Pyruvate Kinase

Umberto Maria Battisti [1,2,†], Chunixa Gao [1,2,†], Fady Akladios [1,2], Woonghee Kim [2], Hong Yang [2], Cemil Bayram [3], Ismail Bolat [4], Metin Kiliclioglu [4], Nursena Yuksel [5], Ozlem Ozdemir Tozlu [5], Cheng Zhang [2,6], Jihad Sebhaoui [7], Shazia Iqbal [7], Saeed Shoaie [2,8], Ahmet Hacimuftuoglu [3], Serkan Yildirim [4], Hasan Turkez [9], Mathias Uhlen [2], Jan Boren [10,11], Adil Mardinoglu [2,8] and Morten Grøtli [1,*]

[1] Department of Chemistry and Molecular Biology, University of Gothenburg, 41296 Gothenburg, Sweden
[2] Science for Life Laboratory, KTH–Royal Institute of Technology, 10450, Stockholm, Sweden
[3] Department of Medical Pharmacology, Faculty of Medicine, Atatürk University, 25240 Erzurum, Turkey
[4] Department of Pathology, Faculty of Veterinary, Atatürk University, 25240 Erzurum, Turkey
[5] Department of Molecular Biology and Genetics, Faculty of Science, Erzurum Technical University, 25050 Erzurum, Turkey
[6] School of Pharmaceutical Sciences, Zhengzhou University, Zhengzhou 450001, China
[7] Trustlife Labs, Drug Research & Development Center, Heka Human Drug Research & Development Center, 34774 Istanbul, Turkey
[8] Centre for Host-Microbiome Interactions, Faculty of Dentistry, Oral & Craniofacial Sciences, King's College London, London SE1 9RT, UK
[9] Department of Medical Biology, Faculty of Medicine, Atatürk University, 25240 Erzurum, Turkey
[10] Department of Molecular and Clinical Medicine, University of Gothenburg, 40530 Gothenburg, Sweden
[11] Sahlgrenska University Hospital, 40530 Gothenburg, Sweden
* Correspondence: grotli@chem.gu.se; Tel.: +46-766229017
† These authors contributed equally to this work.

General information

Unless otherwise noted, commercially available chemicals, reagents and solvents were used without any purification. Microwave reactions were performed in capped vials using a Biotage Initiator Sixty instrument with fixed hold time. The reactions were monitored by thin-layer chromatography (TLC) on silica plated aluminum sheets (Silica gel 60 F254, E. Merck) and detecting spots by UV light. Flash chromatography was performed on silica gel 60 (0.040−0.063 mm, manually or using a Biotage SP4 Flash instrument). ^{1}H NMR (400 MHz) and ^{13}C NMR (100 MHz) spectra were recorded on a JEOL JNM-ECP 400 instrument, using $CDCl_3$, CD_3OD or DMSO-d_6 as solvent as indicated. Chemical shifts are reported in ppm with the solvent residual peak as internal standard: ^{1}H – residual $CHCl_3$ (δ_H 7.26), CD_3OD (δ_H 3.31) or DMSO-d_6 (δ_H 2.50) and ^{13}C – $CDCl_3$ (δ_C 77.16), CD_3OD (δ_C 49.80) or DMSO-d_6 (δ_C 39.52). NMR data are reported as follows: chemical shift, number of protons/carbons, multiplicity (s, singlet; d, doublet; t, triplet; q, quartet; m, multiplet; br, broadened), coupling constants (Hz). Melting points were recorded on a Büchi melting point apparatus B-545 or a Mettler FP82 hot stage equipped with a FP80 temperature controller and are uncorrected. Accurate mass analyses were done by using a Agilent 6520 quadrupole time of flight instrument coupled to Agilent 1290 infinity ultra-performance liquid chromatograph (Santa Clara, USA). Samples were dissolved in acetonitrile and eluted by using isocratic elution (100% acetonitrile) with a flow rate of 0.4 ml/min. Mass spectrometer was operated in positive electrospray ionization scanning between 50 and 1200 m/z. Ion source parameters were as follows; drying gas flow 10 L/min and temperature 325°C and nebulizer pressure 35 psig. Mass spectrometer was calibrated before analyses. Compounds **1**-**3**, **7** were purchased from Sigma-Aldrich, while compounds **5**, **11**, **12** were purchased from Toronto Research Chemicals (20 Martin Ross Avenue, North York, M3J 2K8 Ontario, Canada).

NMR Spectra

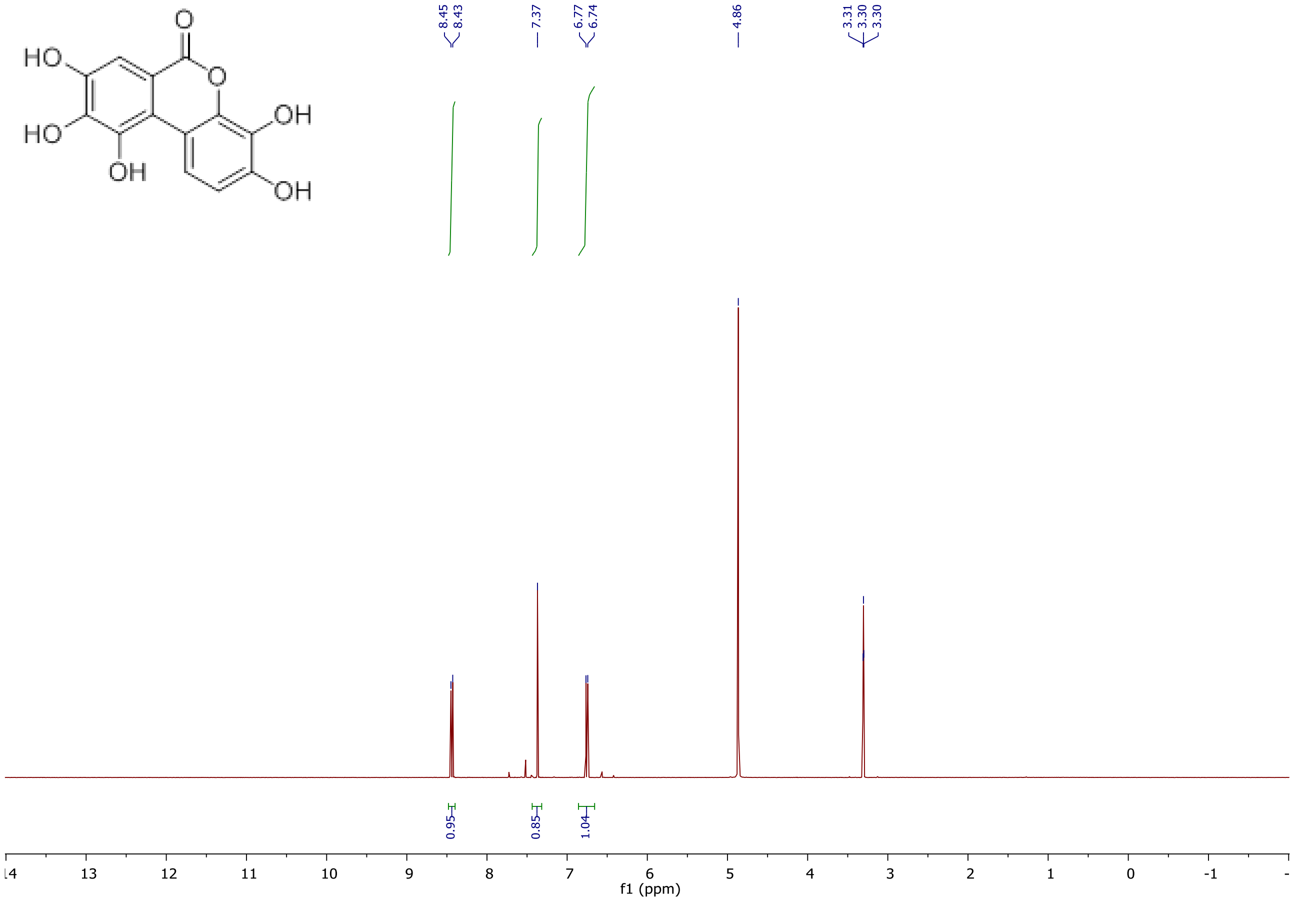

8.45
8.43
7.37
6.77
6.74
4.86
3.31
3.30
3.30
0.95
0.85
1.04
14 13 12 11 10 9 8 7 6 5 4 3 2 1 0 -1
f1 (ppm)

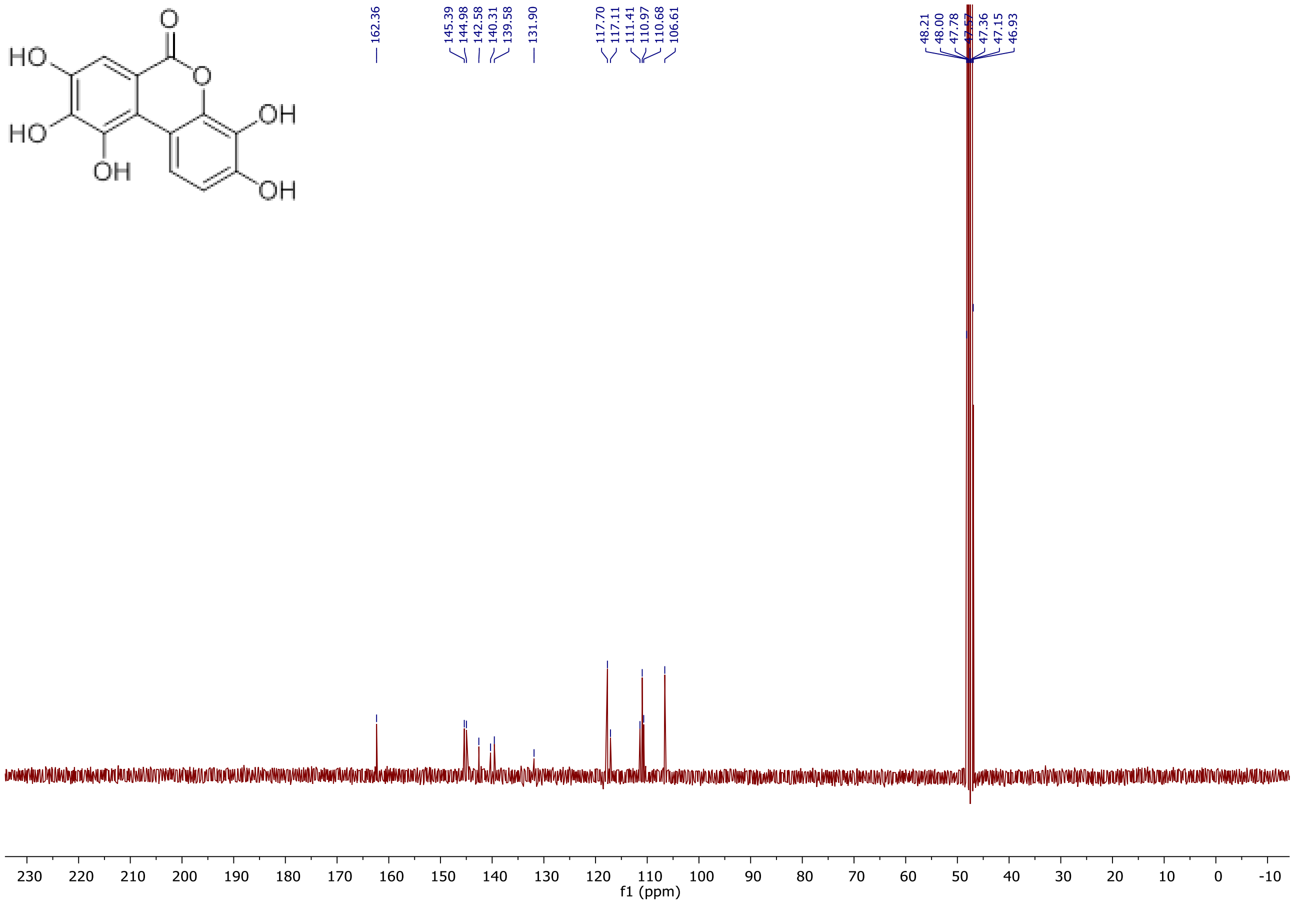

162.36
145.39
144.98
142.58
140.31
139.58
131.90
117.70
117.11
111.41
110.97
110.68
106.61
48.21
48.00
47.78
47.57
47.36
47.15
46.93
230 220 210 200 190 180 170 160 150 140 130 120 110 100 90 80 70 60 50 40 30 20 10 0 -10
f1 (ppm)

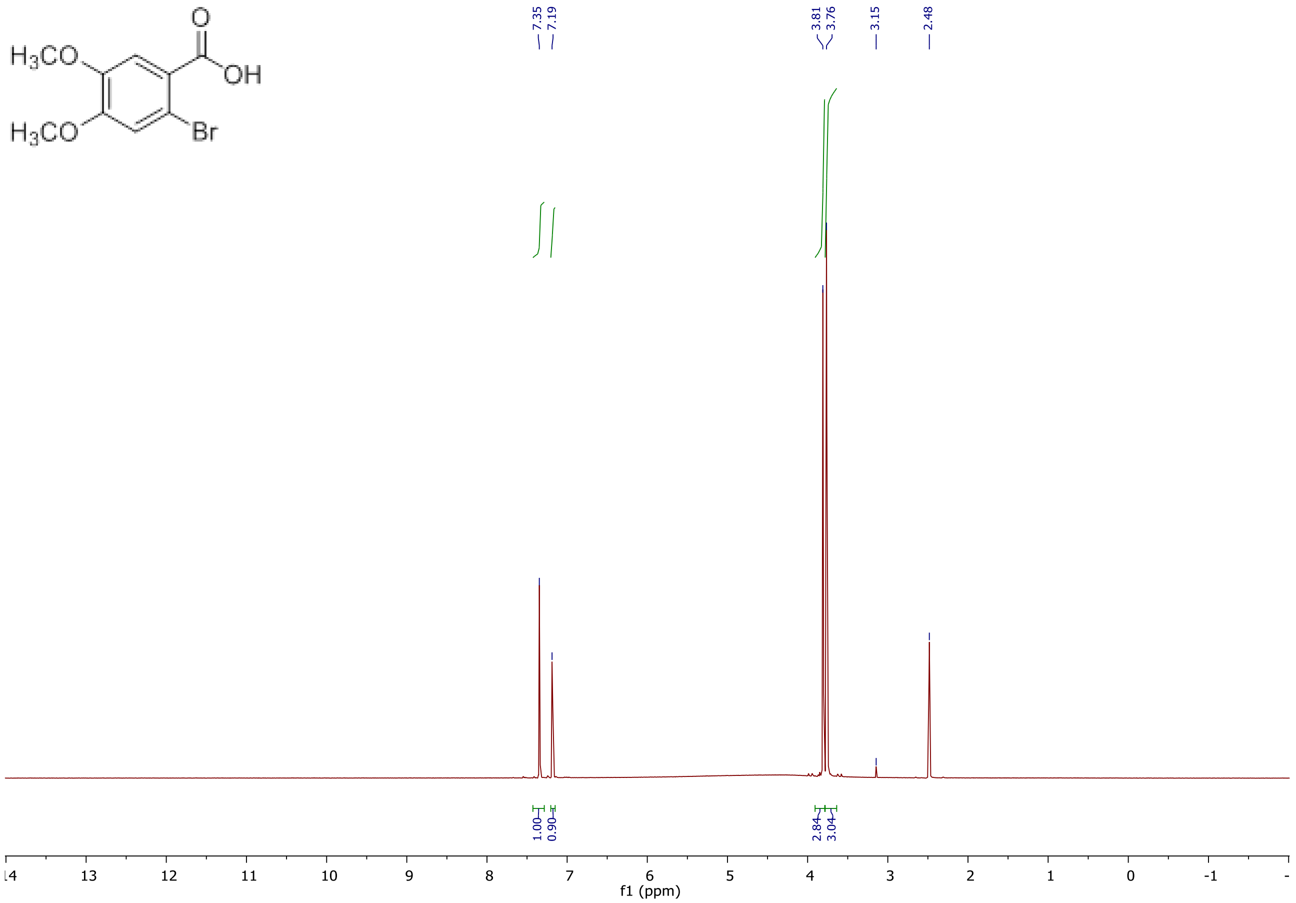
H3CO
H3CO
O
OH
Br
7.35
7.19
3.81
3.76
3.15
2.48
1.00
0.90
2.84
3.04
f1 (ppm)

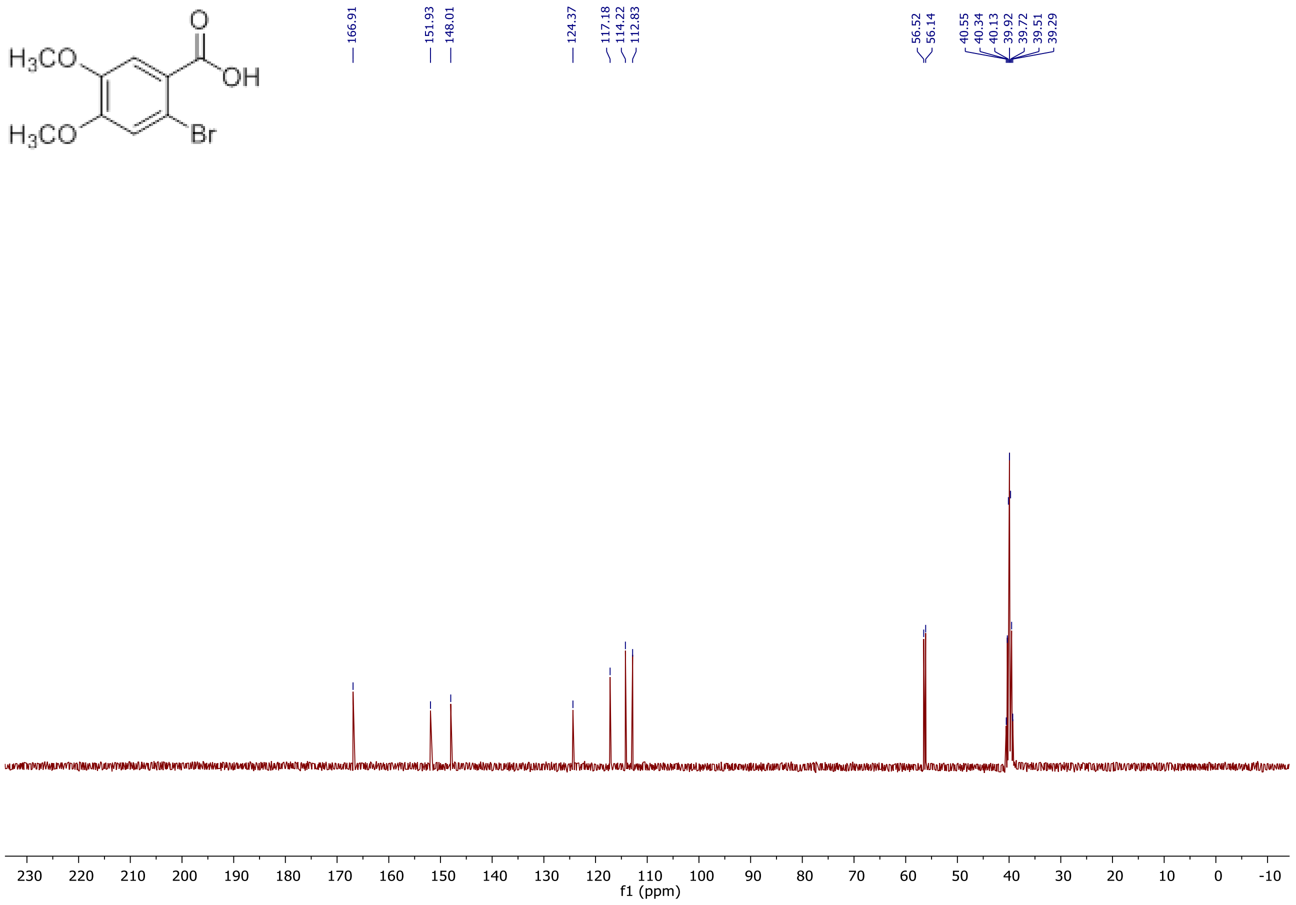
H3CO
H3CO
O
OH
Br
166.91
151.93
148.01
124.37
117.18
114.22
112.83
56.52
56.14
40.55
40.34
40.13
39.92
39.72
39.51
39.29
f1 (ppm)

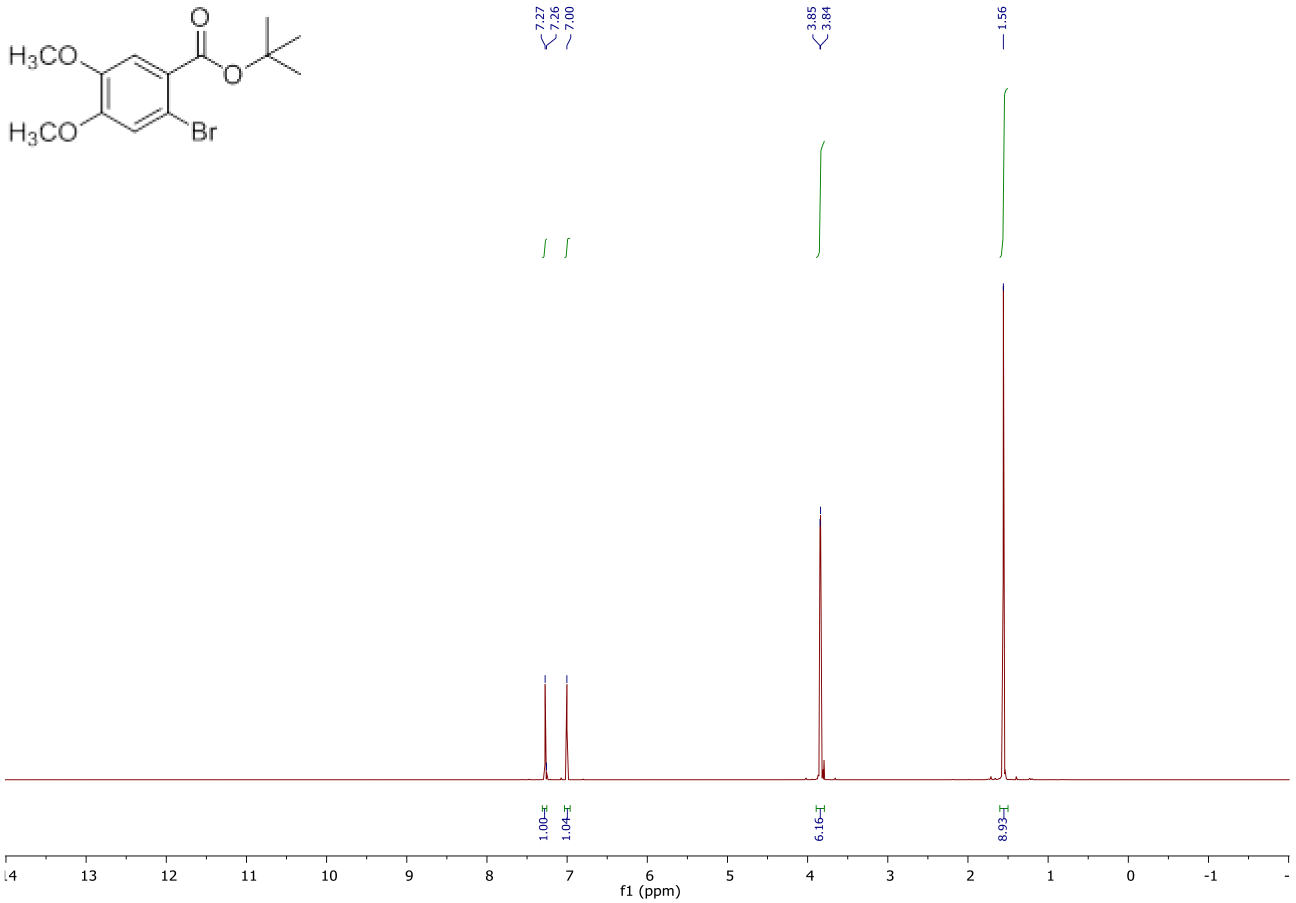
H3CO
H3CO
O
O
Br
7.27
7.26
7.00
3.85
3.84
1.56
1.00
1.04
6.16
8.93
f1 (ppm)

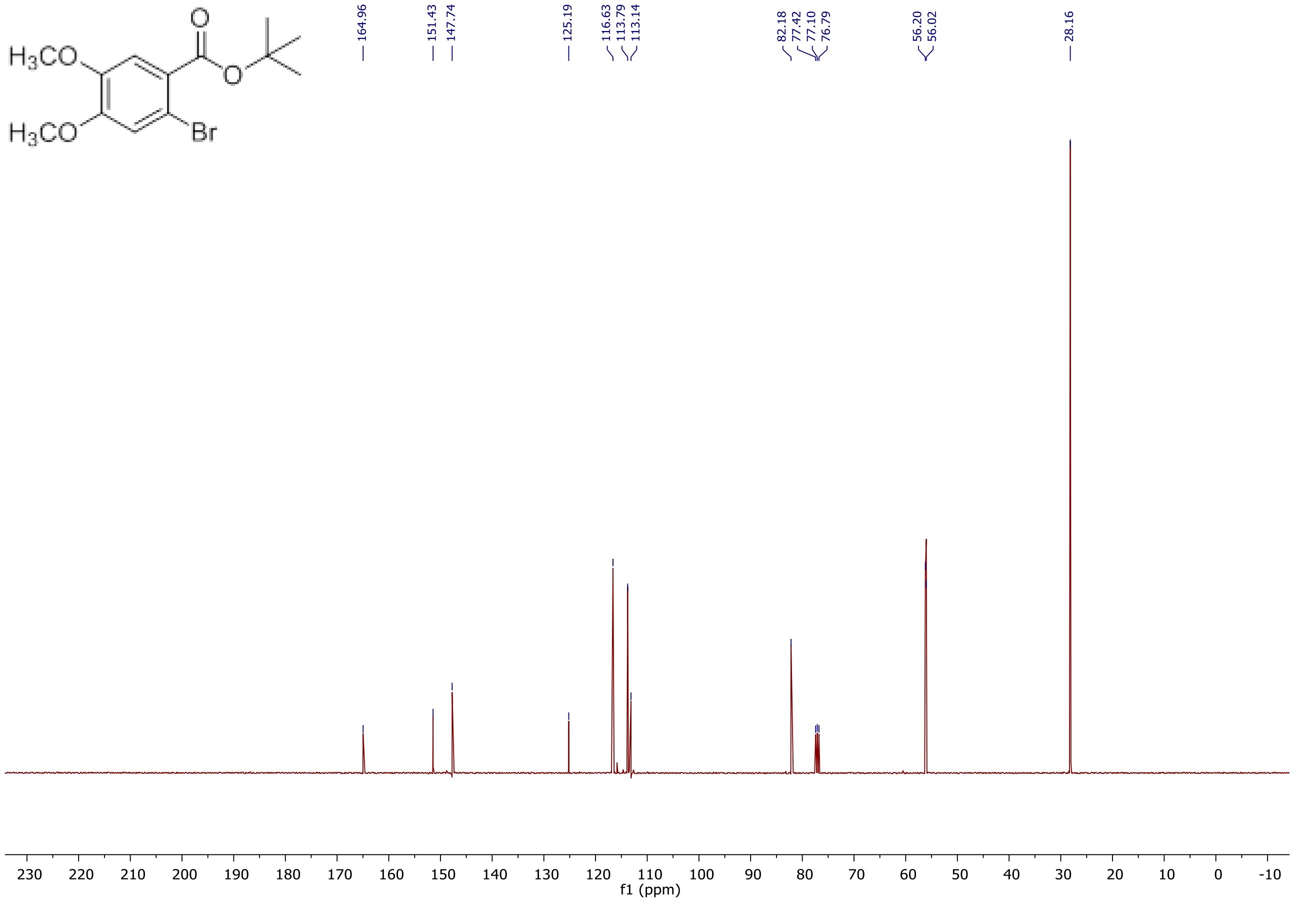
H3CO
H3CO
O
O
Br
164.96
151.43
147.74
125.19
116.63
113.79
113.14
82.18
77.42
77.10
76.79
56.20
56.02
28.16
f1 (ppm)

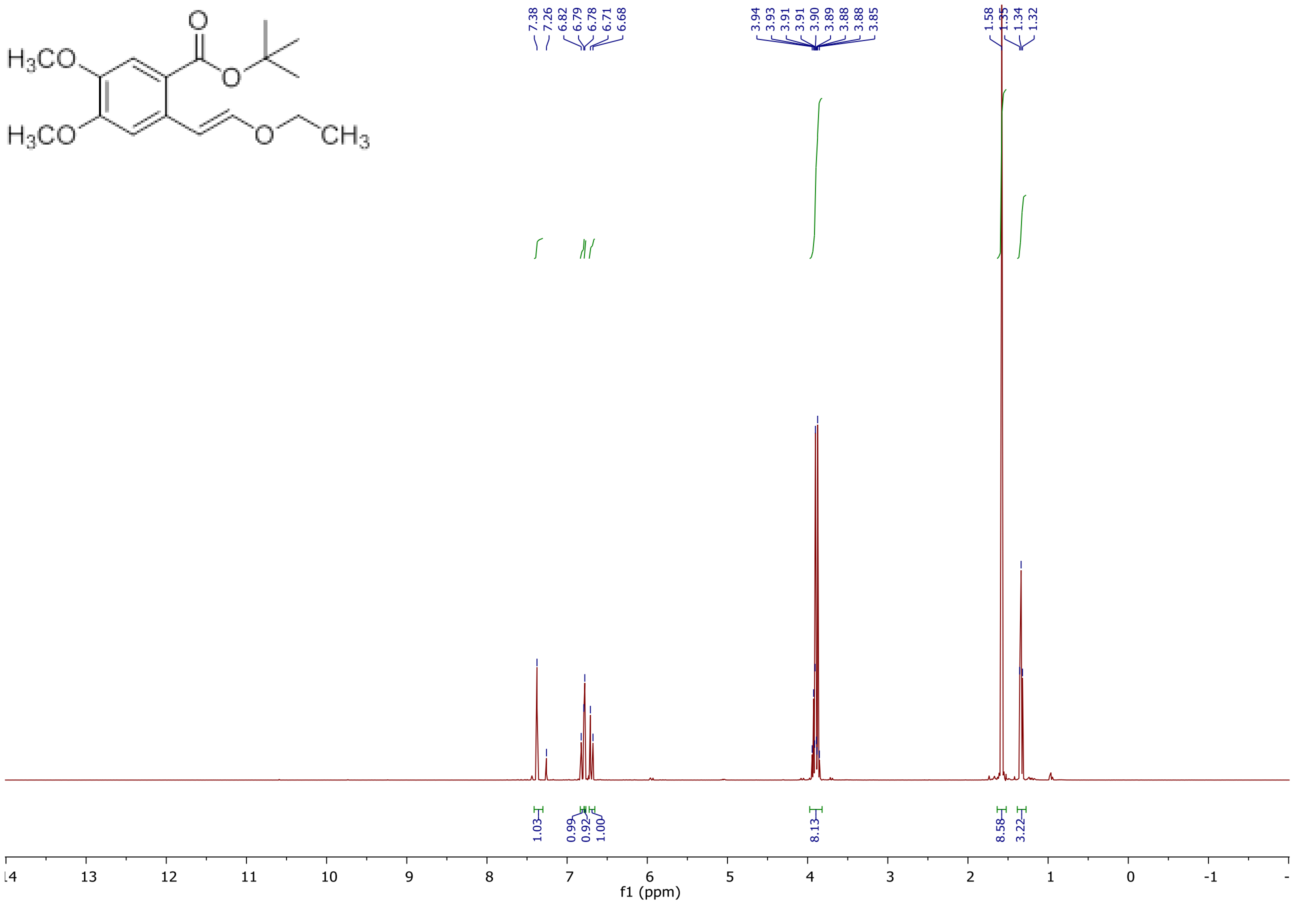
H3CO
H3CO
CH3
7.38 7.26 6.82 6.79 6.78 6.71 6.68
3.94 3.93 3.91 3.91 3.90 3.89 3.88 3.88 3.85
1.58 1.35 1.34 1.32
1.03 0.99 0.92 1.00
8.13
8.58 3.22
f1 (ppm)

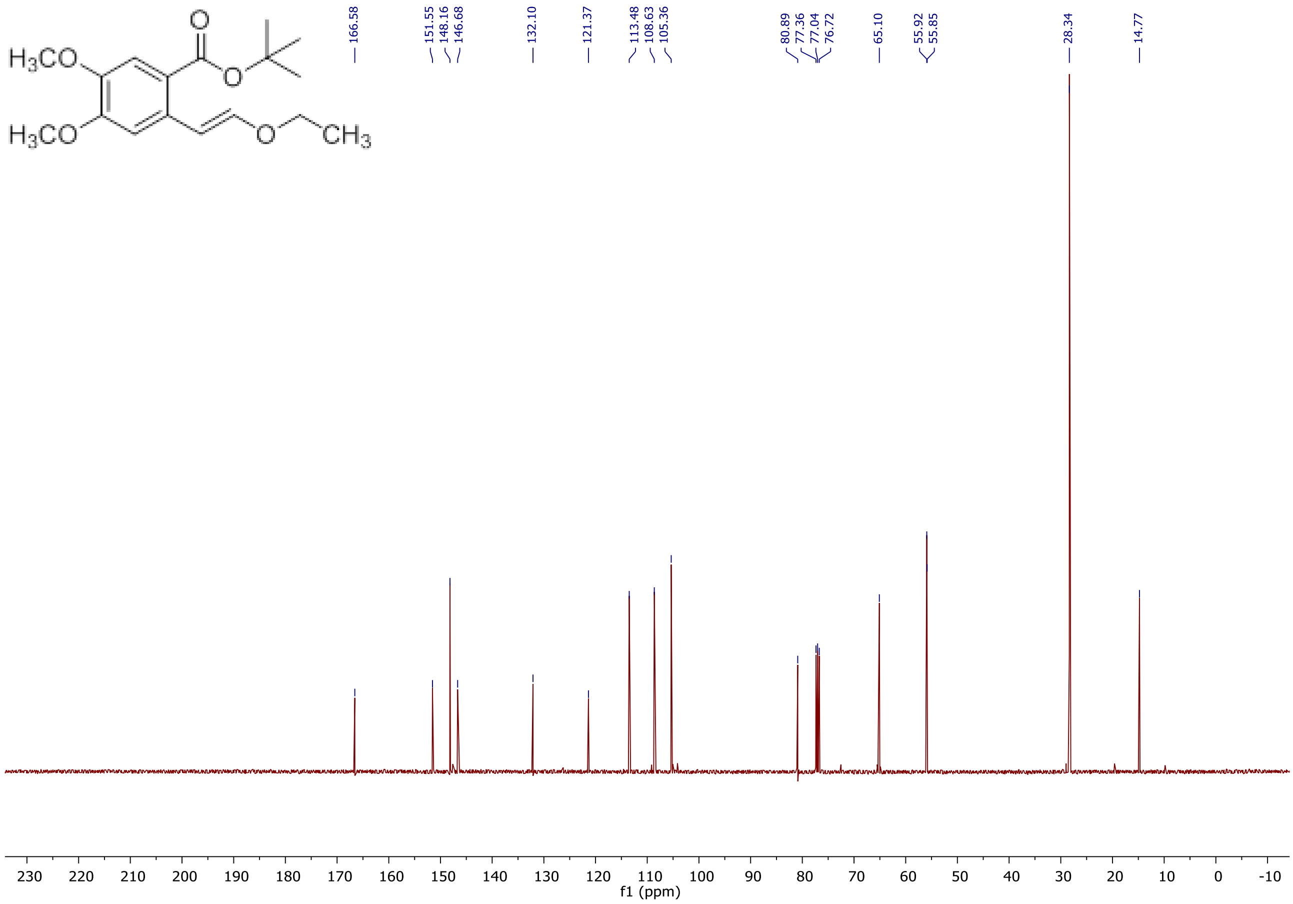
H3CO
H3CO
CH3
166.58
151.55 148.16 146.68
132.10
121.37
113.48 108.63 105.36
80.89 77.36 77.04 76.72
65.10
55.92 55.85
28.34
14.77
f1 (ppm)

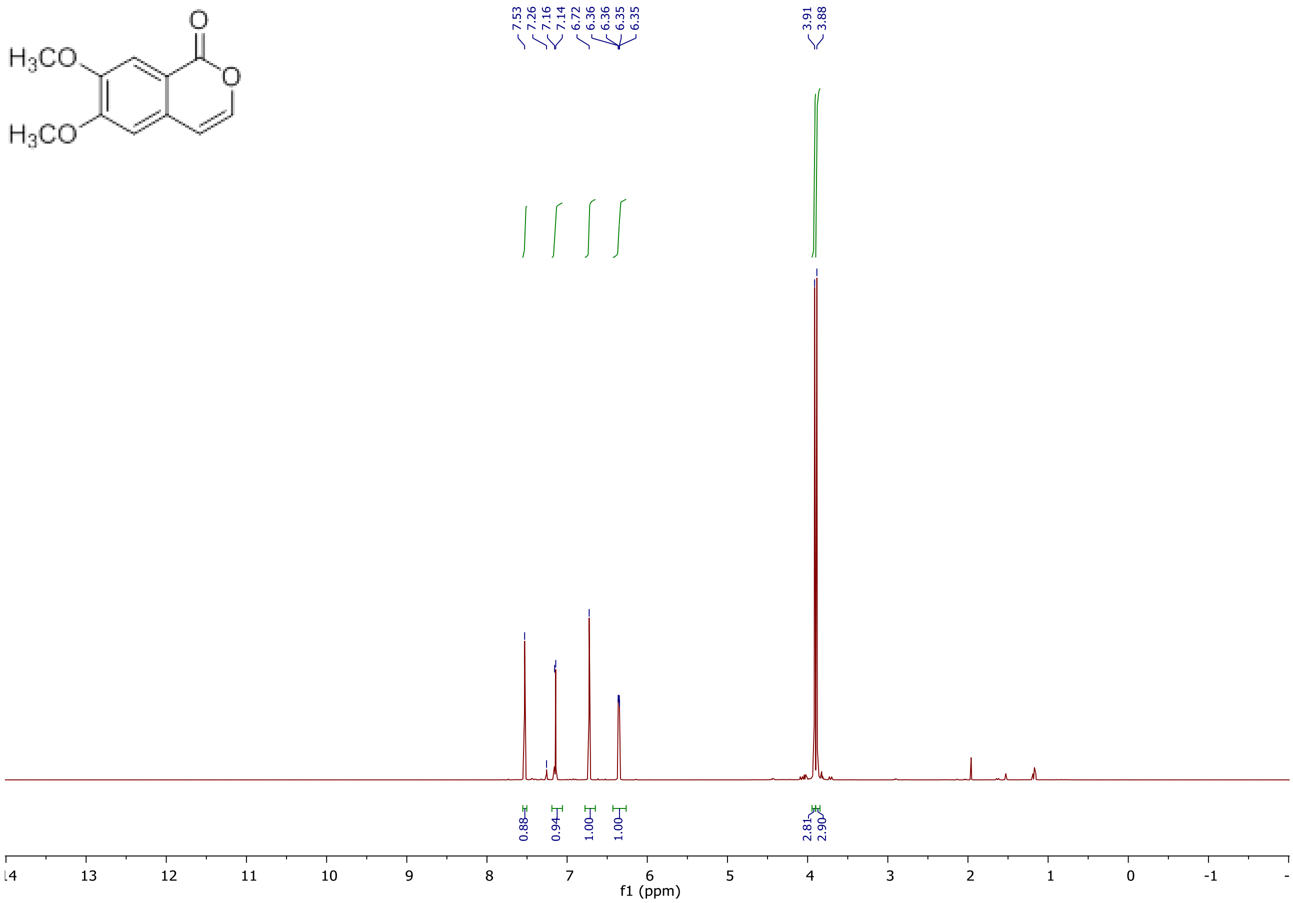
H3CO
H3CO
O
O
7.53
7.26
7.16
7.14
6.72
6.36
6.36
6.35
6.35
3.91
3.88
0.88
0.94
1.00
1.00
2.81
2.90
f1 (ppm)

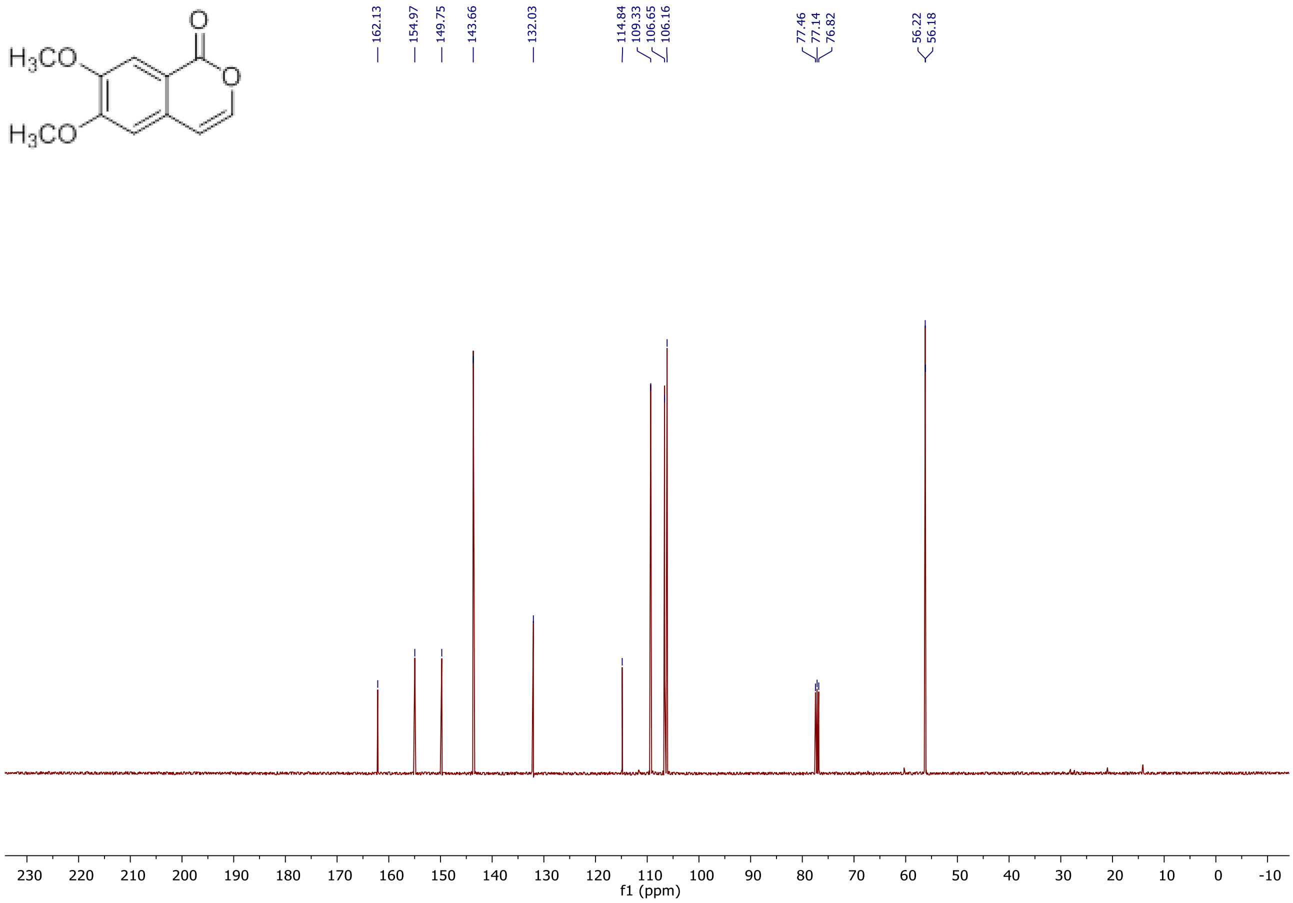
H3CO
H3CO
O
O
162.13
154.97
149.75
143.66
132.03
114.84
109.33
106.65
106.16
77.46
77.14
76.82
56.22
56.18
f1 (ppm)

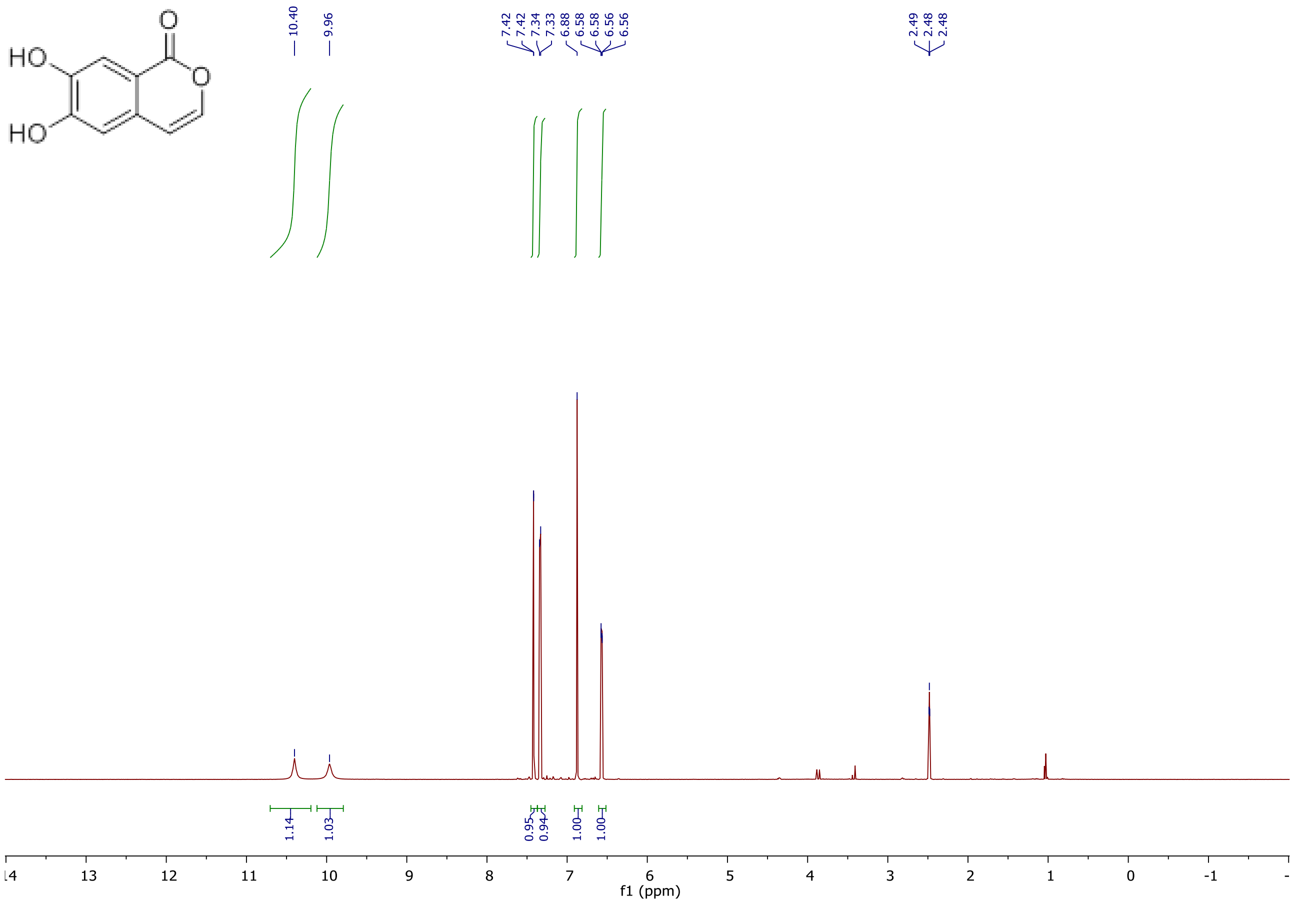
HO
HO
O
O
10.40
9.96
7.42
7.42
7.34
7.33
6.88
6.58
6.58
6.56
6.56
2.49
2.48
2.48
1.14
1.03
0.95
0.94
1.00
1.00
f1 (ppm)

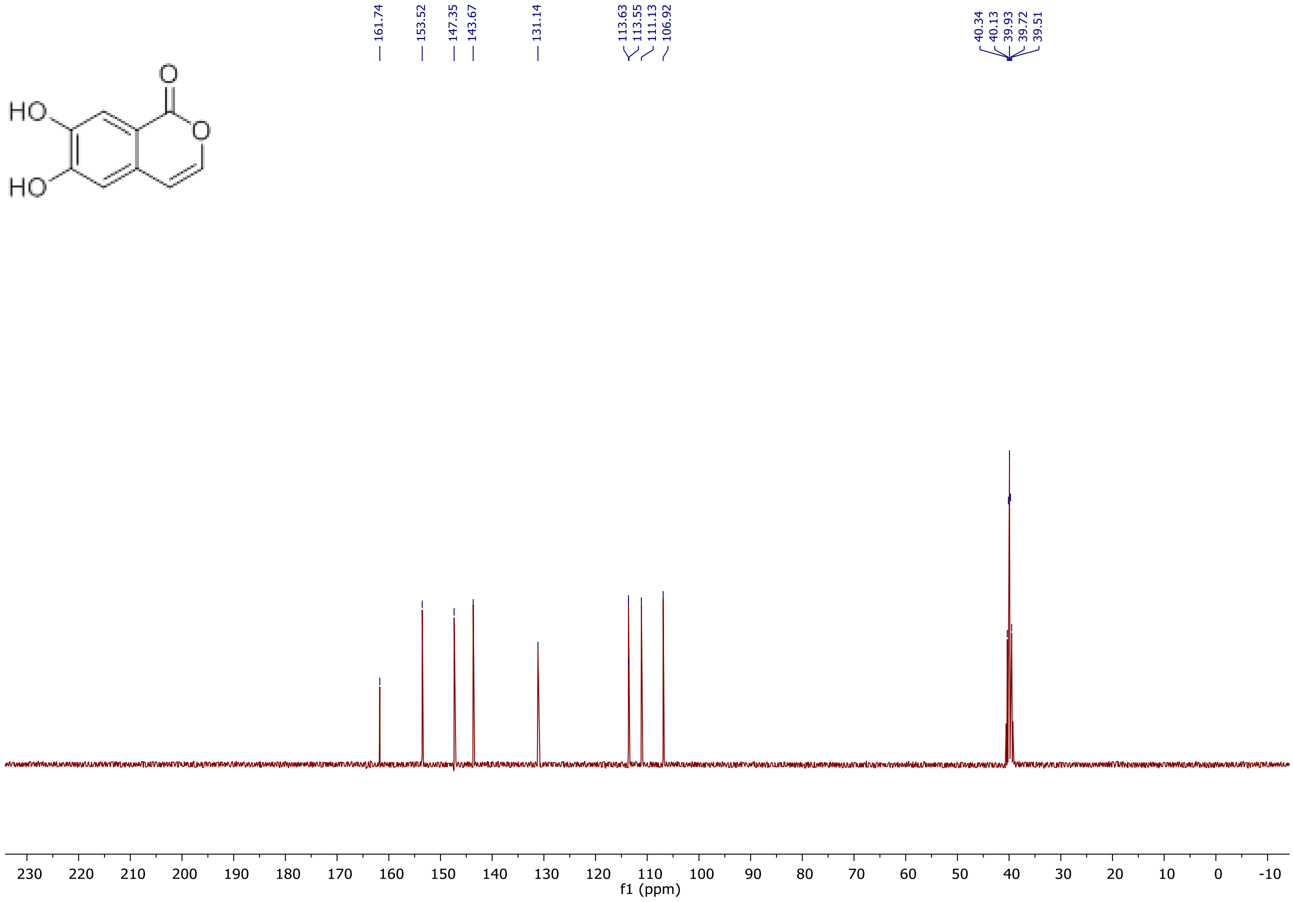
161.74
153.52
147.35
143.67
131.14
113.63
113.55
111.13
106.92
40.34
40.13
39.93
39.72
39.51
HO
HO
O
O
f1 (ppm)

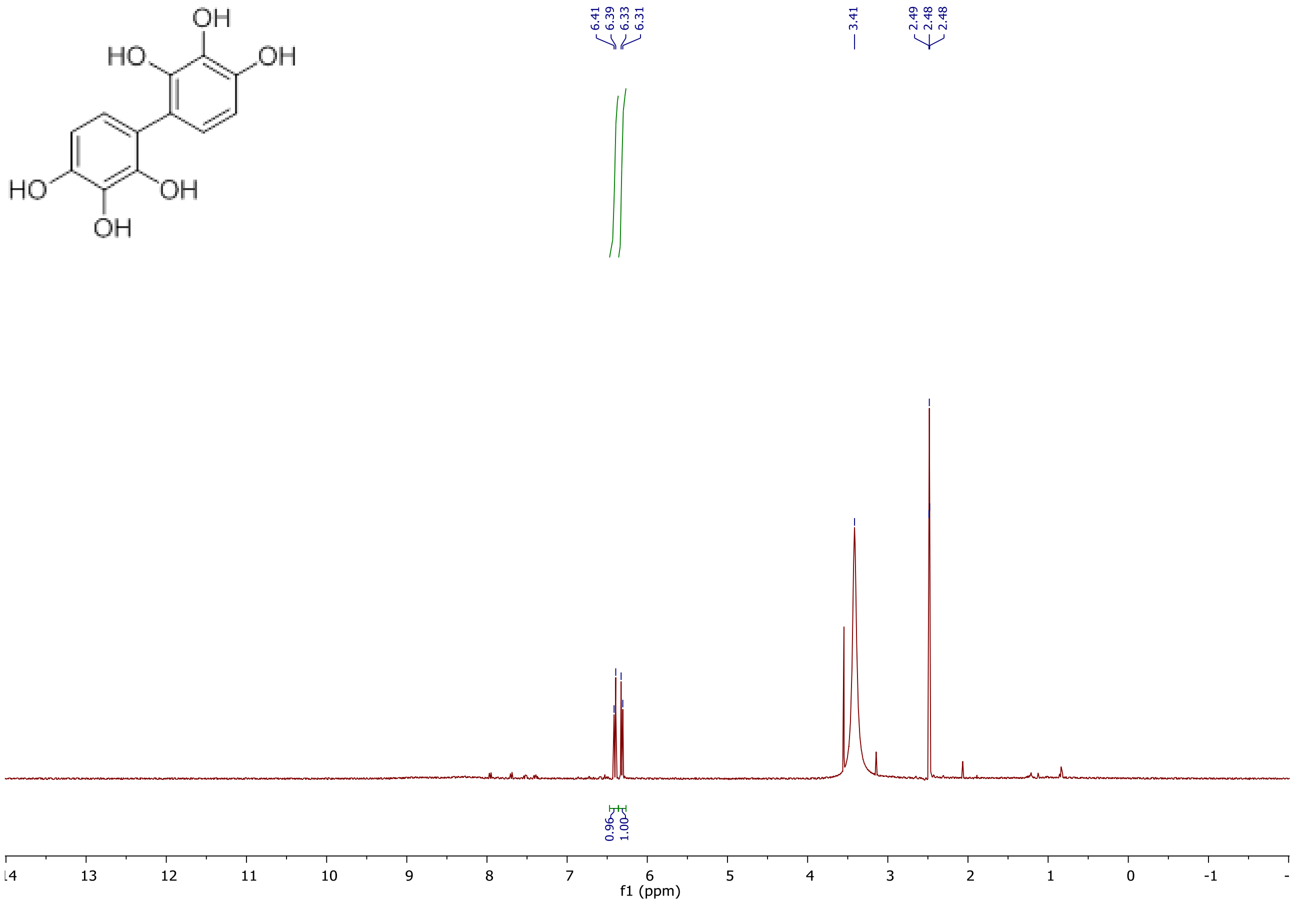
OH
HO
OH
HO
OH
OH
6.41
6.39
6.33
6.31
3.41
2.49
2.48
2.48
0.96
1.00
f1 (ppm)

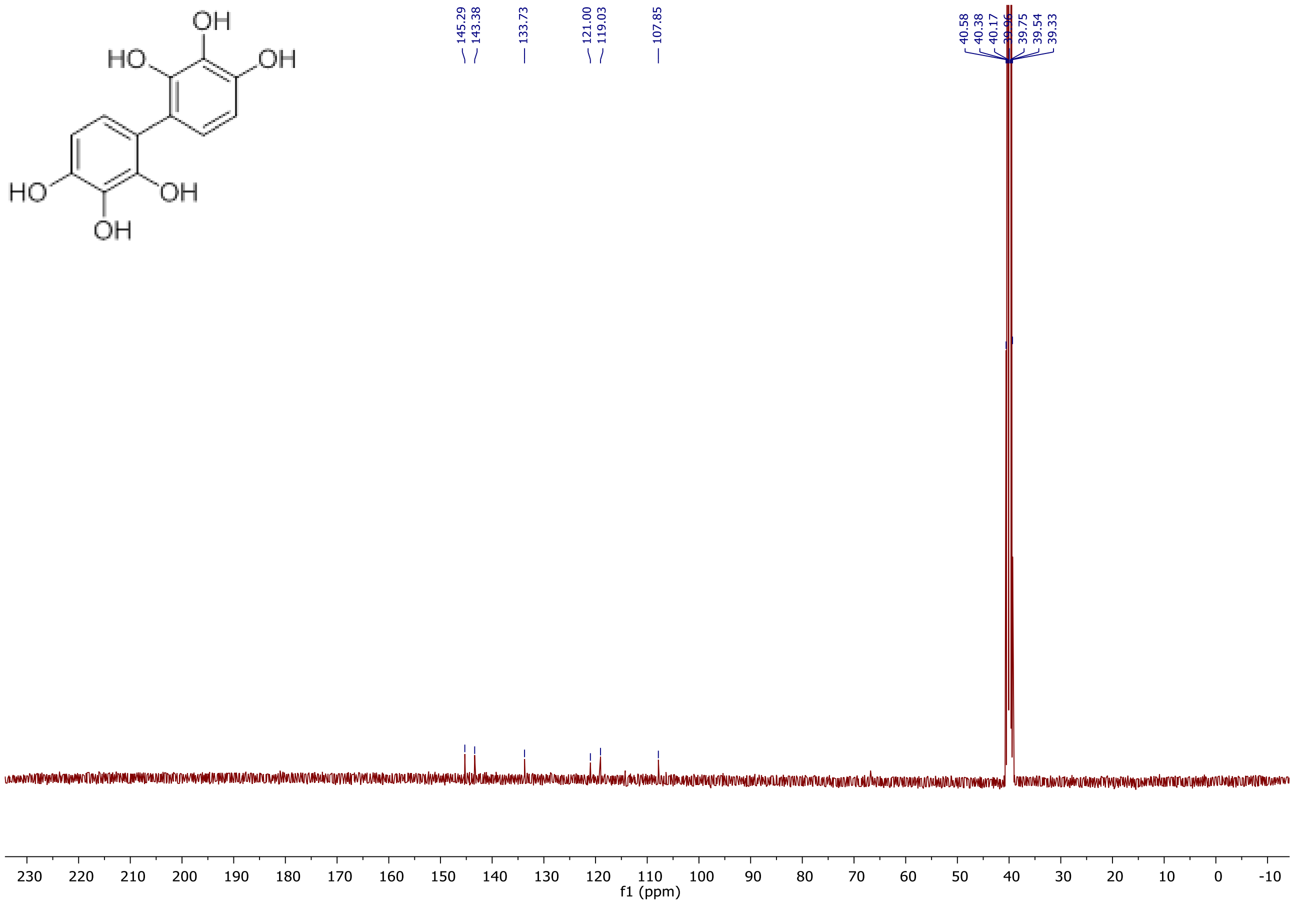
OH
HO
OH
HO
OH
OH
145.29
143.38
133.73
121.00
119.03
107.85
40.58
40.38
40.17
39.96
39.75
39.54
39.33
f1 (ppm)

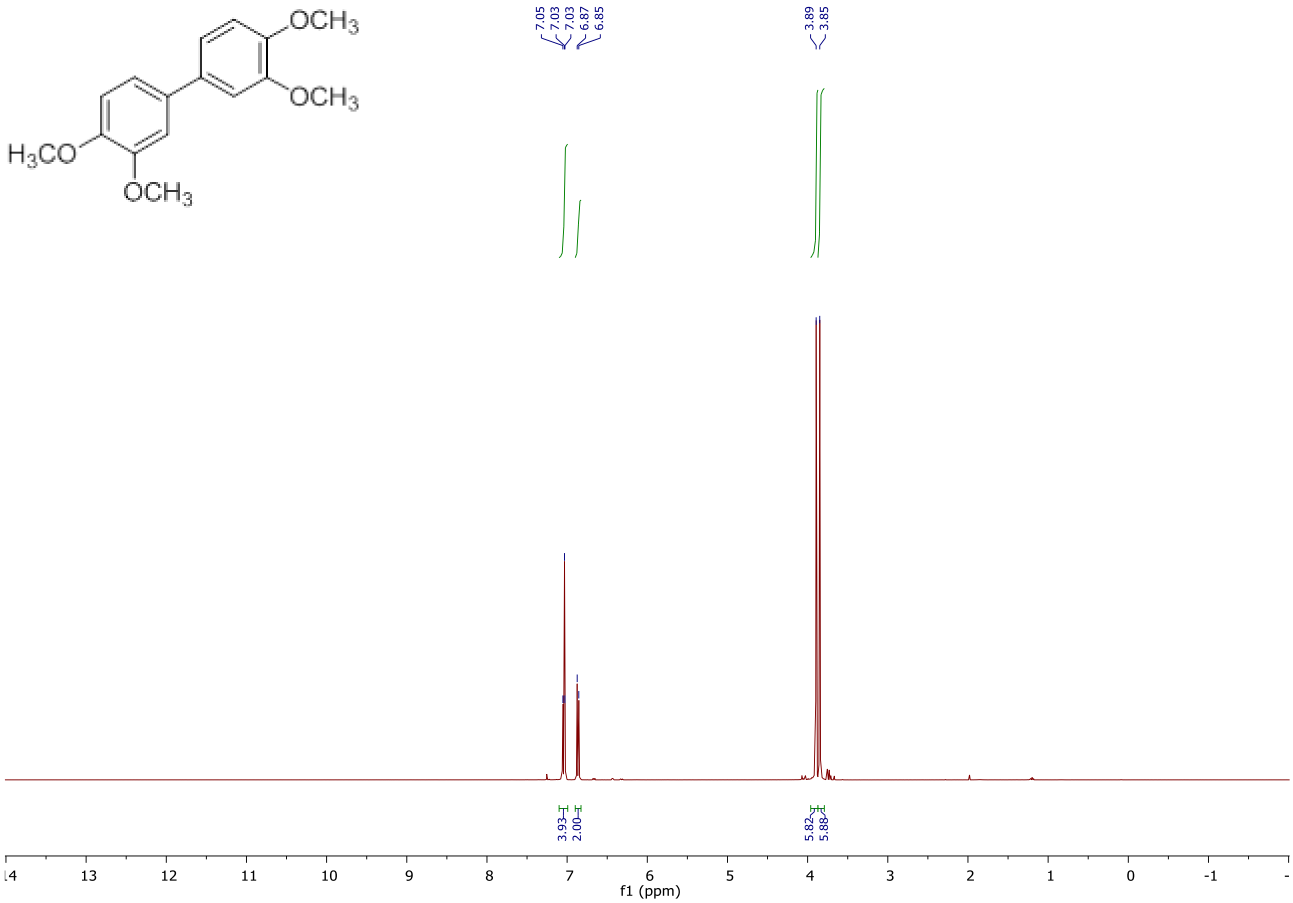
OCH3
OCH3
H3CO
OCH3
7.05
7.03
7.03
6.87
6.85
3.89
3.85
3.93
2.00
5.82
5.88
f1 (ppm)

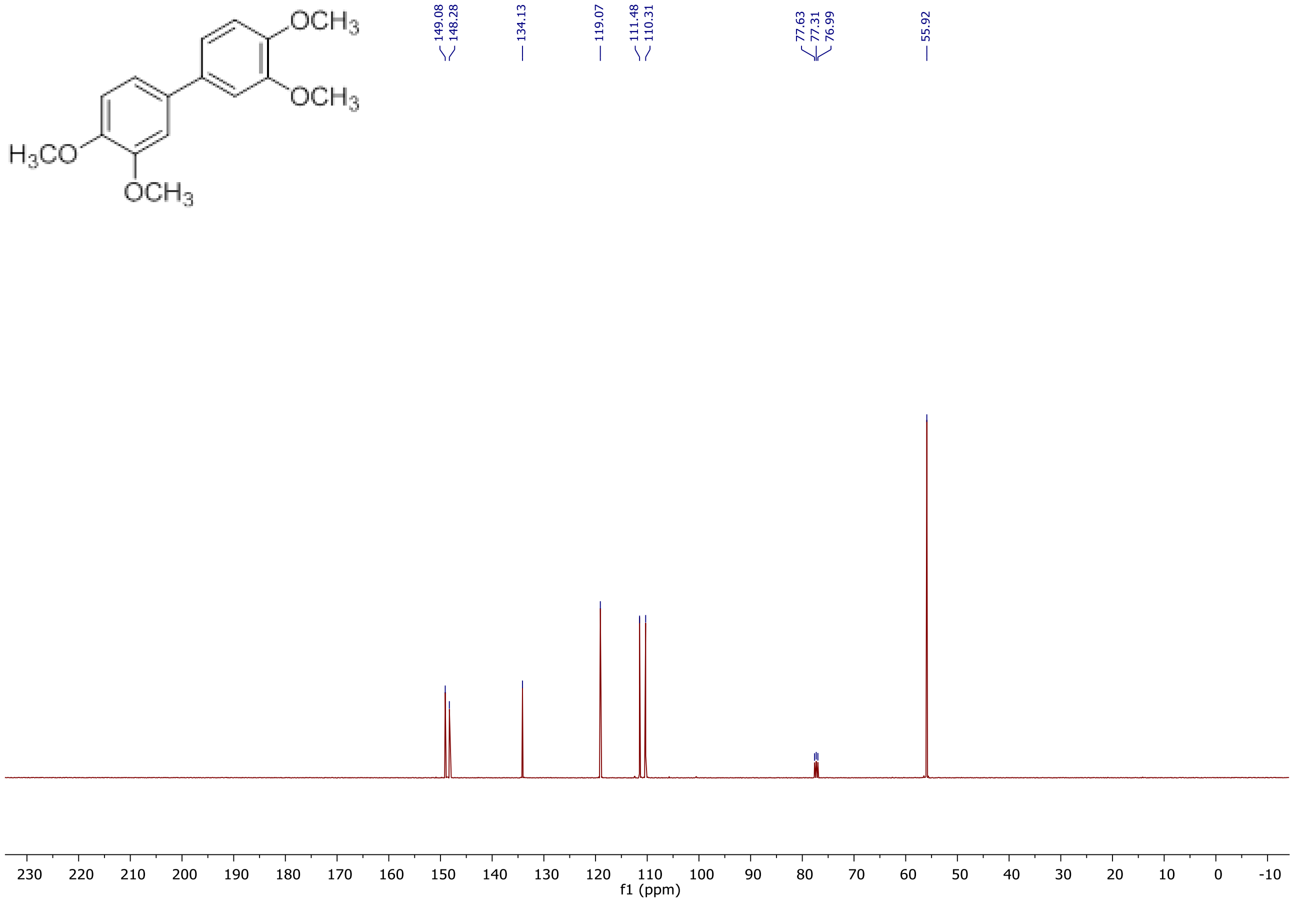
OCH3
OCH3
H3CO
OCH3
149.08
148.28
134.13
119.07
111.48
110.31
77.63
77.31
76.99
55.92
f1 (ppm)

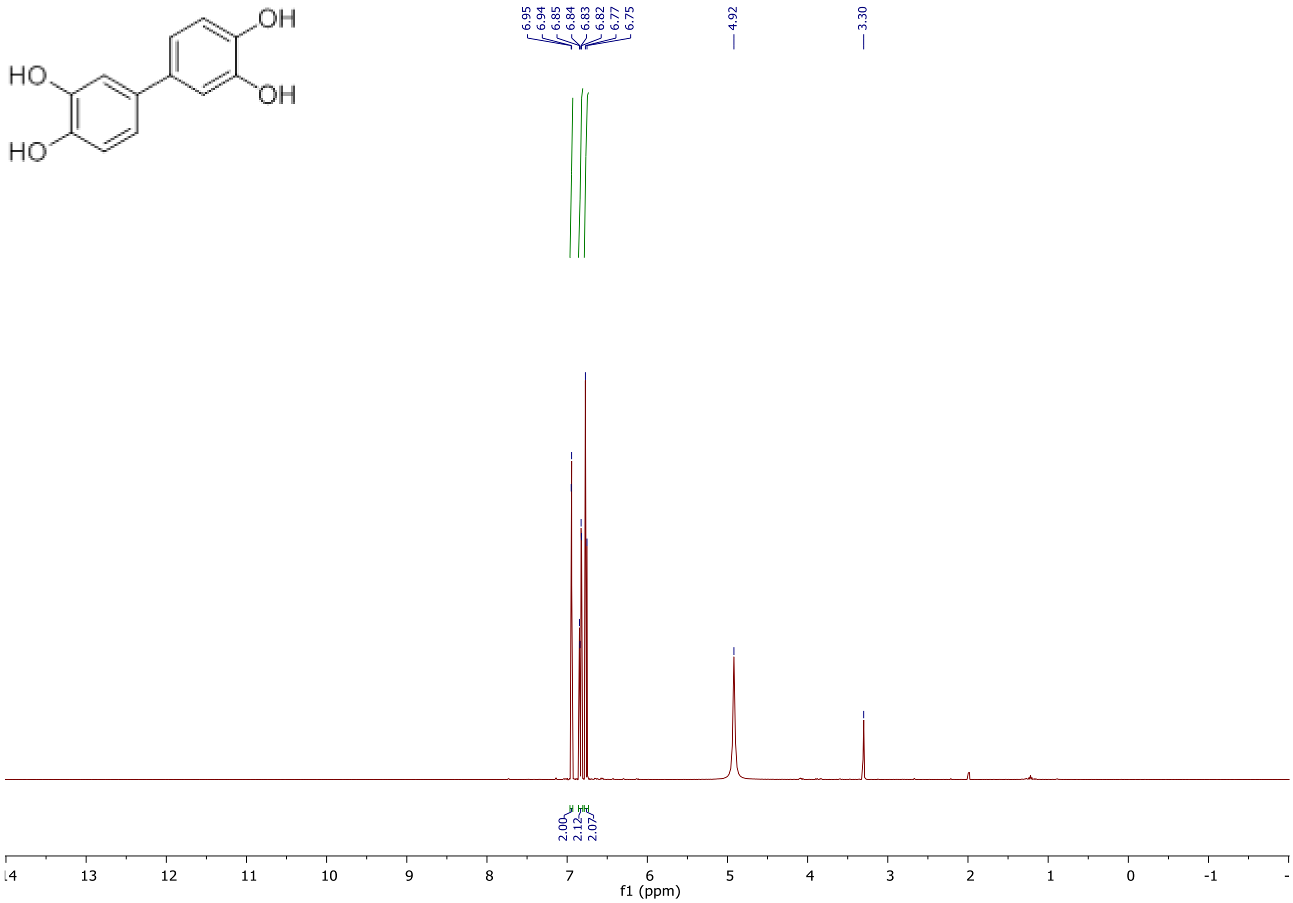
OH
HO
OH
HO
6.95
6.94
6.85
6.84
6.83
6.82
6.77
6.75
4.92
3.30
2.00
2.12
2.07
f1 (ppm)

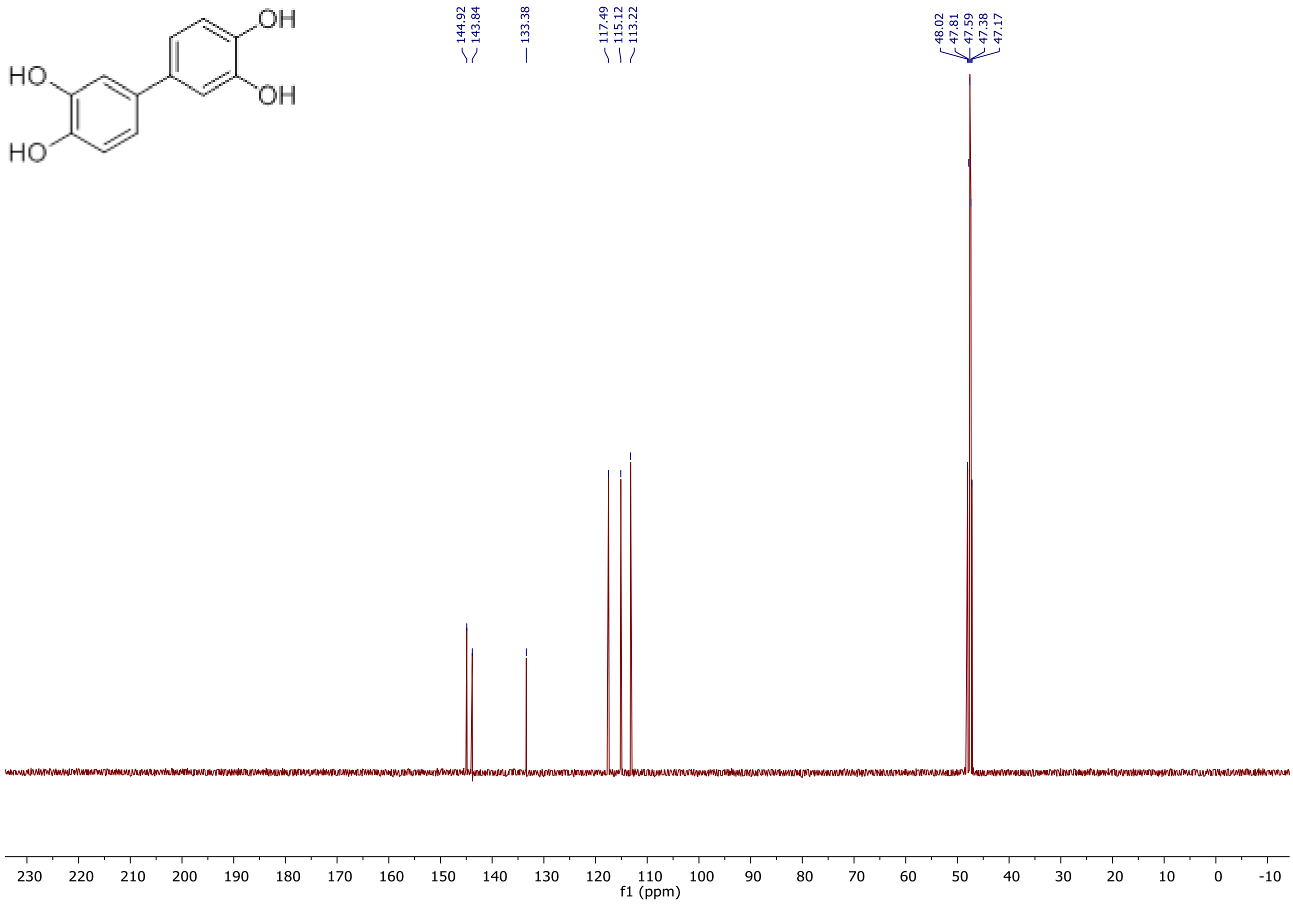
OH
HO
OH
HO
144.92
143.84
133.38
117.49
115.12
113.22
48.02
47.81
47.59
47.38
47.17
f1 (ppm)

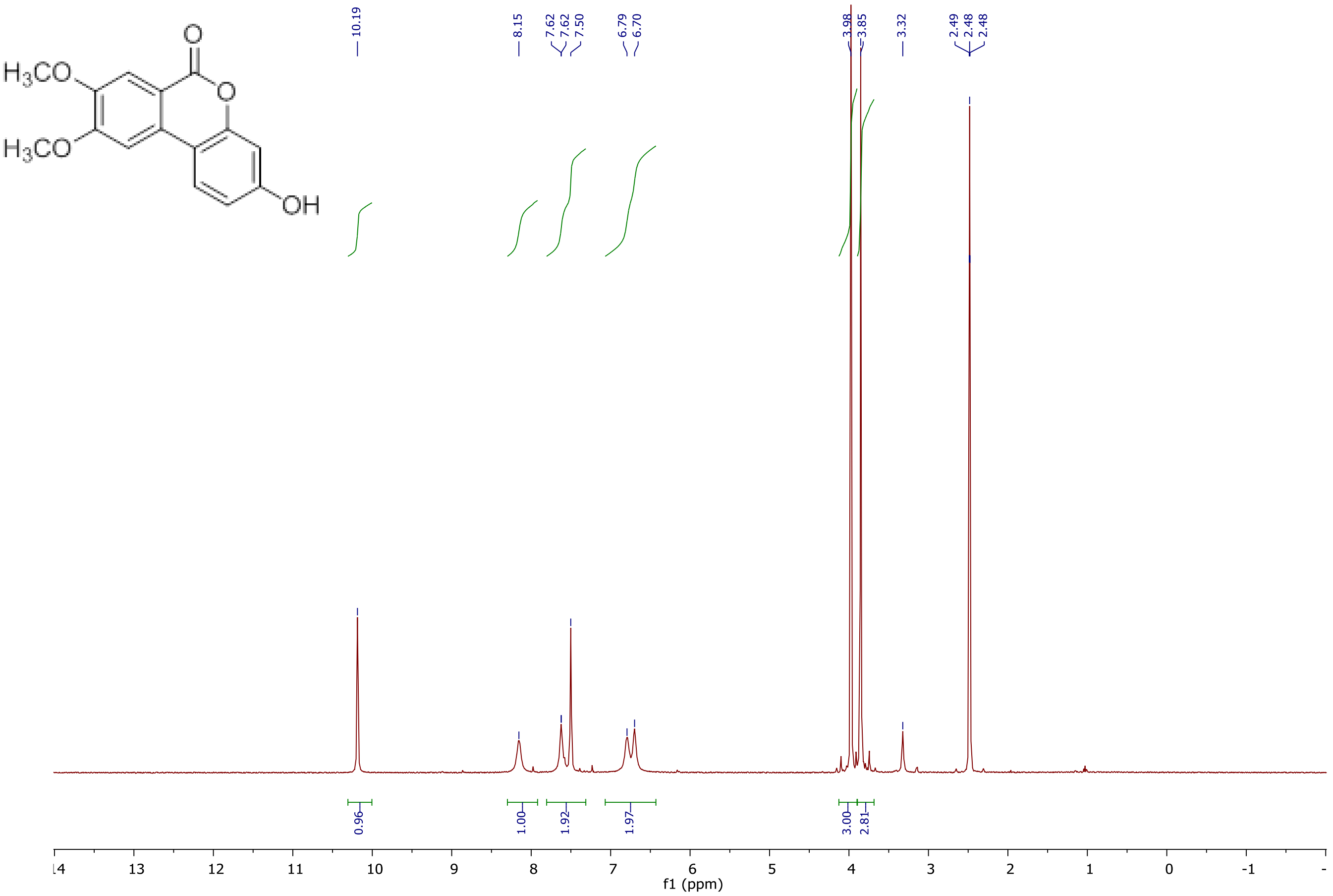
H3CO
H3CO
O
O
OH
10.19
8.15
7.62
7.62
7.50
6.79
6.70
3.98
3.85
3.32
2.49
2.48
2.48
0.96
1.00
1.92
1.97
3.00
2.81
f1 (ppm)

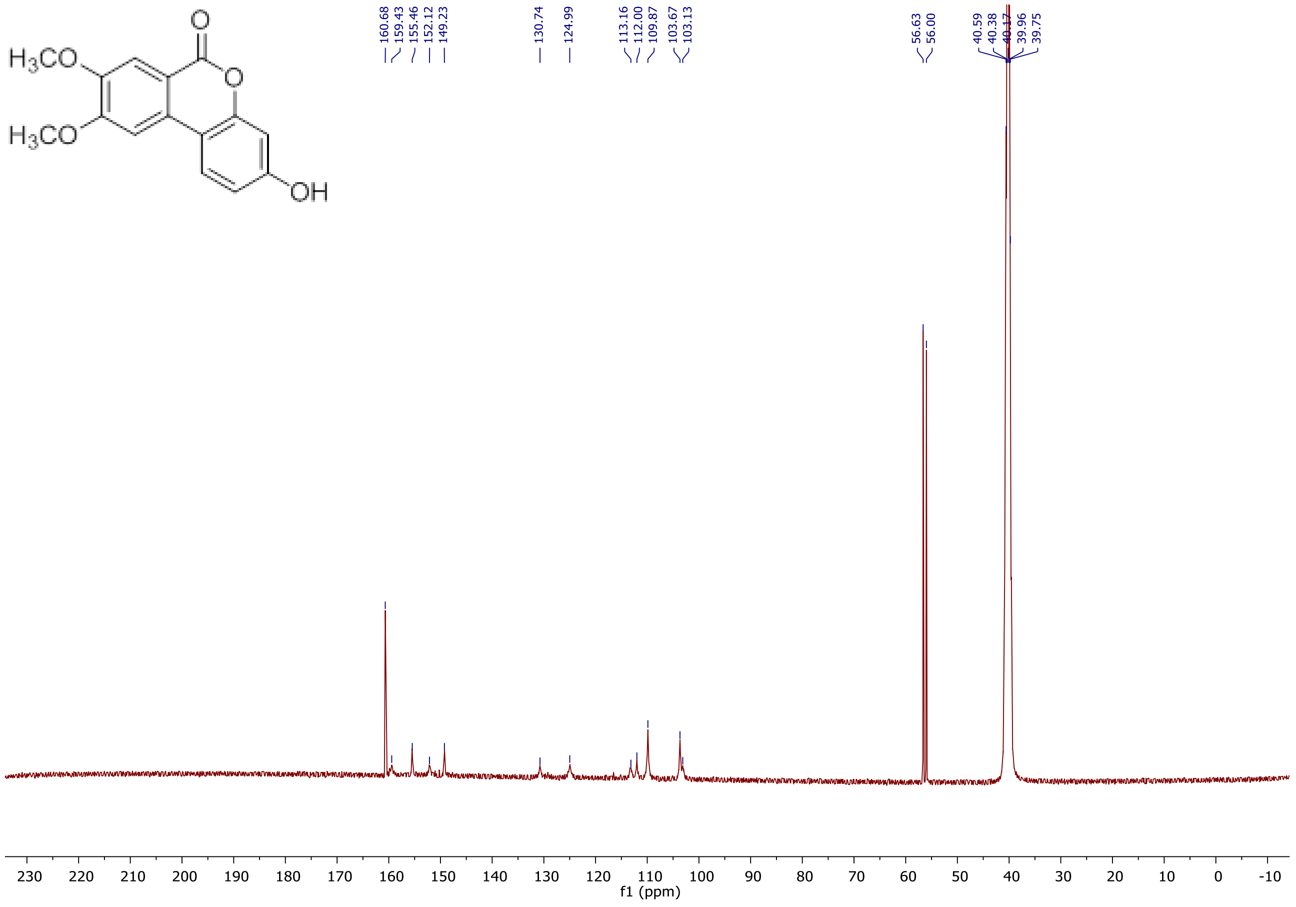
H3CO
H3CO
O
O
OH
160.68
159.43
155.46
152.12
149.23
130.74
124.99
113.16
112.00
109.87
103.67
103.13
56.63
56.00
40.59
40.38
40.17
39.96
39.75
f1 (ppm)

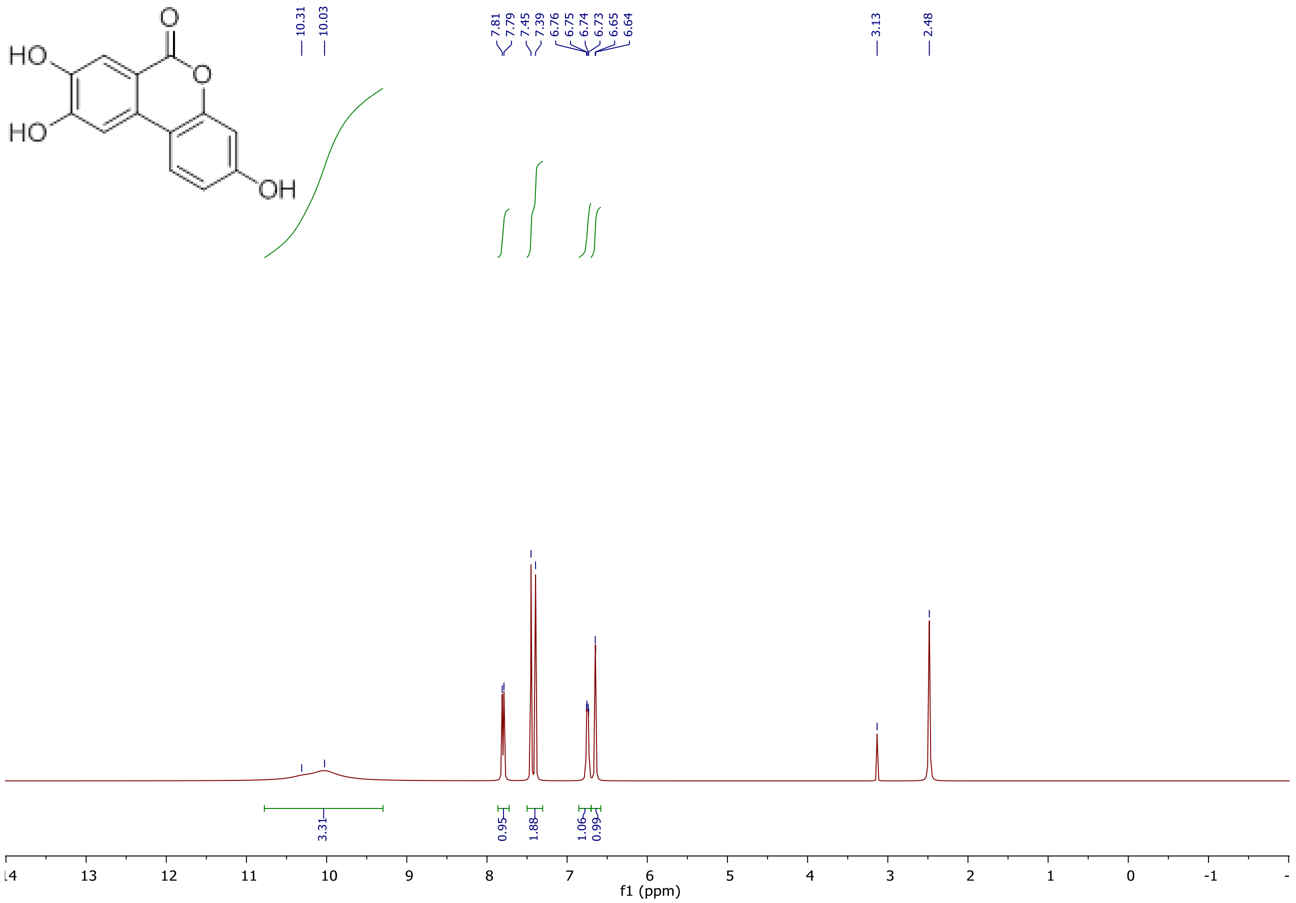
HO
HO
OH
O
10.31
10.03
7.81
7.79
7.45
7.39
6.76
6.75
6.74
6.73
6.65
6.64
3.13
2.48
3.31
0.95
1.88
1.06
0.99
f1 (ppm)

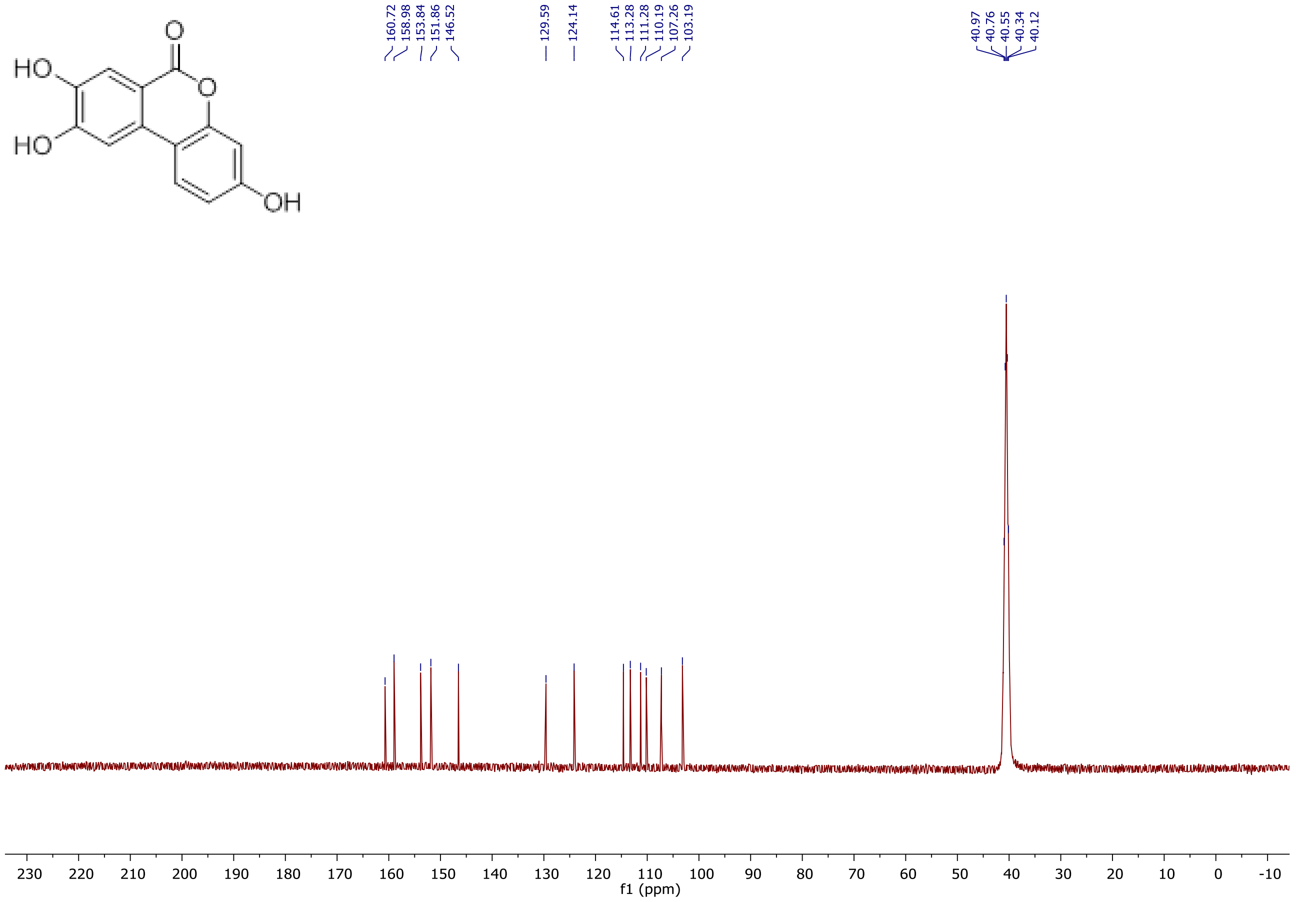
HO
HO
OH
O
160.72
158.98
153.84
151.86
146.52
129.59
124.14
114.61
113.28
111.28
110.19
107.26
103.19
40.97
40.76
40.55
40.34
40.12
f1 (ppm)

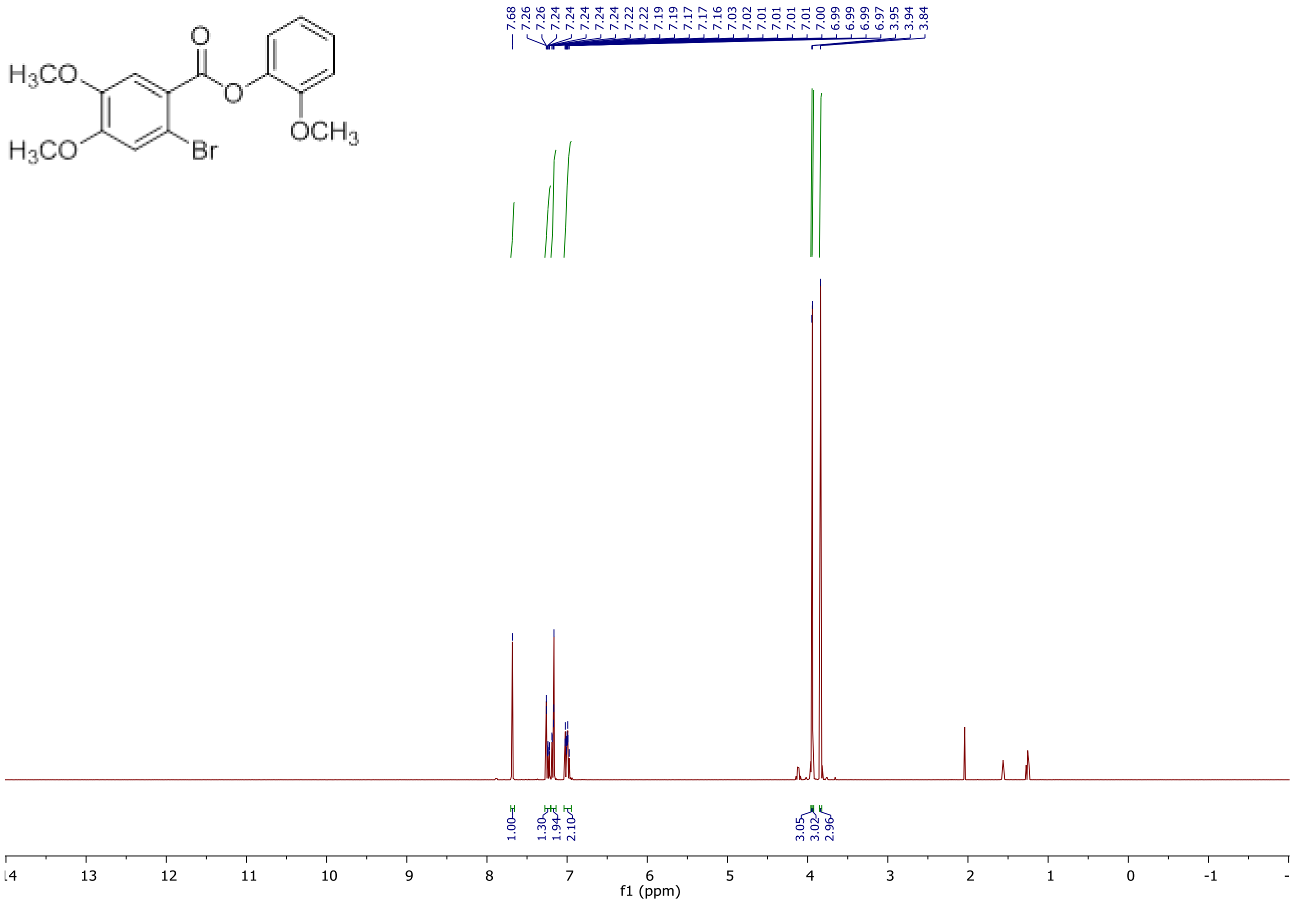
H3CO
H3CO
O
O
Br
OCH3
7.68
7.26
7.24
7.24
7.24
7.24
7.24
7.22
7.22
7.19
7.19
7.17
7.17
7.16
7.03
7.02
7.01
7.01
7.01
7.00
6.99
6.99
6.99
6.97
3.95
3.94
3.84
1.00
1.30
1.94
2.10
3.05
3.02
2.96
f1 (ppm)

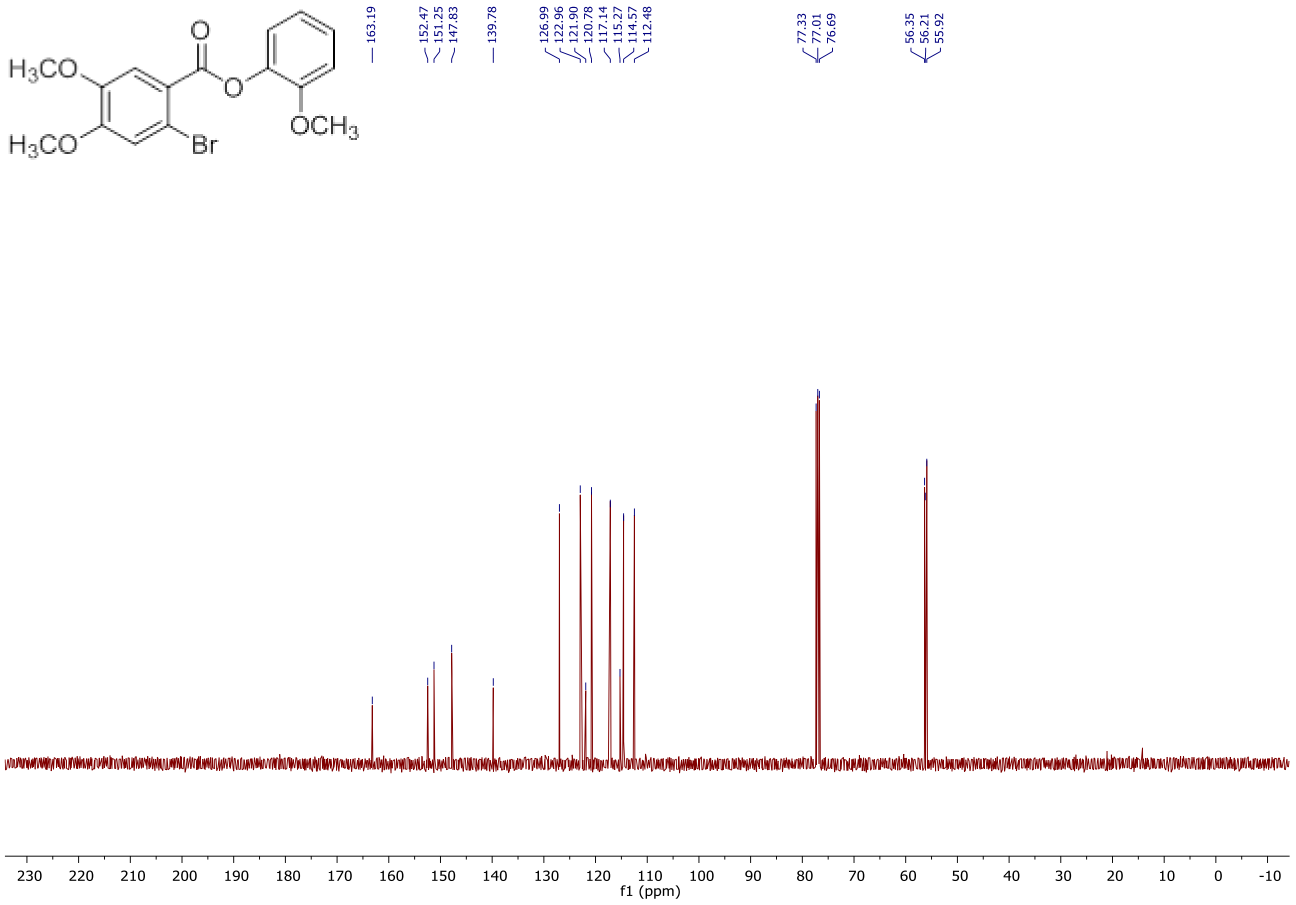
H3CO
H3CO
O
O
Br
OCH3
163.19
152.47
151.25
147.83
139.78
126.99
122.96
121.90
120.78
117.14
115.27
114.57
112.48
77.33
77.01
76.69
56.35
56.21
55.92
f1 (ppm)

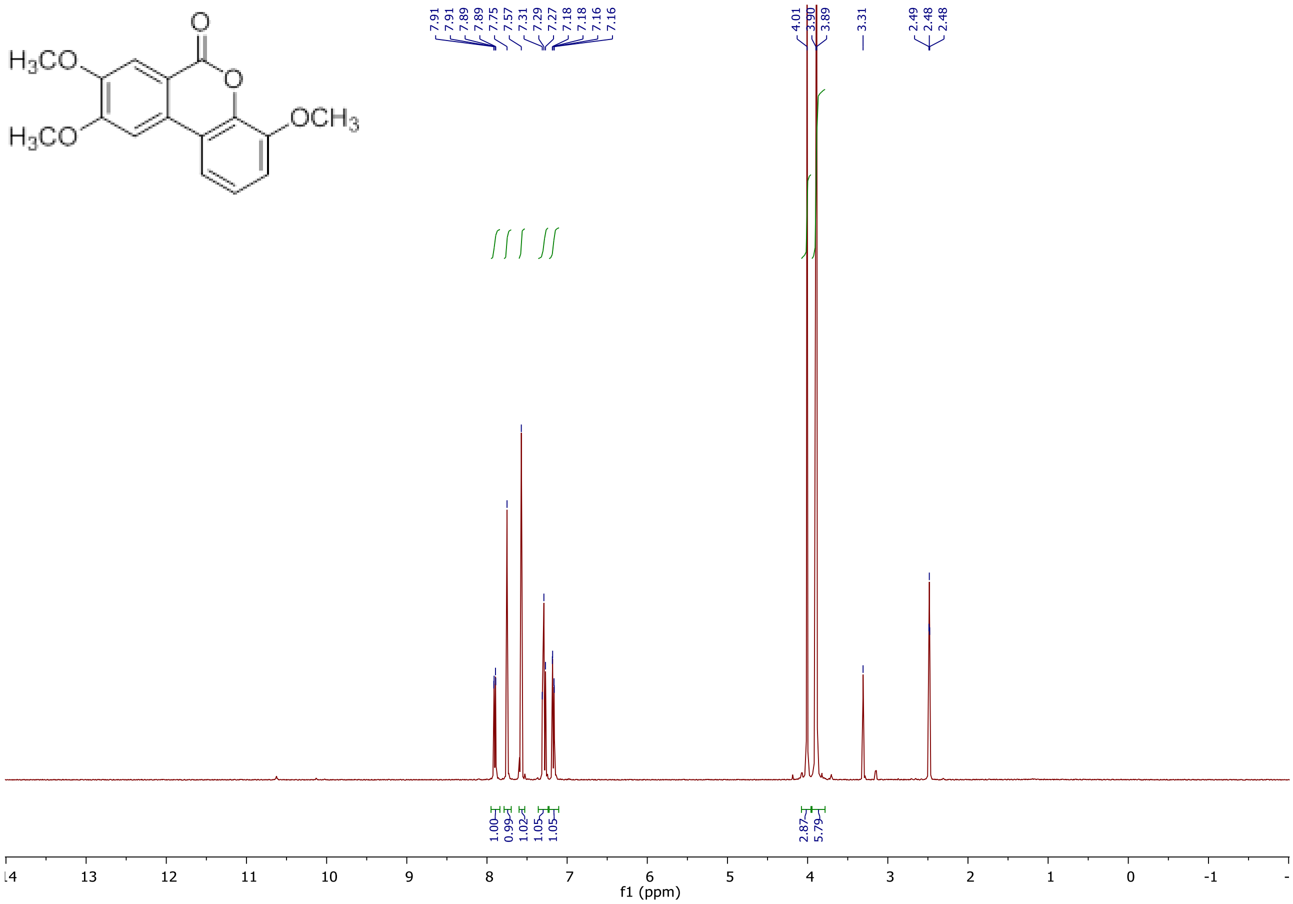
H3CO
H3CO
OCH3
O
O
7.91
7.91
7.89
7.89
7.75
7.57
7.31
7.29
7.27
7.18
7.18
7.16
7.16
4.01
3.90
3.89
3.31
2.49
2.48
2.48
1.00
0.99
1.02
1.05
1.05
2.87
5.79
f1 (ppm)

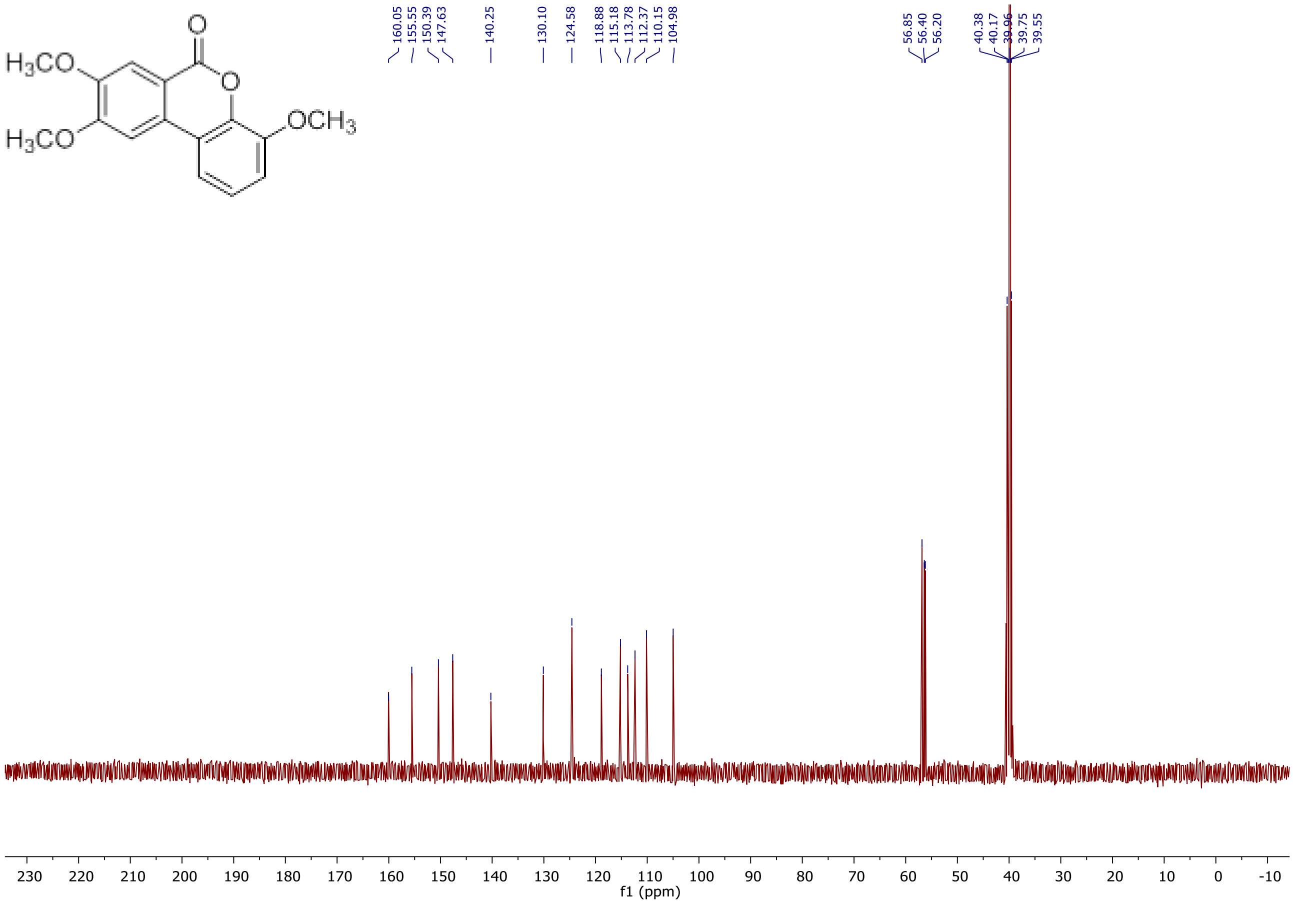
H3CO
H3CO
OCH3
O
O
160.05
155.55
150.39
147.63
140.25
130.10
124.58
118.88
115.18
113.78
112.37
110.15
104.98
56.85
56.40
56.20
40.38
40.17
39.96
39.75
39.55
f1 (ppm)

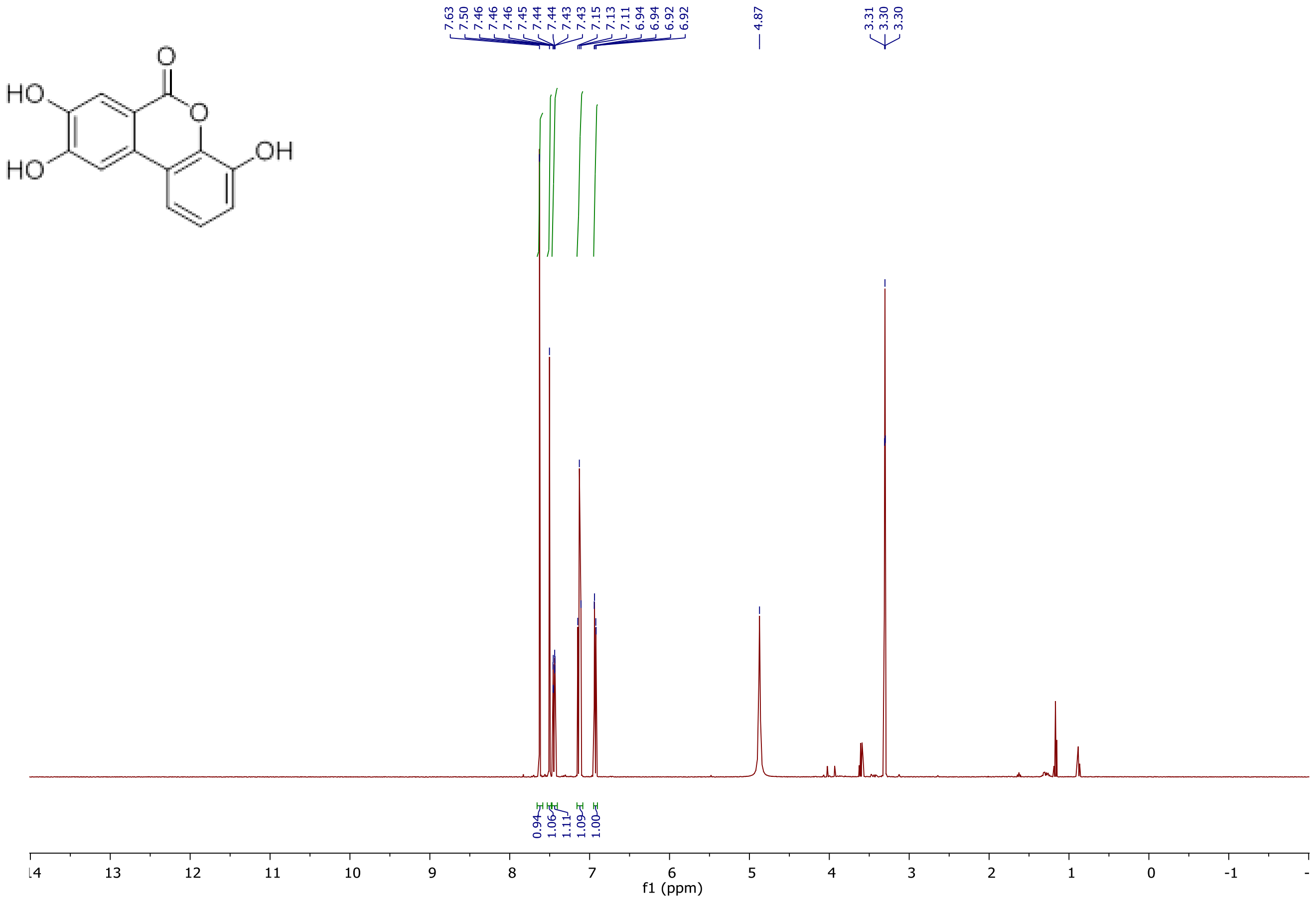
HO
HO
O
O
OH
7.63
7.50
7.46
7.46
7.45
7.44
7.44
7.43
7.43
7.15
7.13
7.11
6.94
6.94
6.92
6.92
4.87
3.31
3.30
3.30
0.94
1.06
1.11
1.09
1.00
f1 (ppm)

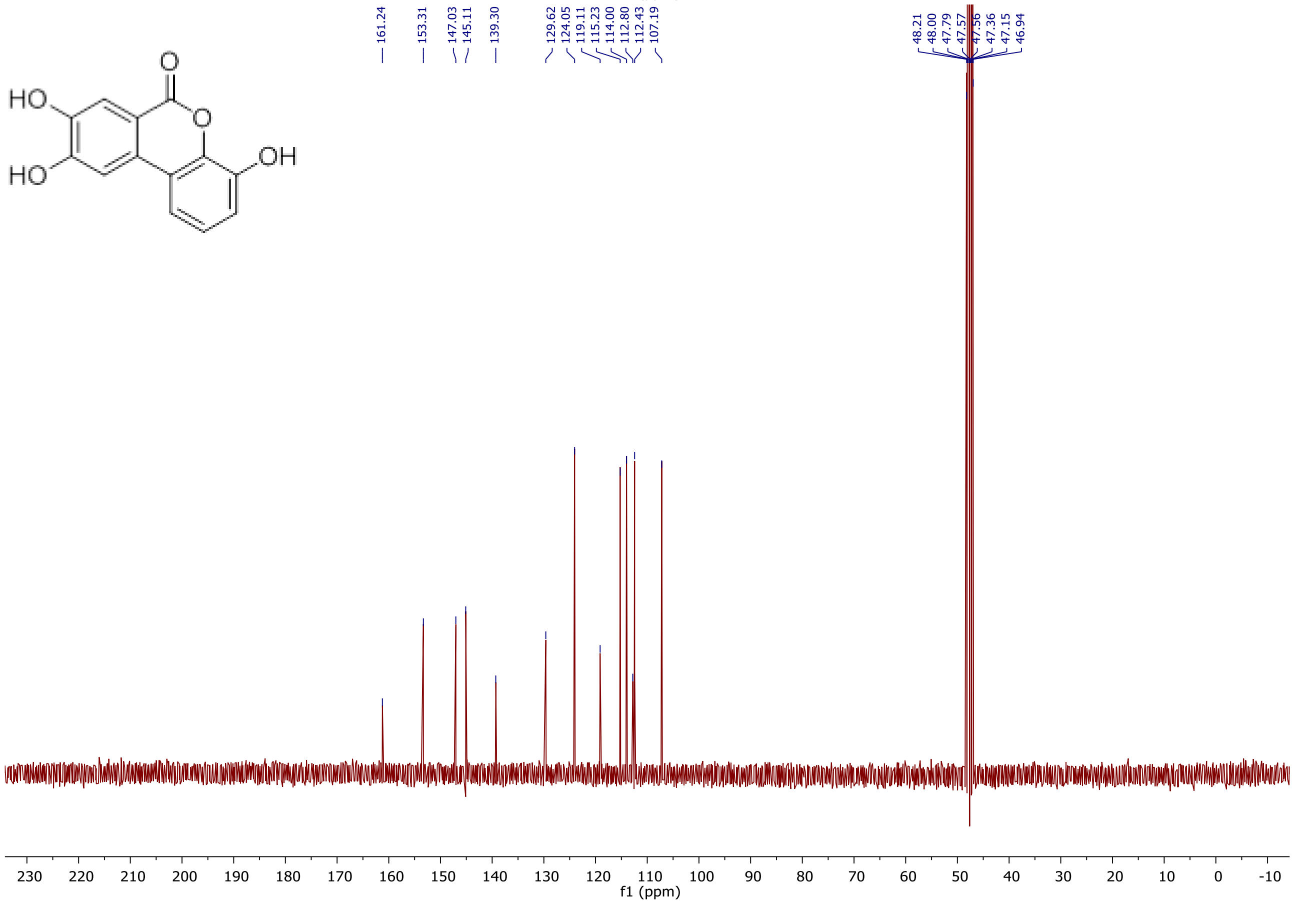
HO
HO
O
O
OH
161.24
153.31
147.03
145.11
139.30
129.62
124.05
119.11
115.23
114.00
112.80
112.43
107.19
48.21
48.00
47.79
47.57
47.56
47.36
47.15
46.94
f1 (ppm)

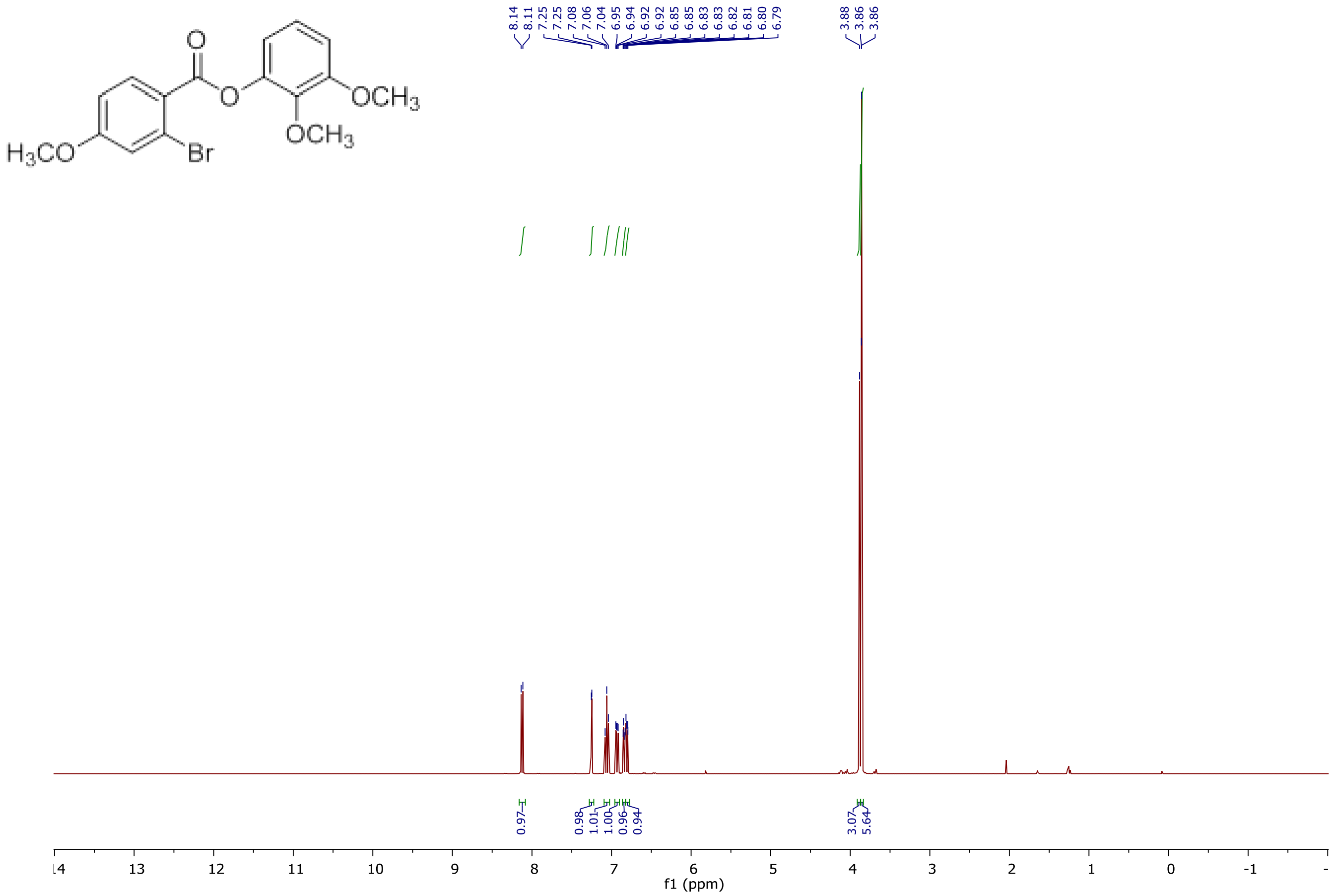
8.14
8.11
7.25
7.25
7.08
7.06
7.04
6.95
6.94
6.92
6.92
6.92
6.85
6.85
6.83
6.83
6.82
6.81
6.80
6.79
3.88
3.86
3.86
OCH3
OCH3
H3CO
Br
0.97
0.98
1.01
1.00
0.96
0.94
3.07
5.64
f1 (ppm)

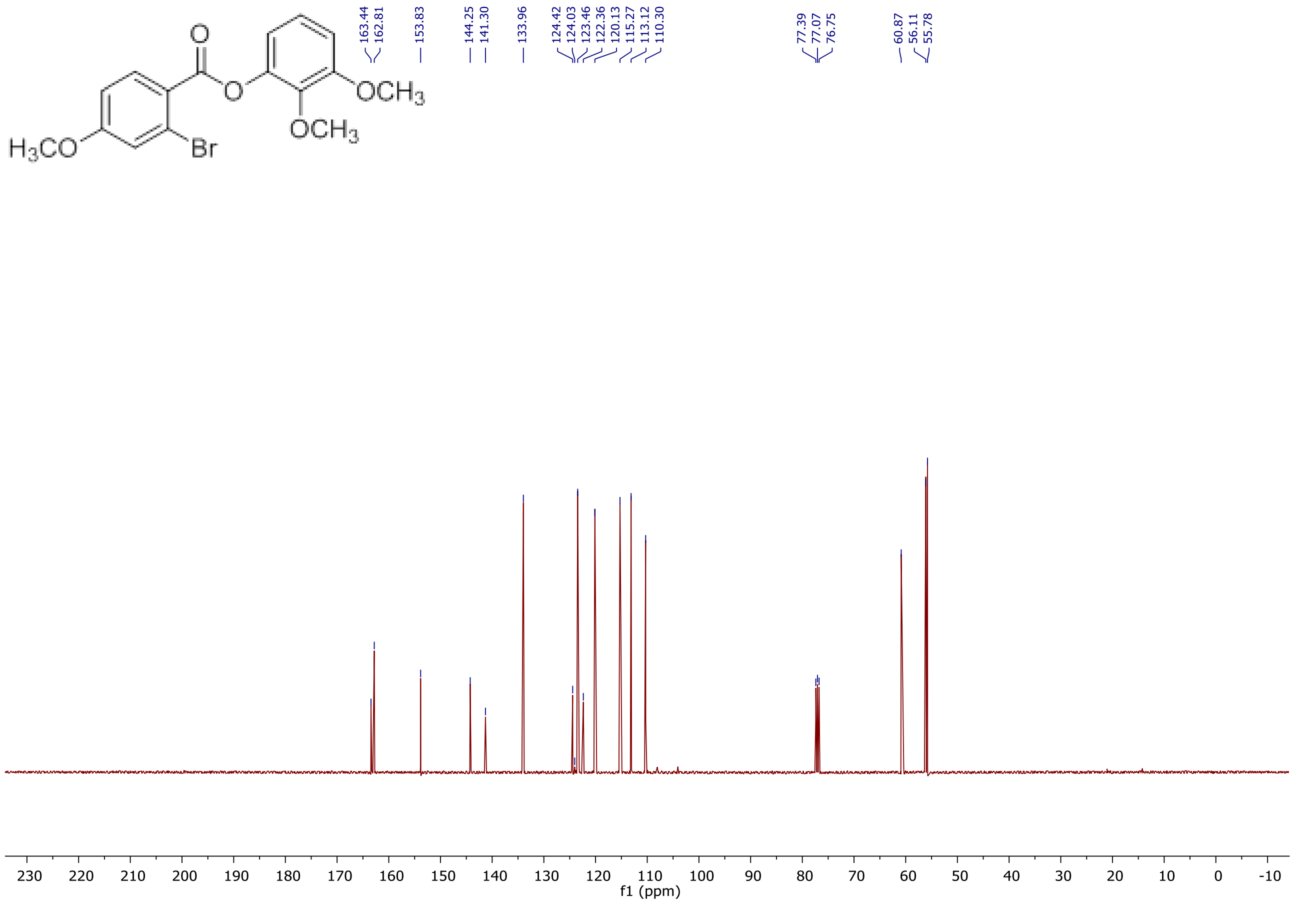
163.44
162.81
153.83
144.25
141.30
133.96
124.42
124.03
123.46
122.36
120.13
115.27
113.12
110.30
77.39
77.07
76.75
60.87
56.11
55.78
OCH3
OCH3
H3CO
Br
f1 (ppm)

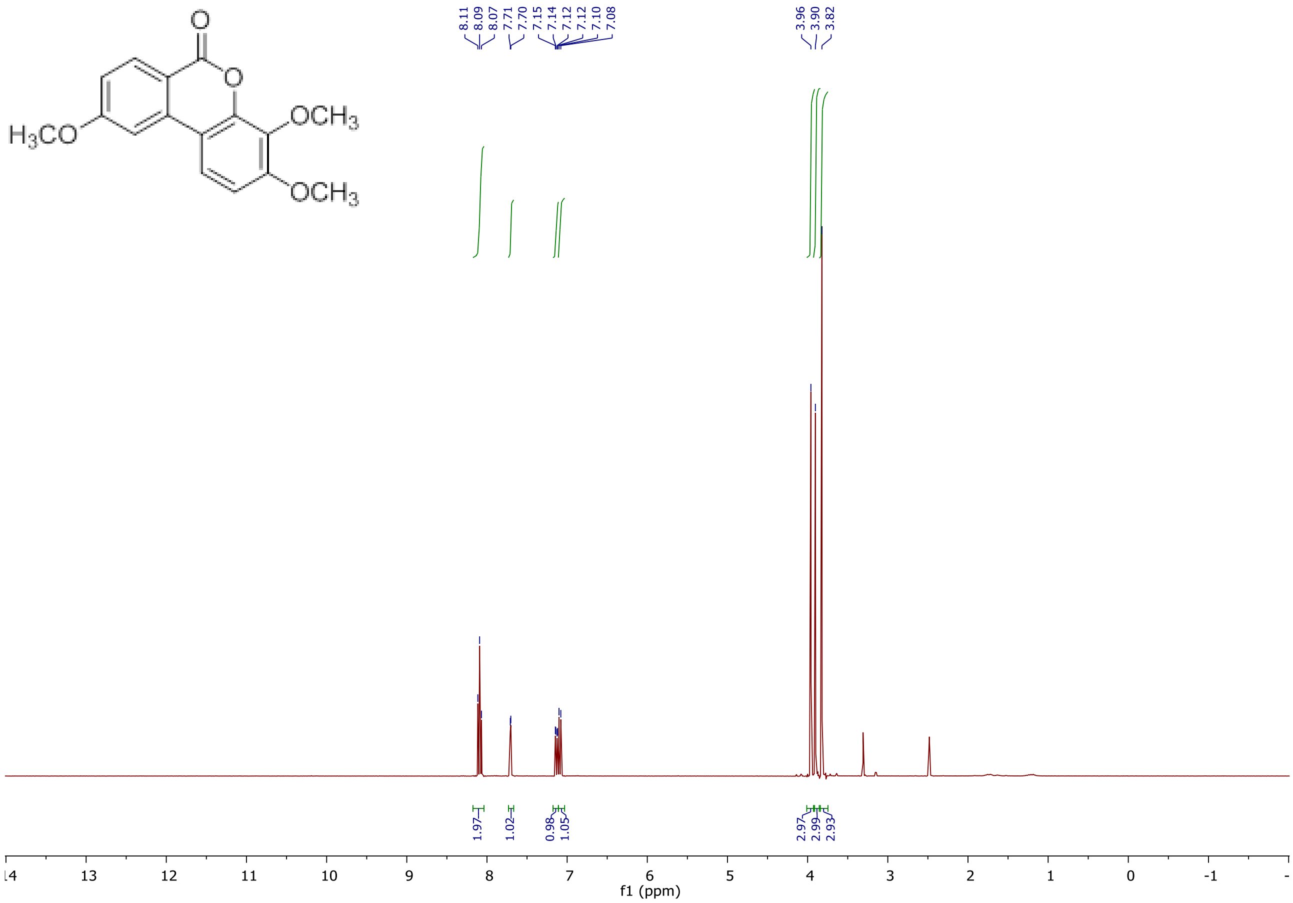

H3CO
OCH3
OCH3
8.11
8.09
8.07
7.71
7.70
7.15
7.14
7.12
7.12
7.10
7.08
3.96
3.90
3.82
1.97
1.02
0.98
1.05
2.97
2.99
2.93
14 13 12 11 10 9 8 7 6 5 4 3 2 1 0 -1
f1 (ppm)

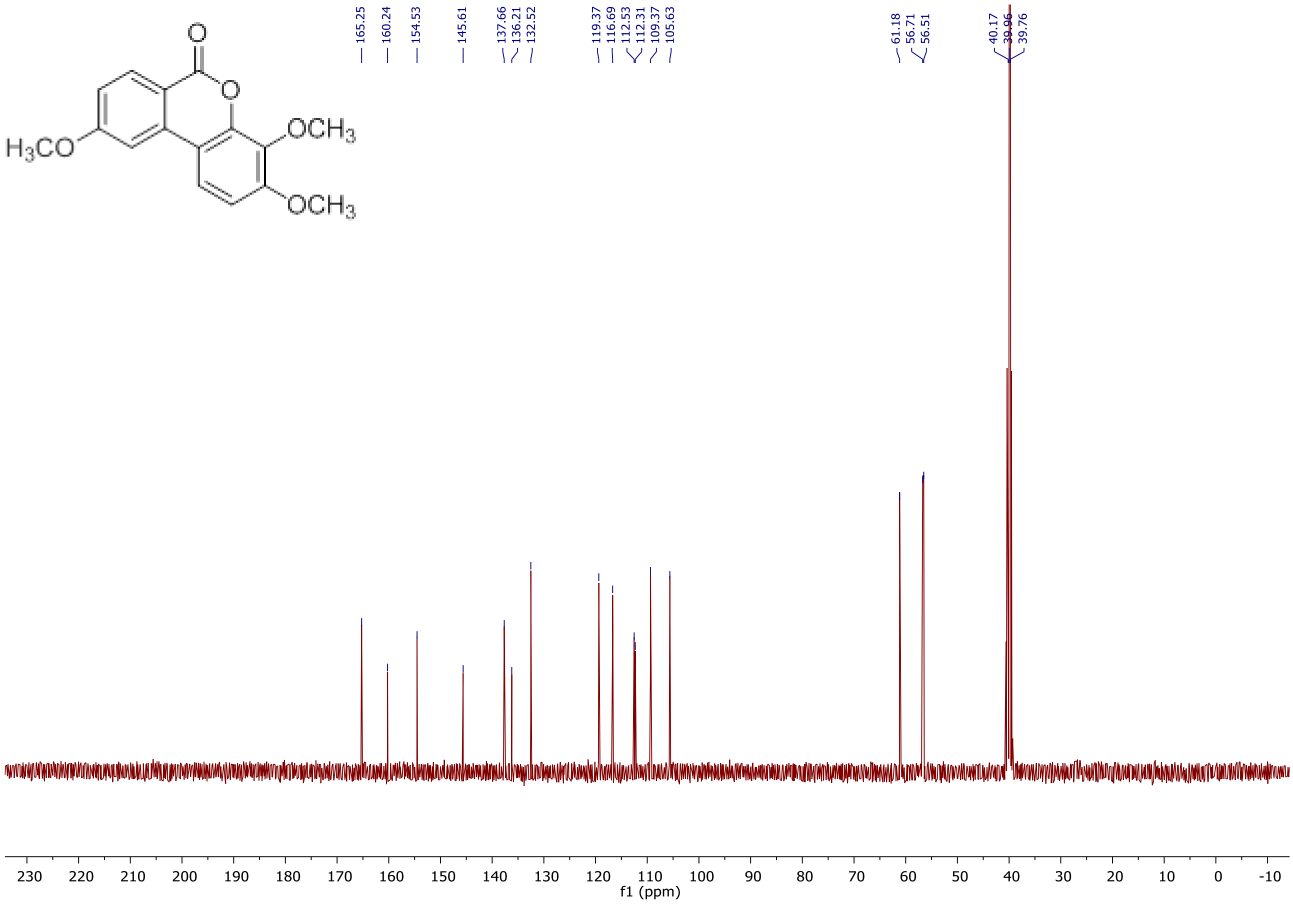

H3CO
OCH3
OCH3
165.25
160.24
154.53
145.61
137.66
136.21
132.52
119.37
116.69
112.53
112.31
109.37
105.63
61.18
56.71
56.51
40.17
39.96
39.76
230 220 210 200 190 180 170 160 150 140 130 120 110 100 90 80 70 60 50 40 30 20 10 0 -10
f1 (ppm)

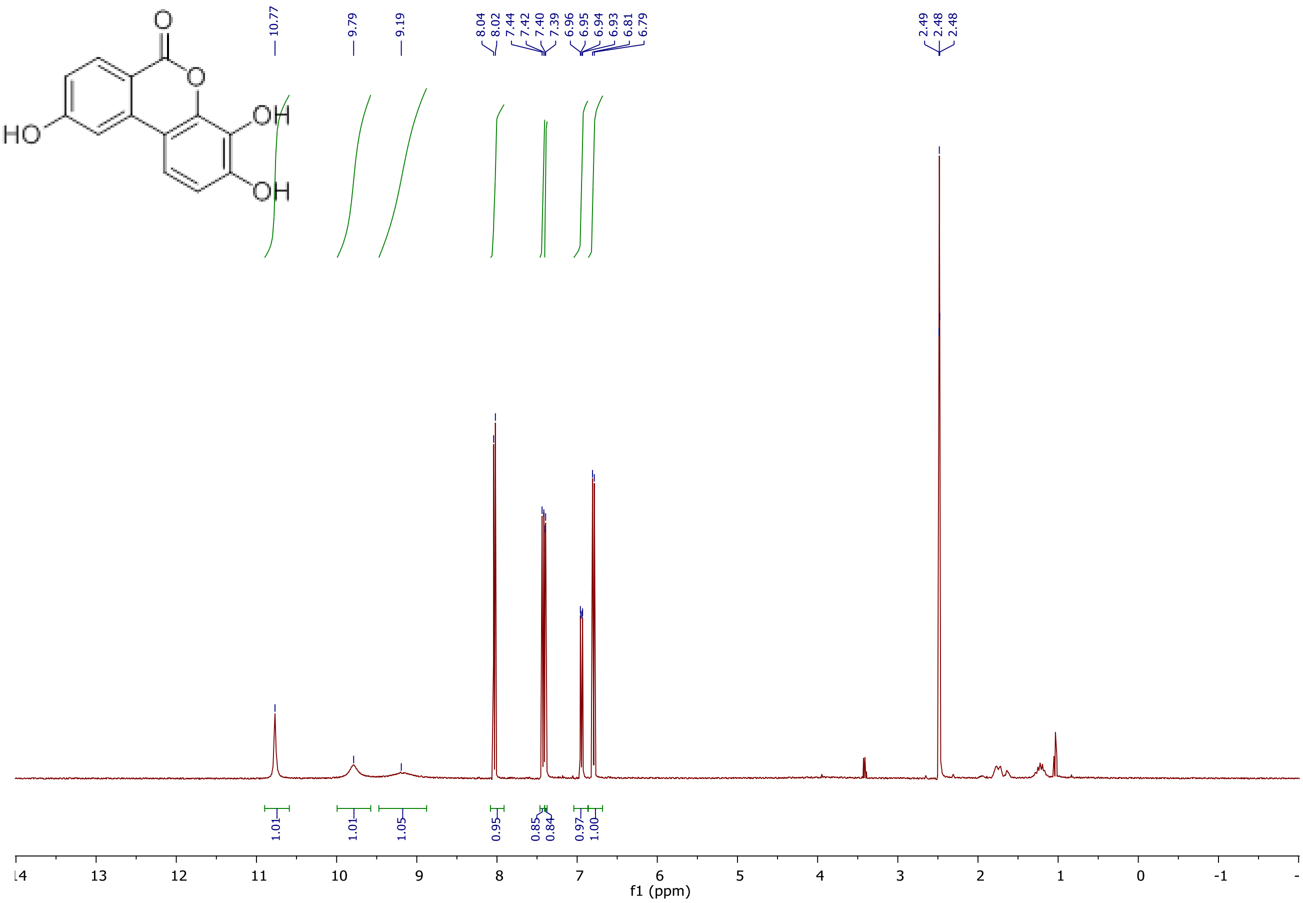

HO
OH
OH
O
10.77
9.79
9.19
8.04
8.02
7.44
7.42
7.40
7.39
6.96
6.95
6.94
6.93
6.81
6.79
2.49
2.48
2.48
1.01
1.01
1.05
0.95
0.85
0.84
0.97
1.00
f1 (ppm)

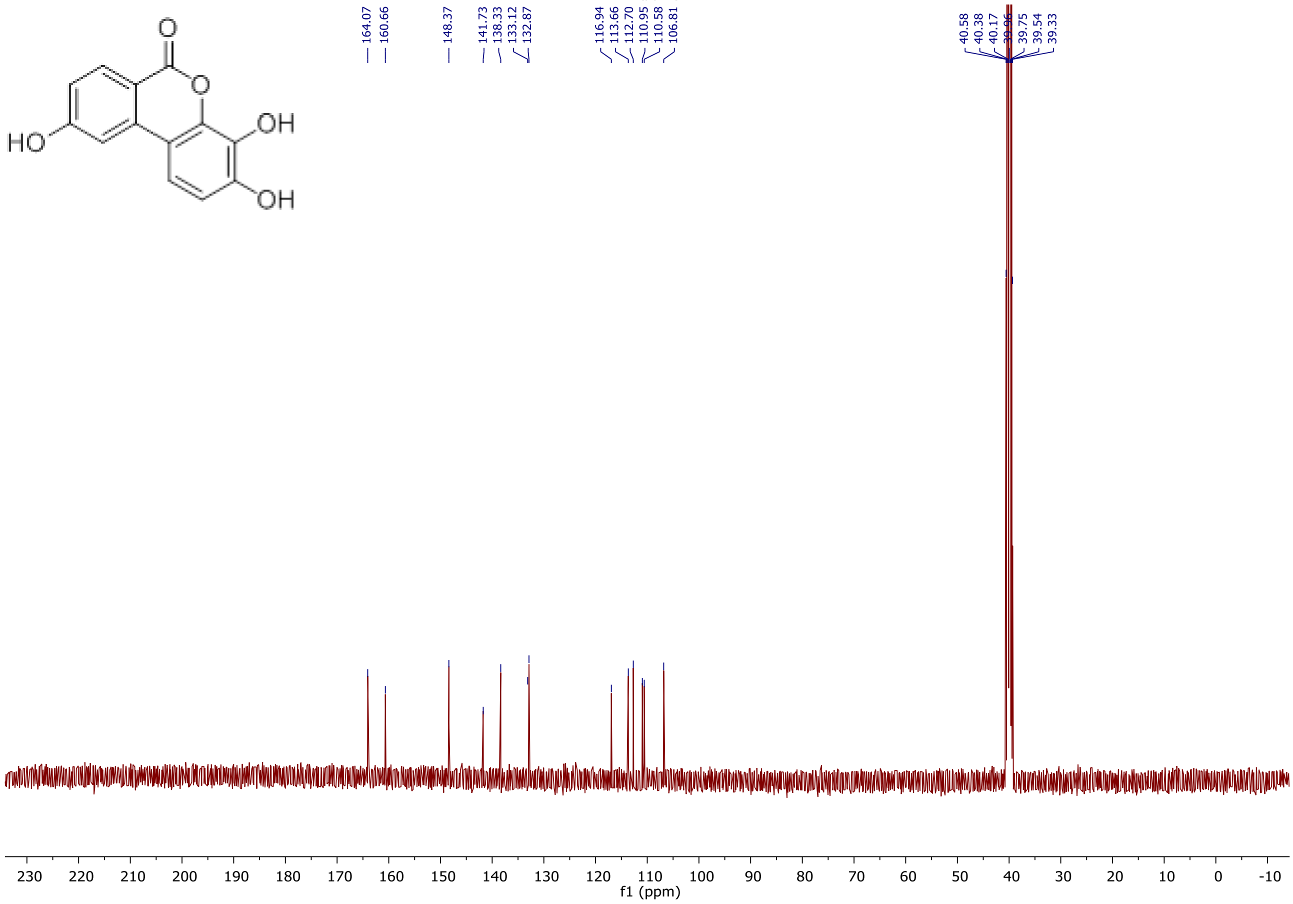

HO
OH
OH
O
164.07
160.66
148.37
141.73
138.33
133.12
132.87
116.94
113.66
112.70
110.95
110.58
106.81
40.58
40.38
40.17
39.96
39.75
39.54
39.33
f1 (ppm)

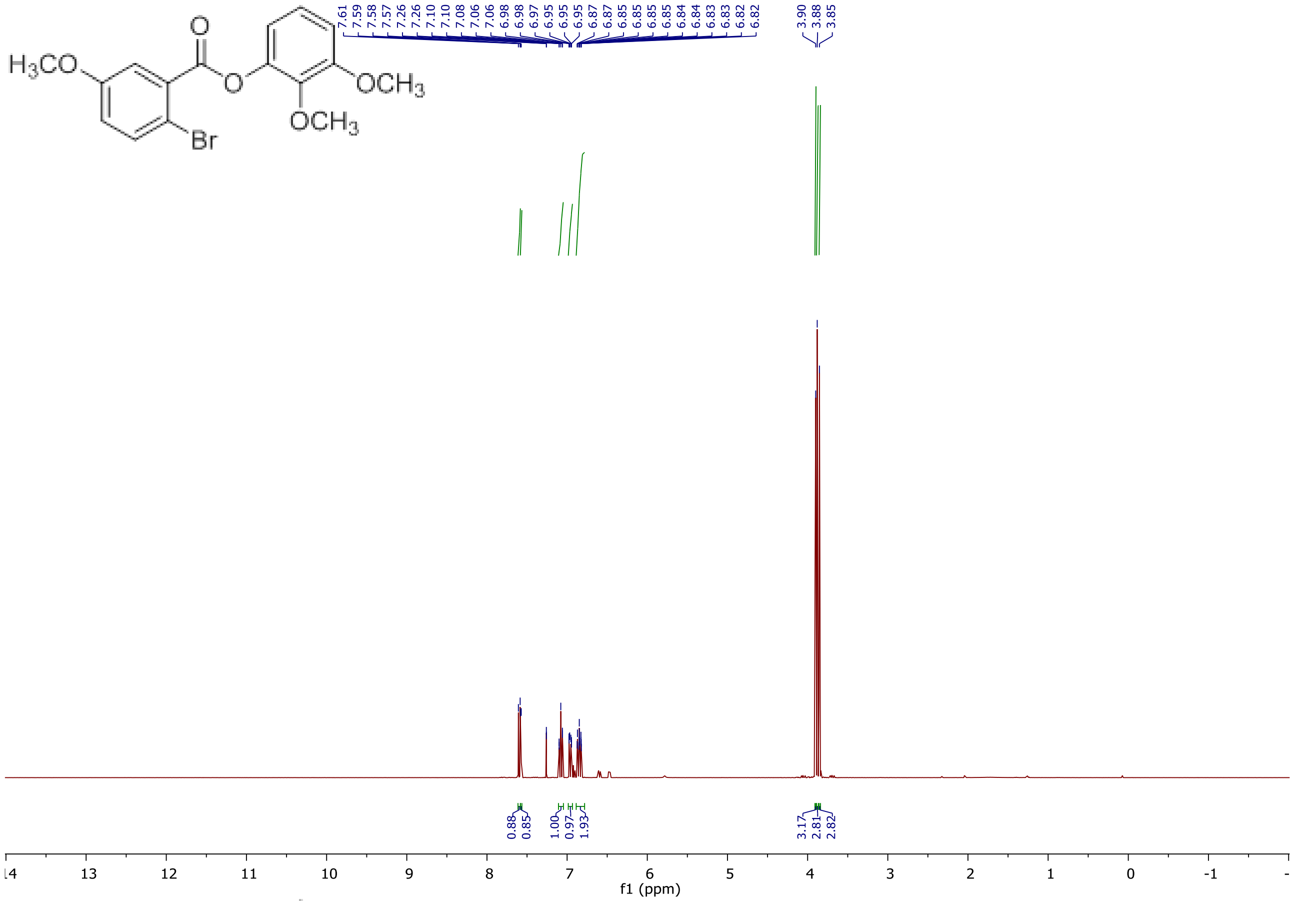
H3CO
O
O
OCH3
OCH3
Br
7.61 7.59 7.58 7.57 7.26 7.10 7.08 7.06 7.06 6.98 6.97 6.95 6.95 6.95 6.87 6.87 6.85 6.85 6.85 6.84 6.83 6.83 6.82 6.82
3.90 3.88 3.85
0.88 0.85 1.00 0.97 1.93 3.17 2.81 2.82
f1 (ppm)

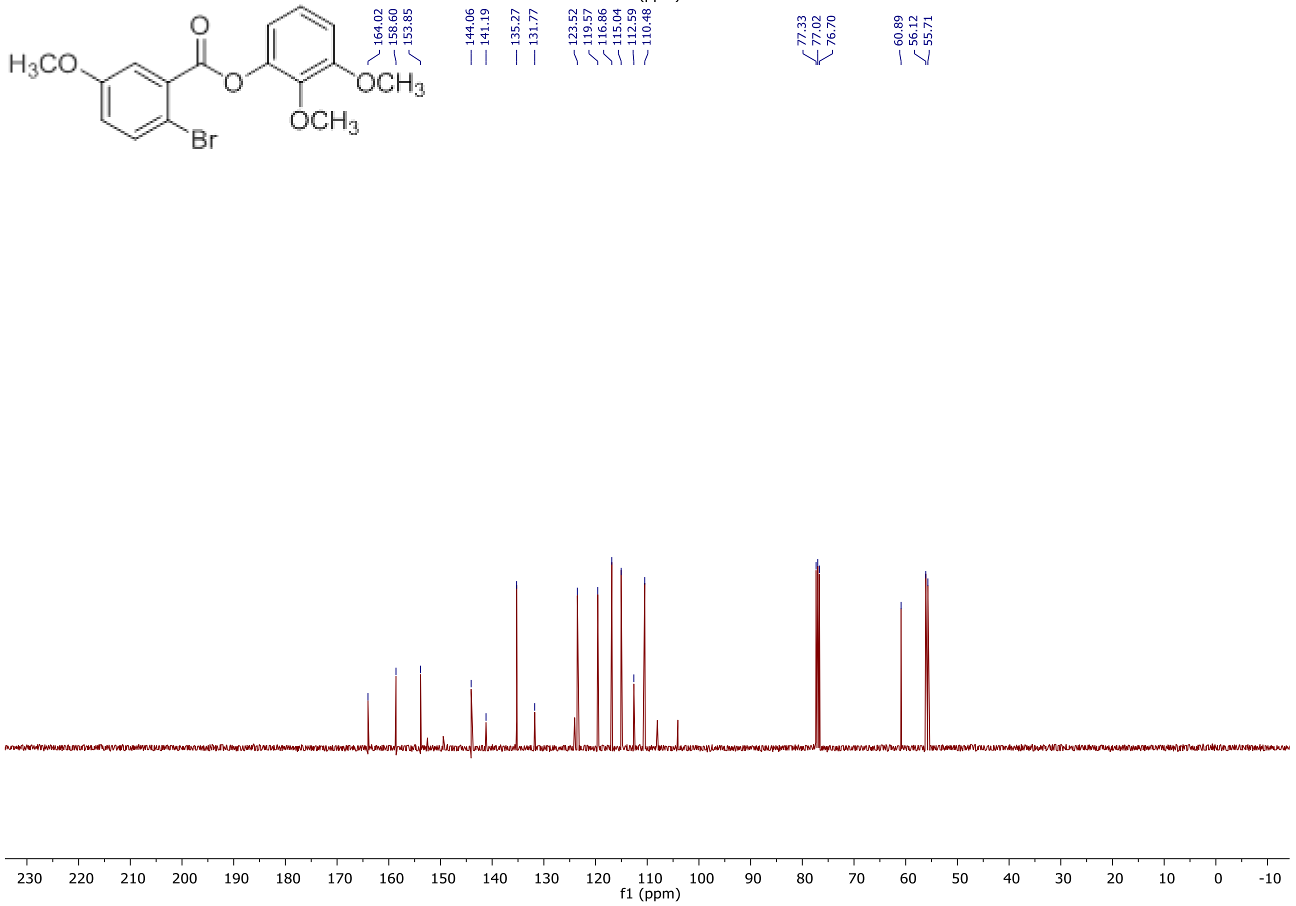
H3CO
O
O
OCH3
OCH3
Br
164.02 158.60 153.85 144.06 141.19 135.27 131.77 123.52 119.57 116.86 115.04 112.59 110.48
77.33 77.02 76.70
60.89 56.12 55.71
f1 (ppm)

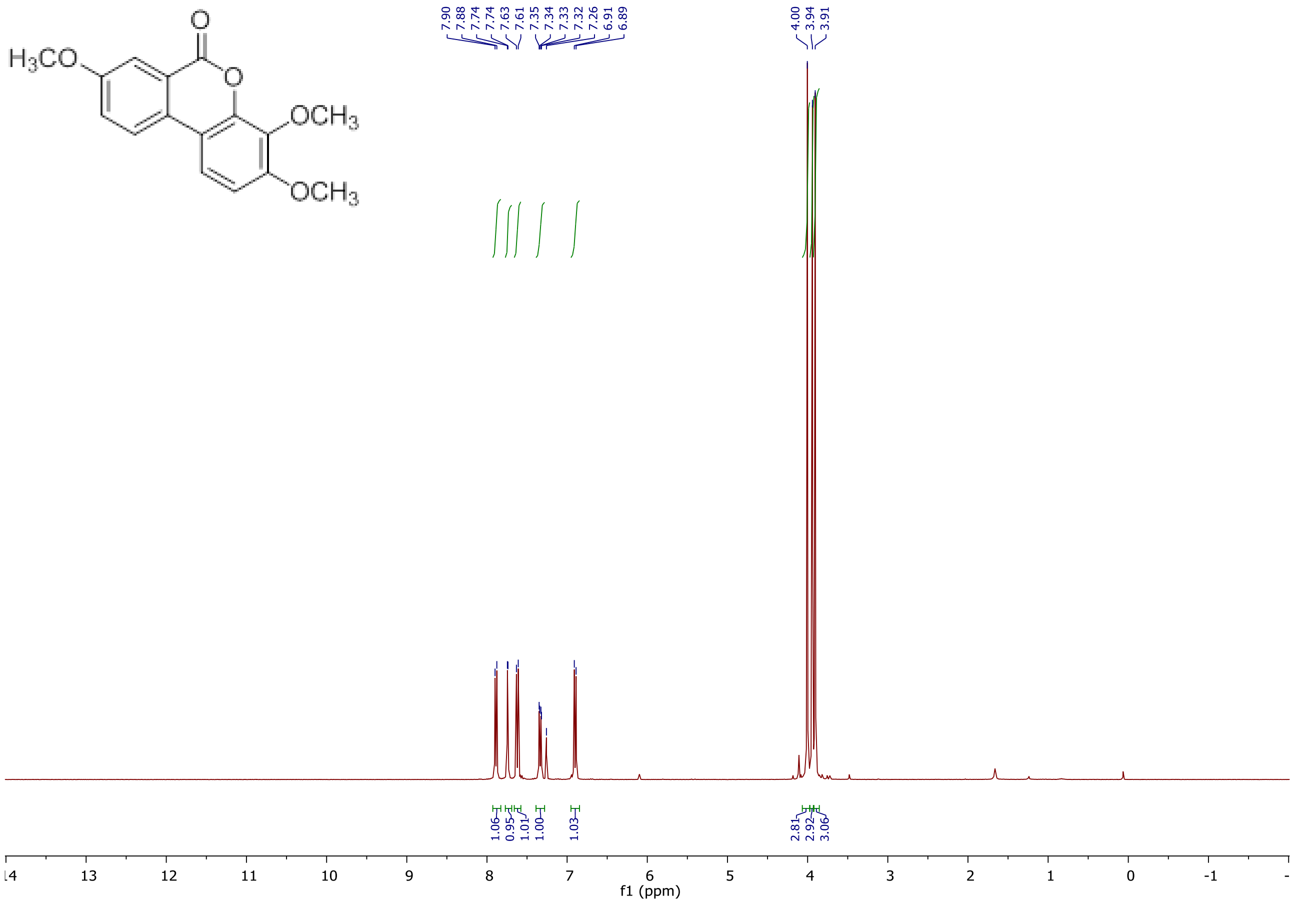
H3CO
OCH3
OCH3
7.90
7.88
7.74
7.74
7.63
7.61
7.35
7.34
7.33
7.32
7.26
6.91
6.89
4.00
3.94
3.91
1.06
0.95
1.01
1.00
1.03
2.81
2.92
3.06
f1 (ppm)

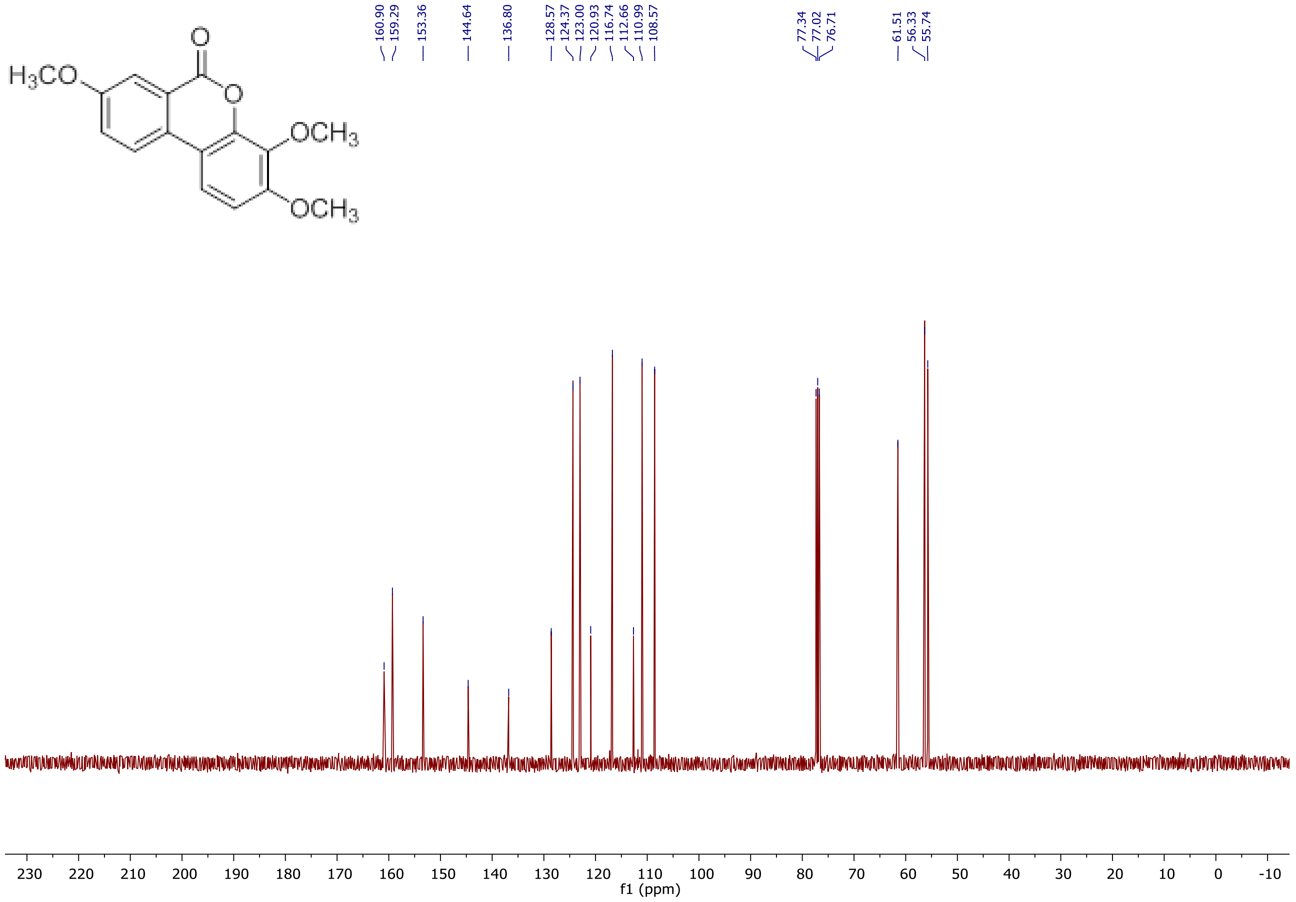
H3CO
OCH3
OCH3
160.90
159.29
153.36
144.64
136.80
128.57
124.37
123.00
120.93
116.74
112.66
110.99
108.57
77.34
77.02
76.71
61.51
56.33
55.74
f1 (ppm)

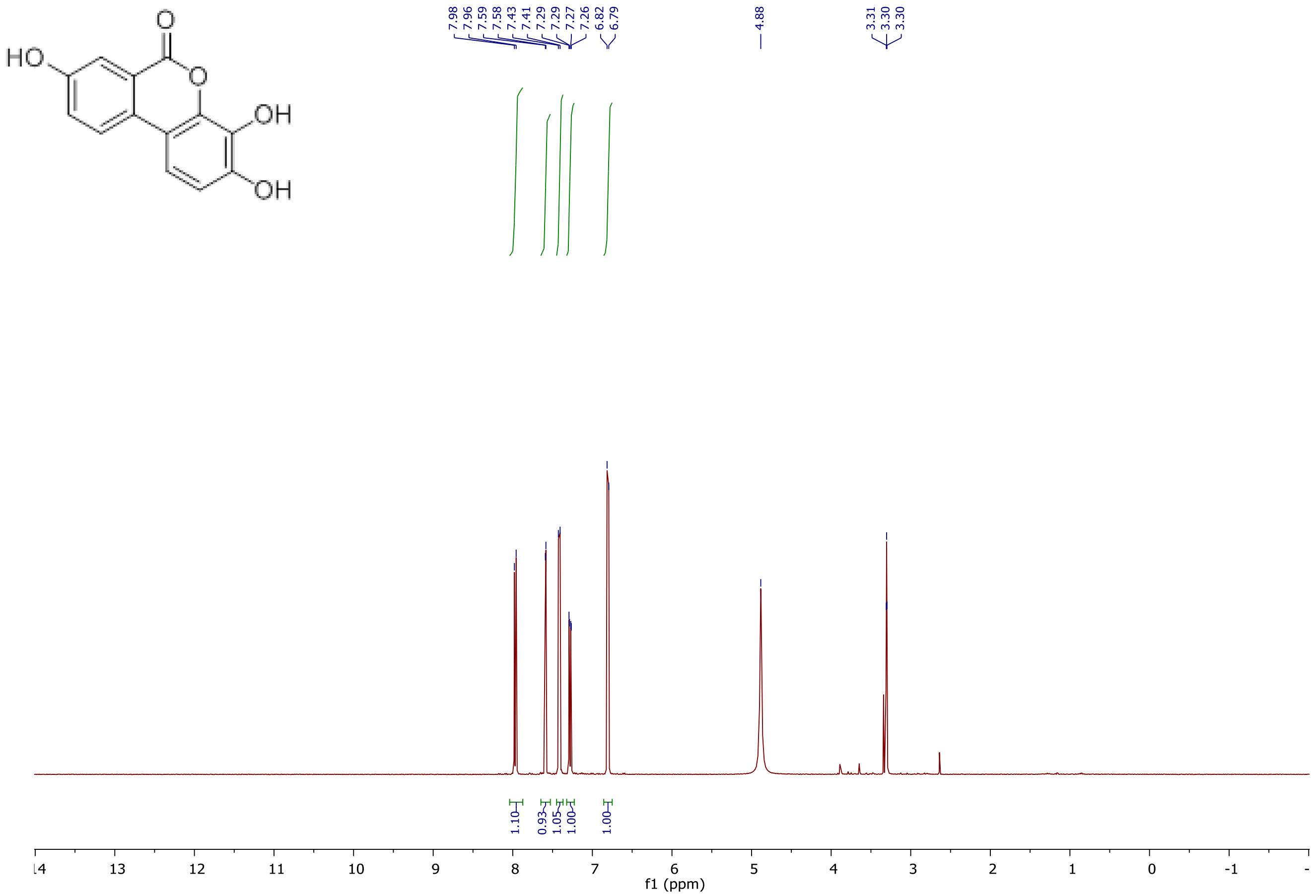
HO
O
OH
OH
7.98
7.96
7.59
7.58
7.43
7.41
7.29
7.29
7.27
7.26
6.82
6.79
4.88
3.31
3.30
3.30
1.10
0.93
1.05
1.00
1.00
f1 (ppm)

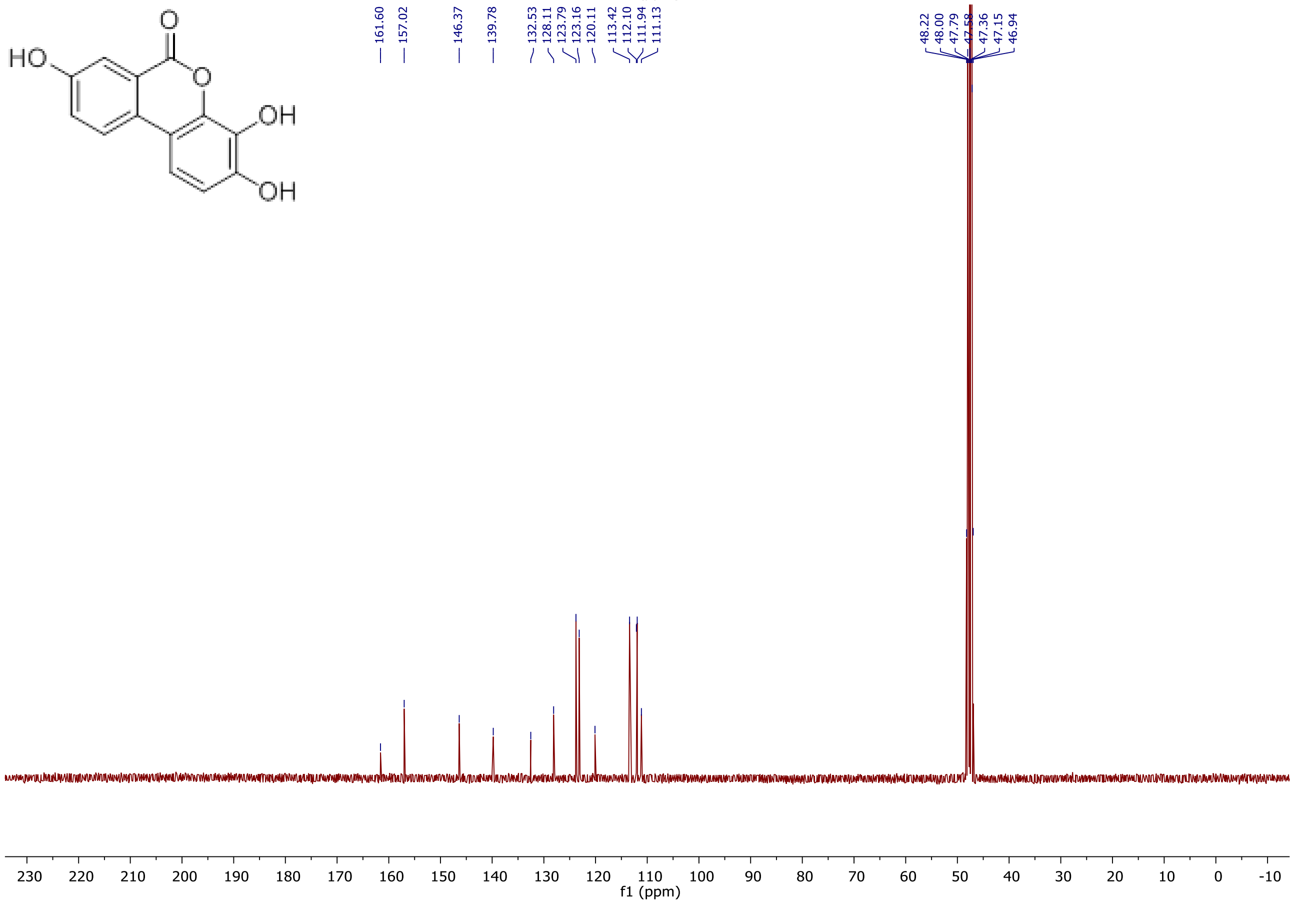
HO
O
OH
OH
161.60
157.02
146.37
139.78
132.53
128.11
123.79
123.16
120.11
113.42
112.10
111.94
111.13
48.22
48.00
47.79
47.36
47.15
46.94
f1 (ppm)
